# Bis(10‐oxacorrole) with Fused Pyridine as a Linker: Synthesis, Structure, and Interaction between the Subunits in the Neutral, Mono‐, and Dicationic States

**DOI:** 10.1002/chem.202501085

**Published:** 2025-04-27

**Authors:** Qin Liu, Sha Li, Guoru Chen, Xiaofang Li, Aleksandra Gałązka, Michał J. Białek, Piotr J. Chmielewski

**Affiliations:** ^1^ Key Laboratory of Theoretical Organic Chemistry and Functional Molecules Ministry of Education School of Chemistry and Chemical Engineering Hunan University of Science and Technology Xiangtan Hunan 411201 China; ^2^ Department of Chemistry University of Wrocław 14 F. Joliot‐Curie Wrocław 50 383 Poland

**Keywords:** antiaromaticity, aromaticity, biradicaloids, porphyrinoids, radicals

## Abstract

Pyridine‐fused bis(oxacorrole)s are obtained by a Hantzsch‐type cyclization using arylaldehydes and 3‐amino‐10‐oxacorrole. Spectroscopic characteristics, corroborated by density functional theory (DFT) calculations, indicate aromaticity of the dimer and strong interaction between the subunits. Electrochemical and spectroelectrochemical analyses indicate facile access to cation radicals and dicationic species. The oxidized forms are characterized by electronic and electron spin resonance (ESR) spectroscopies as well as by DFT calculations. Monocations give rise to strong absorption bands in the near infra‐red (NIR) region between 1500 and 2500 nm, while dications are characterized by a series of absorptions between 1000 and 2200 nm. ESR and variable temperature (VT) nuclear magnetic resonance (NMR) experiments indicate the presence of singlet‐triplet spin equilibria for the solution of the dication. For the dication of bis(oxacorrole) in the singlet ground state, neither aromaticity nor antiaromaticity has been detected by low‐temperature ^1^H NMR and gauge independent atomic orbital (GIAO) calculations despite delocalization of π‐electrons.

## Graphical abstract

A family of aromatic pyridine‐fused bis(oxacorrole) nickel(II) complexes was obtained starting from antiaromatic norcorrolatonickel(II). Interaction between the subunits of the dimer allowed observation of four‐step oxidation, giving rise to strong absorptions in the NIR region.

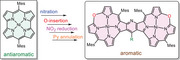

## 1 Introduction

**Figure 1:**
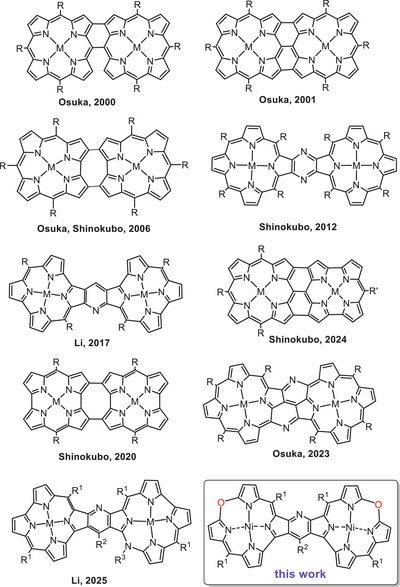
Fused bis(porphyrinoids).

**Scheme 1:**
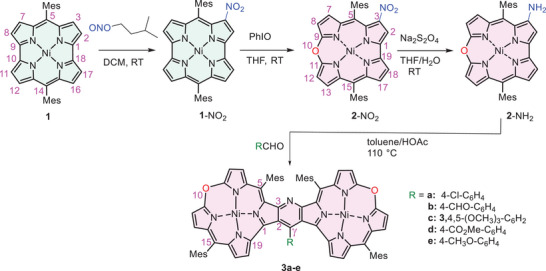
Synthetic path leading from antiaromatic norcorrole **1** to pyridine‐fused bis(oxacorrole) **3**.

Porphyrinoids, porphyrin‐like macrocycles comprising smaller hetero‐ or carbocyclic moieties, constitute a class of macrocycles with conjugated π‐electrons forming aromatic, nonaromatic, or antiaromatic systems. The character of the conjugation depends on the number of delocalized electron pairs (viz. Hückel's rule) and the size and shape of the macrocyclic skeleton.^[^ ^1^, ^2^, ^3^, ^4^, ^5^, ^6^, ^7^, ^8^, ^9^, ^10^, ^11^, ^12^, ^13^, ^14^, ^15^, ^16^ ^]^ These structural features determine the optical and redox properties of the porphyrinoids, allowing their fine‐tuning or essential modifications. There are also many structurally different oligomers with a direct bonding between the porphyrinoid subunits,^[^ ^17^, ^18^, ^19^, ^20^, ^21^, ^22^, ^23^ ^]^ but the strongest interaction between the subunits is expected for the systems in which macrocyclic rings are fused to each other or to the linking ring or rings securing conjugation (Figure 1).^[^ ^24^, ^25^, ^26^, ^27^, ^28^, ^29^, ^30^, ^31^, ^32^, ^33^, ^34^ ^]^ Such an interaction results in the profound alteration of the macrocyclic subunits' electronic structure, leading to changes in spectroscopic, magnetic, and redox properties of the porphyrinoid subunits. Those subunits may be of the same character, i.e., both aromatic or antiaromatic in homodimers, while a fused heterodimer consisting of aromatic porphyrin and antiaromatic norcorrole has also been reported recently by Shinokubo^[^ ^31^ ^]^ (Figure 1). In our laboratories, the heterodimers consisting of pyridine‐fused antiaromatic norcorrolatonickel(II) and aromatic 10‐azacorrole nickel(II) have been synthesized and reported recently (Figure 1).^[^ ^35^ ^]^ As we have shown, the antiaromatic norcorrolatonickel(II) **1** ^[^ ^36^ ^]^ (Scheme 1) can be efficiently transformed into aromatic zwitterionic corrole^[^ ^37^ ^]^ or 10‐arylazacorrole^[^ ^38^, ^39^ ^]^ nickel(II) complexes by oxidative ring expansions. Notably, owing to its antiaromatic character, norcorrole **1**,^[^ ^36^ ^]^ easily obtainable on a gram scale, can also be easily modified in many substitution or addition reactions.^[^ ^40^, ^41^, ^42^, ^43^, ^44^, ^45^, ^46^, ^47^, ^48^, ^49^, ^50^, ^51^, ^52^, ^53^, ^54^, ^55^, ^56^, ^57^, ^58^, ^59^ ^]^ As we have shown, the oxidative ring expansion leading to the azacorrole is also effective for the substituted norcorroles.^[^ ^60^ ^]^ That, in turn, may be exploited in synthesizing analogously substituted aromatic systems with a potential for further application.

We have recently reported the synthesis of the pyridine‐fused bis(10‐arylazacorrole)s starting from either the pyridine‐fused bis(norcorrole) or from the appropriately functionalized monomeric azacorrole.^[^ ^35^ ^]^ In this paper, we reported the synthesis and characterization of pyridine‐fused bis[10‐oxacorrolatonickel(II)] system (Figure 1), starting from the conversion of the easily obtainable 3‐nitro‐norcorrolatonickel(II)^[^ ^54^ ^]^ into analogously substituted 10‐oxacorrole. The 10‐oxacorrole is a macrocycle that closely resembles porphyrin by having a similar cavity size and a doubly negative charge of the coordination core.^[^ ^61^, ^62^ ^]^ Thus, it stabilizes complexes of divalent metals such as copper(II), nickel(II), and palladium(II), unlike regular corroles of triply negative core.^[^ ^37^, ^63^ ^]^ Although its aromaticity in the free base state is much less pronounced than that of corroles or the other 10‐heterocorroles,^[^ ^62^ ^]^ the nickel(II) complexes of oxacorroles indicate a diatropic current similar to that of 10‐azacorrolatonickel(II)^[^ ^36^, ^38^, ^39^ ^]^. Here, we intended to obtain dimeric systems consisting of pyridine‐fused oxacorroles to elucidate the effects of the *meso*‐oxygen presence on the structural, spectral, and redox properties of the interacting, weakly aromatic subunits.

## 2 Results and Discussion

The synthesis of the pyridine‐fused bis(oxacorrole) **3** (Scheme 1) mimicked that of the analogous bis(norcorrole),^[^ ^30^ ^]^ involving an arylaldehyde and NH_2_‐substituted macrocycle in a key step of Hantzsch‐type cyclization. This synthetic step is, by necessity, regioselective, owing to the reaction mechanism proceeding through ArCH‐linked bis(amino‐macrocycle). In turn, the positioning of NH_2_ at C3 in the monomer is related to the regioselectivity of the nitration of norcorrole **1,** occurring solely at this site.^[^ ^54^ ^]^ On the other hand, the insertion of an oxygen atom into the macrocycle may essentially occur in two positions: either between C1 and C18 or between C9 and C10 of **1**‐NO_2_ (Scheme 1). Nevertheless, the only product of the reaction with iodosylbenzene was **2**‐NO_2_, indicating selectivity of the ring expansion occurring solely at the C9‐C10 bipyrrole moiety. It appeared also that the nitro group facilitates the norcorrole → oxacorrole transformation, allowing application of a less aggressive oxygen‐transfer agent than in the original synthesis of oxacorrole and giving a slightly higher outcome (about 60% reaction yield of **2**‐NO_2_ with PhIO versus 50% for **2** reacting with *m‐*chloro‐peroxybenzoic acid^[^ ^36^ ^]^). The final dimerization process was carried out in the presence of air as a one‐pot reaction. However, it involved two steps: condensation, with leaving H_2_O followed by the oxidative pyridine annulation.^[^ ^30^ ^]^ The moderate yield of 36–51% of the dimerization step did not depend significantly on the substituents present in the aryl aldehyde applied for the condensation.

**Figure 2:**
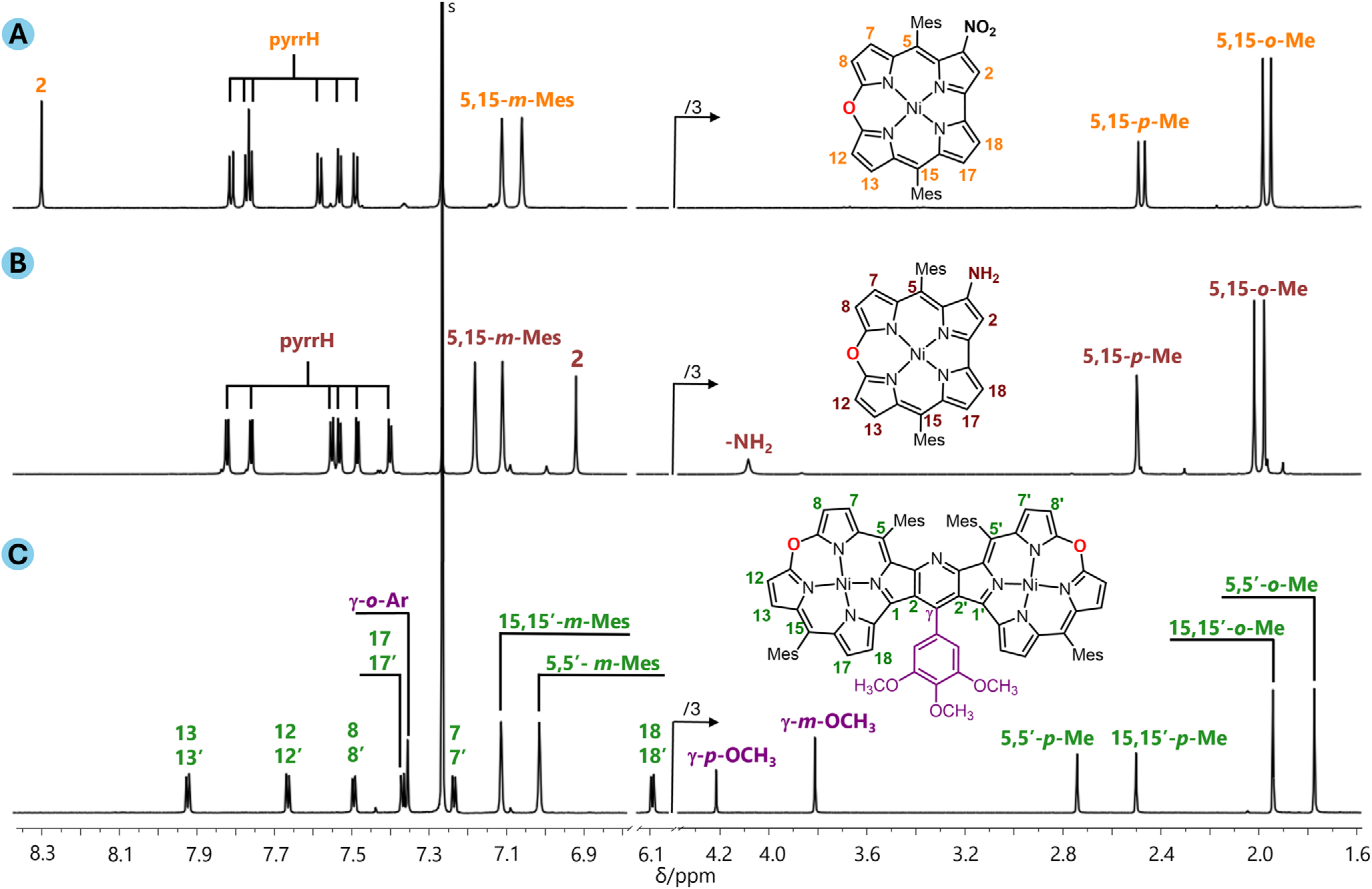
^1^H NMR spectra (500 MHz, CDCl_3_, 300 K) of **2**‐NO_2_ (A), **2**‐NH_2_ (B), and **3c** (C).

All the new systems were characterized by high‐resolution mass spectrometry (HRMS), 1D and 2D homo‐ and heteronuclear nuclear magnetic resonanse (NMR) experiments, optical spectroscopy, and for monomeric **2**‐NO_2_ and for the dimers **3a** and **3c**, also by single‐crystal X‐ray diffraction analyses.^[^ ^64^ ^]^ Mass spectra confirmed the composition of the products, in particular, the dimeric character of **3** (see Experimental Section in Supporting Information for the details). ^1^H NMR indicated aromaticity and symmetry of both monomeric 10‐oxacorrole derivatives and all the pyridine‐fused dimers (Figure 2). The spectra of **2**‐NO_2_ and **2**‐NH_2_ were interpreted as those of the *C*_s_ point symmetry group molecules by the presence of one substituent on the oxacorrole ring. Thus, the six β‐pyrrole protons gave rise to three pairs of doublets in the region of δ 7.3–7.9 ppm for both monomers, while 2‐H appeared as a singlet near δ 8.3 ppm for **2**‐NO_2_ or at δ ≈ 6.9 ppm for **2**‐NH_2_, reflecting strong influence of the electron donating‐withdrawing properties of the substituent at C3 on the shielding of the adjacent position. The spectra of all dimers **3** revealed a twofold symmetry of the molecules, consisting of one set of six pairwise coupled doublets spread over a region of δ 6.1–8.0 ppm, two sets of *meso*‐Mes signals (*meta*‐H, *o‐*Me, and *p*‐Me), and the signals of the aryl located at C_γ_ of the pyridine bridge. Significantly, there was no diastereotopic differentiation of the *m*‐H or *o‐*Me of the mesityls, suggesting either planarity of the dimer or fast on the NMR timescale libration of the subunits with respect to a mean plane of the molecule. Notably, a significant upfield shift of 18‐ and 18′‐H in comparison to the other pyrrole β‐protons of **3**, was due to the shielding effect of the aromatic current of the aryl substituent at the pyridine C_γ_.

**Figure 3:**
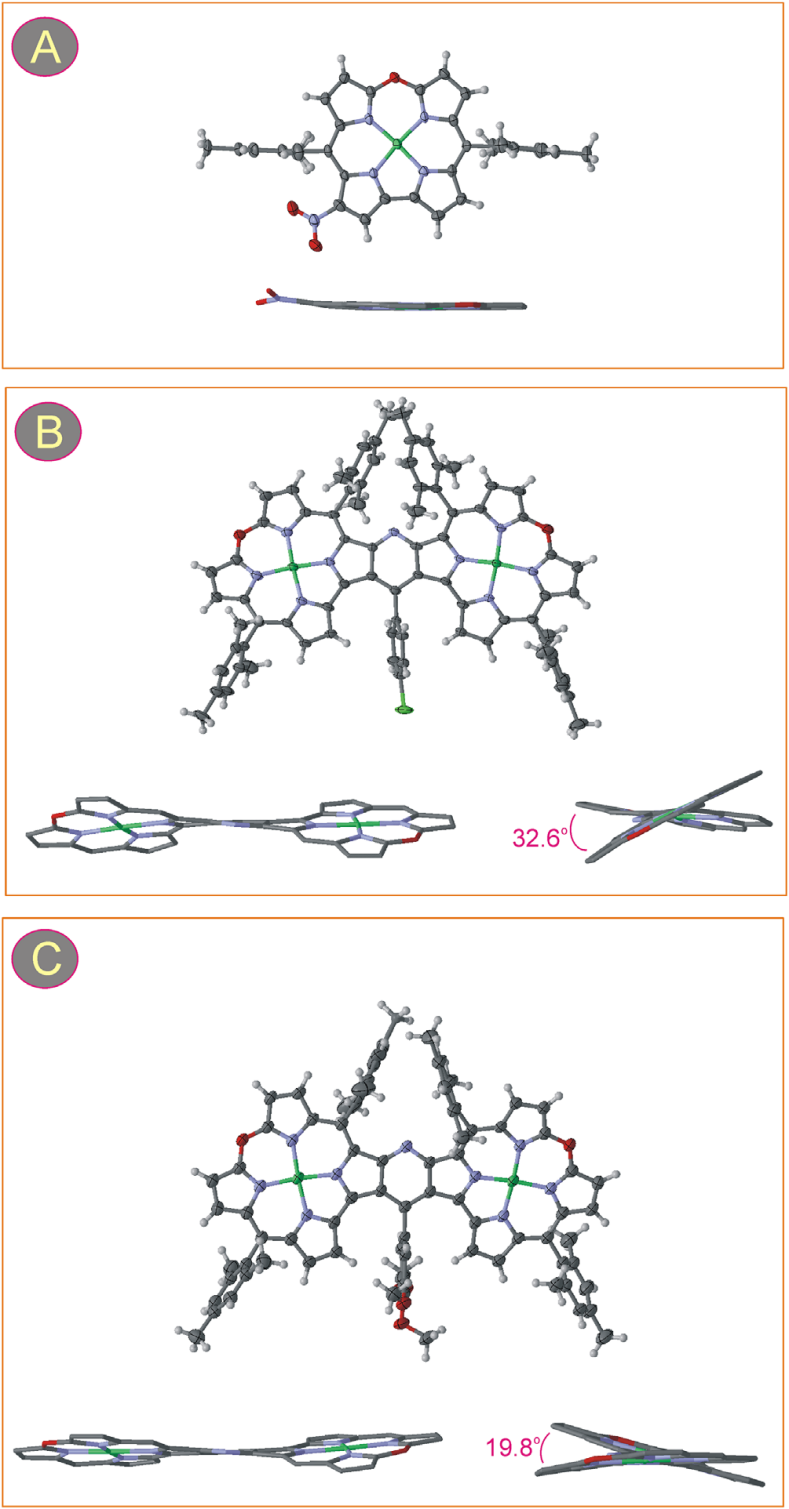
Perspective aerial views with 50% displacement ellipsoids and stick representations of two side views of the molecular structures of **2**‐NO_2_ (A), **3a** (B), and **3c** (C). All solvent molecules present in the crystal lattices are omitted. In the side views, all hydrogen atoms and aryl substituents are removed for clarity. Only one enantiomer is shown for **3a** and **3c**. The numbers given in one of the side views are the dihedral angles between oxacorrole mean planes in the dimers.

**Figure 4:**
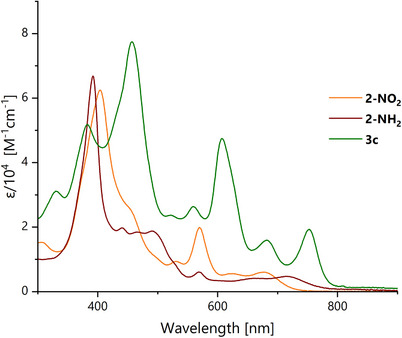
UV/Vis/NIR absorption spectra (DCM, 298 K) of **2**‐NO_2_, **2**‐NH_3_, and **3c**.

The crystal diffraction analysis for monomeric oxacorrole **2**‐NO_2_ confirmed its molecular structure and revealed planarity of the macrocyclic ring (total out‐of‐plane deviation 0.2 Å), including the central metal ion coplanar with the 4 N donor system of the porphyrinoid (Figure 3, 4). The nitro group deviates slightly from the mean plane of the macrocycle with a dihedral angle of about 14°.

The crystal structures of dimeric **3a** and **3c** also comprise almost planar macrocyclic subunits which, however, are not coplanar with each other (Figure 3). The dihedral angles between the mean planes of oxacorroles are 32.6° for **3a** and 19.8° for **3c**. A relatively big difference between these angles cannot be accounted for merely by different steric conditions in the dimers. Although the twist of the molecule appeared to be caused by the repulsion of the mesityl substituents at C5 and C5', a part of the distortion was likely due to crystal packing forces. Nevertheless, the skewing of **3** is much less pronounced than that of the pyrazine‐fused oligoporphyrins in which macrocyclic subunits and the whole molecule are profoundly distorted.^[^ ^34^, ^65^ ^]^ Due to the twisting observed for **3a** and **3c**, both molecules were chiral in the solid state, but they crystallized in the centrosymmetric space groups as racemates.

Electronic spectra of the monomeric oxacorrole derivatives resemble those of porphyrins and azacorroles rather than corrole, with Soret‐like bands near 400 nm and a series of weaker Q bands in the region between 450 and 750 nm (Figure 4). The spectra differ in the exact position of the bands, which reflects a difference in the electron‐donating and electron‐withdrawing properties of the substituents. In the spectrum of **2**‐NH_2_, the lowest energy transition is bathochromically shifted by 60 nm with respect to that of **2**‐NO_2_, while the Soret band of the amine‐substituted oxacorrole appears at about 15 nm lower wavelength than that of the nitrated derivative. A considerable bathochromic shift of the Q‐band and splitting of the Soret band are observed for the dimeric system **3**. The increased intensities of almost all absorption with respect to those observed for monomers and overall alteration of the spectral shape reflect the dimeric character of **3** and strong electronic interaction between the subunits (Figure 4).

**Figure 5:**
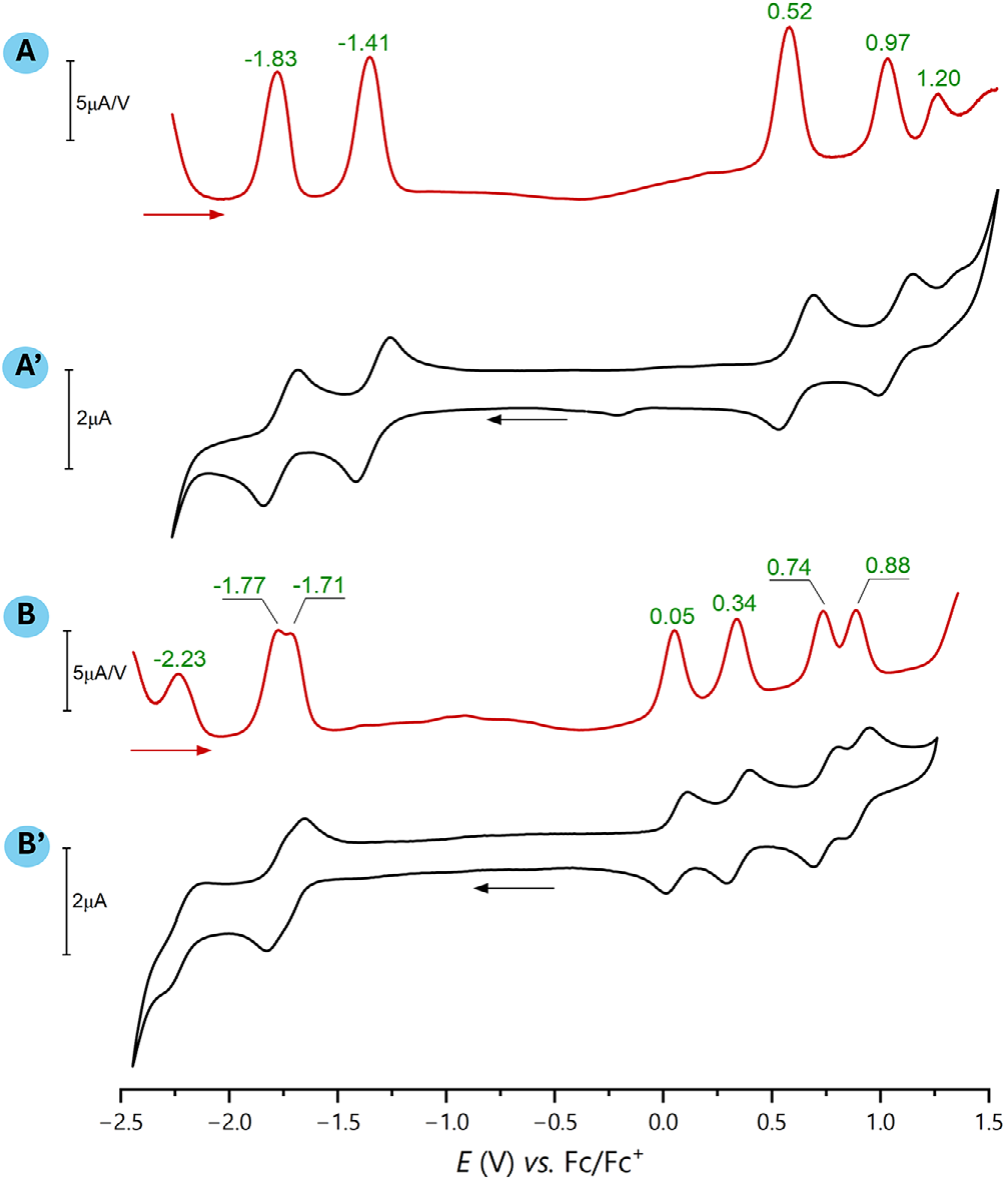
DPV (red traces) and CV (100 mV s^−1^, black traces) of **2**‐NO_2_ (A and A') and **3c** (B and B'). Conditions: solvent, DCM; supporting electrolyte, tetrabutylammonium hexafluorophosphate (0.3 M); working electrode, glassy carbon disc; reference electrode, Ag/Ag^+^ (liquid junction); auxiliary electrode, platinum rod. The horizontal arrows indicate the direction of the potential sweep. The green numbers associated with DPV scans are peak potentials in volts.

Redox properties of the new systems were studied in dichloromethane (DCM) through cyclic (CV) and differential pulse (DPV) voltammetry using a glassy carbon electrode and Bu_4_NPF_6_ as a supporting electrolyte (Figure 5). The electrode potentials were referenced to the ferrocene/ferrocenium internal standard. All electrochemical experiments were presented in Figures S60–S66 in the Supporting Information, and data were collected in Table 1. Voltammograms of **2**‐NO_2_ revealed two reversible reductions and one reversible oxidation, followed by one quasi‐reversible and one irreversible process. Notably, both the first oxidation and first reduction steps were anodically shifted by 0.22 and 0.34 V, respectively, compared with the unsubstituted oxacorrolatonickel(II).^[^ ^36^ ^]^ Such potential shifts reflect stabilization of the reduced forms and destabilization of the oxidized forms by the presence of the strong electron‐withdrawing substituent and indicate strong influence of the NO_2_ group attached directly to the porphyrinoid ring on the energy of both highest occupied molecular orbital (HOMO) and lowest unoccupied molecular orbital (LUMO). The additional reduction event observed at −1.83 V for **2**‐NO_2_ also indicated stabilization of the reduced forms provided by electron‐withdrawing substituents. A similar strong influence has been observed previously for the antiaromatic nitrated norcorroles.^[^ ^54^ ^]^ As expected, the electron‐donating substituent NH_2_ stabilizes the oxidized forms of the oxacorrole, shifting the first oxidation potential of **2**‐NH_2_ by 0.3 V to the lower value of *E*_ox1_, and decreasing the second oxidation potential by 0.22 V in comparison with unsubstituted **2**. Dimeric species **3** were characterized by four reversible oxidations occurring between 0.05 and 0.95 V, two closely‐spaced reversible reductions at about −1.7 V, and one or two irreversible reductions below −2 V. Considering the symmetric structure of the dimers, the multiplication of the redox events with respect to the monomers indicated strong interaction between the subunits. Such an interaction was of an electronic rather than electrostatic nature, considering the relatively long distance between the consecutively formed charged sites located on the different subunits. The first oxidation was considerably facilitated by dimerization, occurring in all systems **3** at the potentials *E*_ox1_ more than 200 mV lower than in monomeric **2**. Also, the second oxidation steps were observed for the dimers at more than 0.4 V lower potentials *E*_ox2_, which were close to the first oxidation potential of **2**. The first reductions in dimers occurred at slightly higher potentials *E*_red1_ than in monomers. Thus, the electrochemical HOMO‐LUMO gap Δ*E*_HL_ = e(*E*_ox1_ − *E*_red1_) is 0.3 eV narrower in pyridine‐fused bis[10‐oxacorrolatonickel(II)] systems **3** than in monomeric 10‐oxacorrole nickel(II).

**Table 1:** Electrode potentials from electrochemical measurements for oxacorrole‐containing systems in DCM solutions

System	*E*_red1_ [V]	*E*_red2_ [V]	*E*_red3_ [V]	*E*_ox1_ [V]	*E*_ox2_ [V]	*E*_ox3_ [V]	*E*_ox4_ [V]	Δ*E*_HL_ [eV]
**2‐**NO_2_	−1.41	−1.83	–	0.52	0.97	1.20^[a]^		1.93
**2‐**NH_2_	−1.84	–	–	0.00	0.56	–	–	1.84
**2** ^[b]^	−1.75	–	–	0.30	0.78			2.05
**3a**	−1.69	−1.74	−2.19 −2.36^[a]^	0.06	0.36	0.78	0.93	1.75
**3b**	−1.66	−1.72	−2.15 −2.32	0.09	0.37	0.79	0.93	1.75
**3c**	−1.71	−1.77	−2.23	0.05	0.34	0.74	0.88	1.76
**3d**	−1.68	−1.74	−2.18 −2.37	0.07	0.35	0.77	0.92	1.75
**3e**	−1.69	−1.74	−2.19 −2.37^[a]^	0.05	0.33	0.75	0.89	1.74

^[a]^ Irreversible wave;

^[b]^ Unsubstituted oxacorrole. Data from ref. [36]

**Figure 6:**
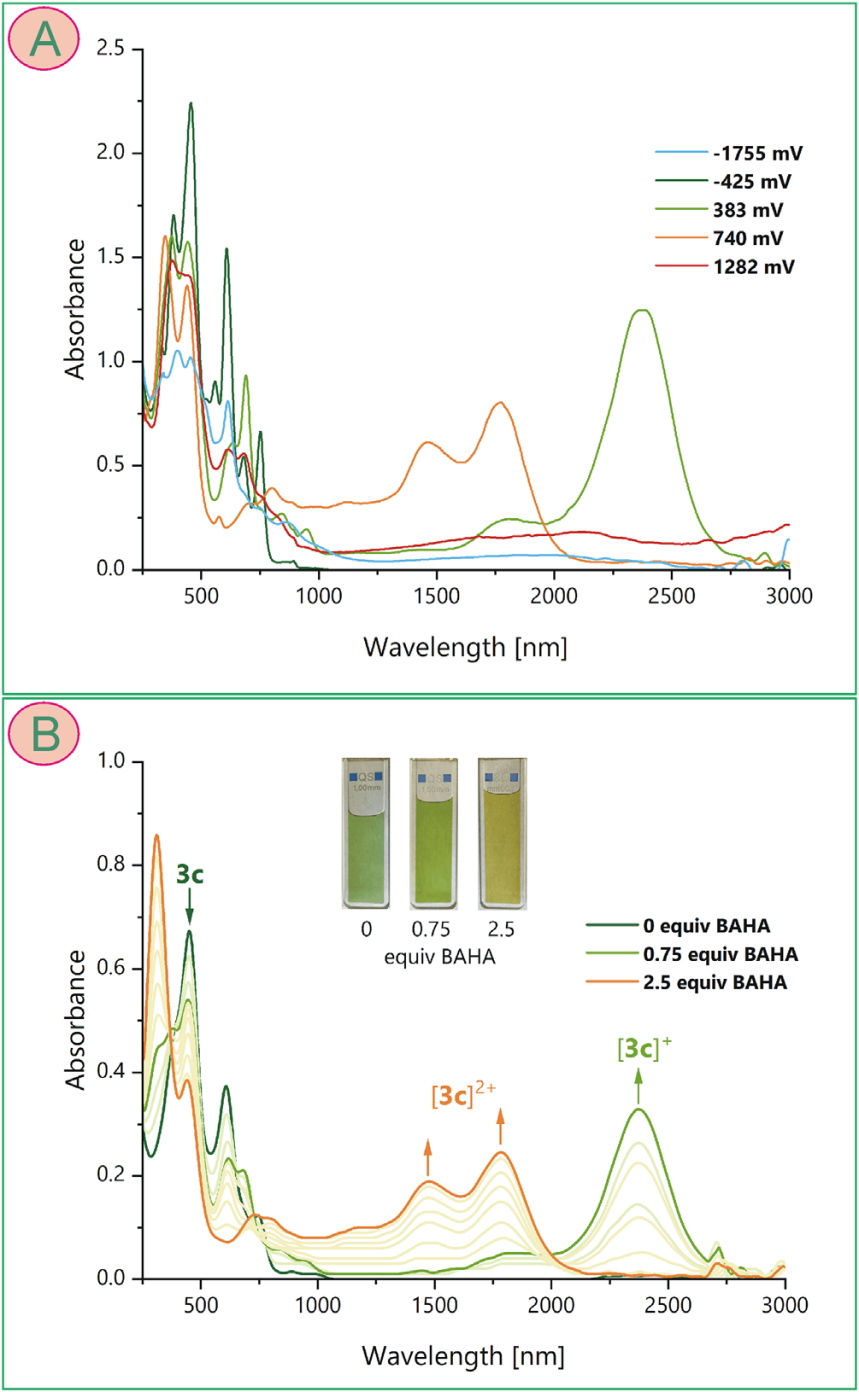
(A) UV/Vis/NIR absorption spectra recorded at the selected potentials of the working electrode upon spectroelectrochemical experiments on **3c**. Conditions: solvent, DCM; supporting electrolyte, Bu_4_NPF_6_ (0.1 M); working electrode, platinum gauze; reference electrode, Ag/Ag^+^; auxiliary electrode, platinum gauze; potential scan rate, 0.5 mV s^−1^. (B) Selected spectra recorded upon spectrophotometric titration of **3c** with tris(4‐bromophenyl)ammoniumyl hexachloroantimonate (BAHA).

Prompted by the electrochemistry results, indicating facile oxidation of the dimers, we decided to characterize the cationic forms employing electronic absorption spectroelectrochemistry, using an optically transparent thin layer electrode (OTTLE). Spectral changes were observed for **3c** in the range covering UV‐vis‐NIR from 250 to 3000 nm (Figures 6A, S67, and S68 in Supporting Information). A strong low‐energy band at about 2370 nm was formed with a weaker band at 1800 nm upon passing by the potential of the first oxidation. The formation of these NIR features was accompanied by some changes in the UV‐vis region. The resulting spectrum was assigned to the one‐electron oxidized species, i.e., cation radical [**3c^•^**]^+^. Upon increasing potential, the intensity of the radical spectrum decreased. Still, the bands disappeared completely at a potential that is considerably higher than that of the second oxidation. This may be due to the comproportionation of neutral and dicationic species, causing partial restoration of the monocationic intermediate in the electrode double layer. Nevertheless, the spectral changes in the NIR region involved a gradual increase of three bands at 1120, 1460, and 1770 nm and a considerable absorbance decrease in the visible region (at about 600 nm) upon the potential increase to 0.74 V. These spectral changes were clearly related to the second oxidation process and, thus, the spectrum was assigned to dicationic species [**3c**]^2+^. The further increase of the electrode potential allowed observation of the third oxidized form, i.e., [**3c**]^3+^ giving rise to three broad and weaker bands in the NIR region (1600, 2100, and >3000 nm) and an increase of the absorbance in the visible region between 500 and 700 nm. We also attempted to observe the reduced forms. A cathodic scan below −1.7 V resulted in the formation of a broad and weak band centered at about 1800 nm, accompanied by a considerable decrease of the Soret band and broadening of the Q‐like bands in the visible region. With the potential passing by −2 V, the NIR band disappeared irreversibly. The observed spectra were assigned to a doubly reduced form of the dimer, i.e., [**3c**]^2−^, as the first and the second reduction potentials were close to each other (−1.71 and − 1.77 V), and only one change of the spectrum shape was observed over this potential range.

For comparison, we ran an analogous spectroelectrochemical experiment for the monomeric **2**‐NO_2_ under the same conditions. The spectral changes observed upon oxidation scan in the UV‐vis region involved considerable weakening of the Soret band and broadening of the Q‐bands after passing 0.5 V of the electrode potentials (see Figure S67 in Supporting Information). Such changes indicate losing the aromatic character of the porphyrinoid and, thus, breaking the conjugation path due to an electron abstraction from the delocalized system. In the NIR region, spectral features were observed when approaching 0.8 V as ill‐defined bands near 1400 nm, tailing to 2500 nm.

**Figure 7:**
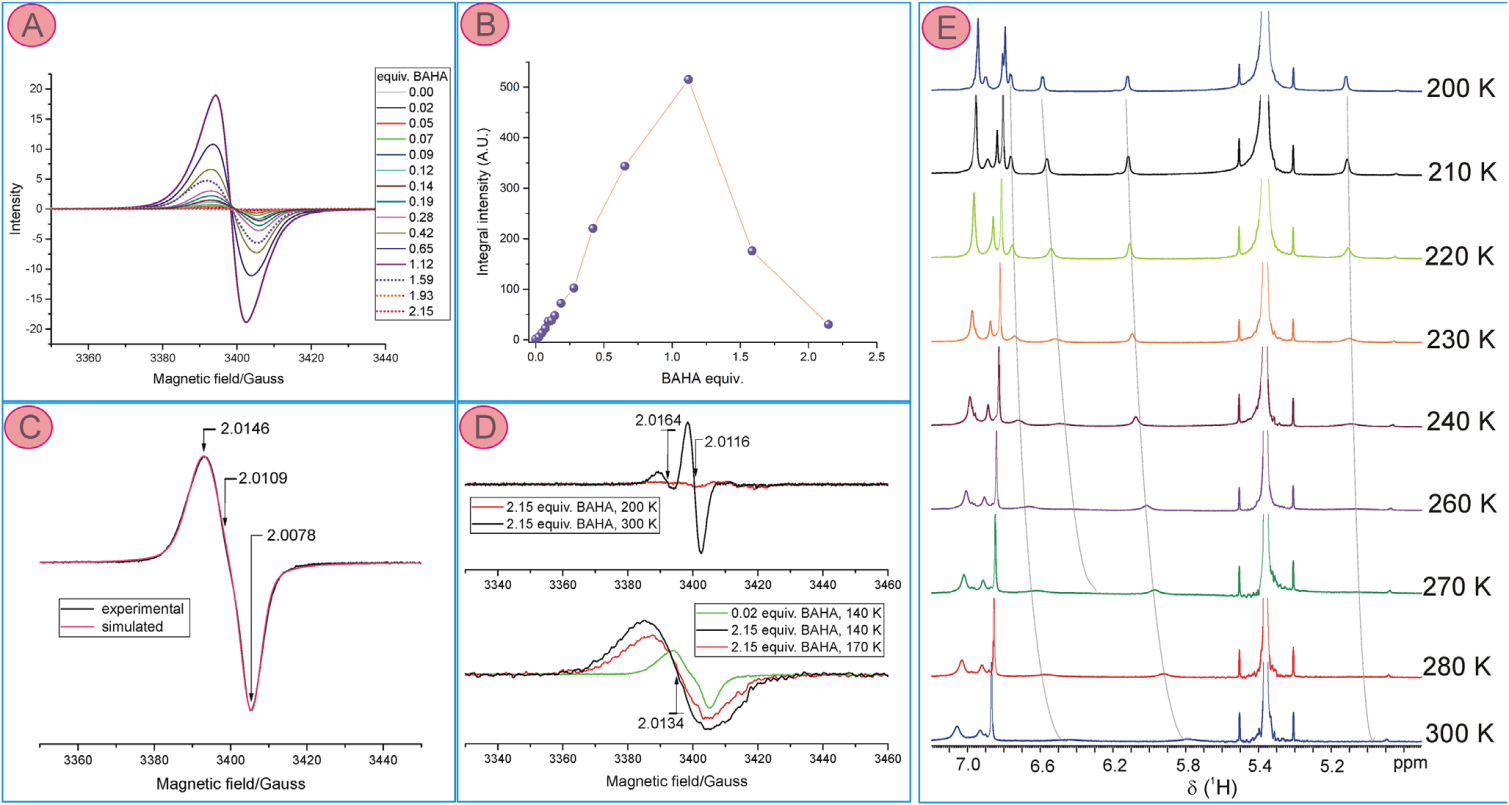
(A) Frozen solution ESR spectra (DCM, 140 K) recorded upon titration of **3c** with BAHA solution in DCM at a specified amount of the oxidant and (B) dependence of the doubly integrated ESR signal intensities on the amount of the BAHA. Both the first derivative spectra in (A) and integrated intensities were corrected for the dilution by the added volume of the titrant solution. (C) Frozen‐solution spectrum (DCM, 140 K) taken after the addition of 0.07 equiv. of BAHA to **3c** (black trace, experimental spectrum) along with spin‐Hamiltonian parameters derived from the simulation (red trace, simulated spectrum). (D) Bottom: frozen solution spectra with lowest (green trace) and highest (black and red traces) amounts of BAHA added to **3c**; top: liquid solution ESR spectra of **3c** with twofold excess of BAHA recorded at 200 K (red trace) and 300 K (black trace). (E) Selected ^1^H NMR spectra (600 MHz, CD_2_Cl_2_) recorded upon variable temperature experiment on the sample of [**3c**]^2+^ prepared from the NIR‐controlled oxidation of **3c** with BAHA followed by precipitation with hexane.

Stepwise chemical oxidation of **3** was conducted using tris(4‐bromophenyl)ammoniumyl hexachloroantimonate (BAHA, Magic Blue), and titrations were monitored using electronic absorption spectroscopy (Figure 6B) and electron spin resonance (ESR) (Figure 7A and 7B). The spectrophotometric titration of **3c** revealed analogous spectral changes as those observed in the spectroelectrochemical experiment, i.e., formation of the monocation radical, strongly absorbing in the NIR III region, and its transformation into [**3c**]^2+^, giving rise to three bands between 1000 and 1800 nm. Also, the changes in the visible region were similar to those generated in the OTTLE. Still, titration allowed visualization of the solution color changes from grass‐green of the starting **3c**, through grape‐green for the [**3c^•^**]^+^ to olive for the dication. Further oxidation was prevented by the insufficient potential of BAHA (0.7 V) with respect to the third oxidation potential of **3c** (0.74 V).

The ESR‐monitored titration of **3c** with BAHA revealed the spin state of the cationic and dicationic species. The spectra were recorded for the frozen DCM solution at 140 K (Figure 7A). The signal intensity increased up to the addition of 1 equiv. of BAHA and decreased almost completely upon the addition of 2 equiv. of the oxidant. However, the spectrum also changed its shape by a considerable broadening of the spectral line, thus the signal double integrals were taken into account upon the intensity comparison (Figure 7B). The frozen‐solution ESR spectrum of the mono‐oxidized **3c** was characterized by a low anisotropy of the Zeeman term of the spin Hamiltonian and its orthorhombic symmetry (Figure 7C). Such a low anisotropy of the **g** tensor (*g_x _* = 2.0146, *g_y_* = 2.0109, *g_z_* = 2.0078) is typical for radical rather than nickel(III) species,^[^ ^66^, ^67^, ^68^, ^69^, ^70^ ^]^ thus, a radical character of the monocation and ligand‐centered oxidation of **3** can be inferred from the ESR. The spectral changes observed at higher amounts of the added oxidant were related to the formation of the dication that potentially may adopt a paramagnetic triplet or diamagnetic singlet state, and the populations of these states depend on temperature. The strong intensity decrease upon adding two equivalents of BAHA at 140 K suggested the singlet ground state of [**3c**]^2+^ and, thus, its domination at low temperatures. On the other hand, the broader signal centered at *g* = 2.0134 observed in frozen solutions can be assigned to the triplet state, that is, the diradical form of the dication [**3c^••^**]^2+^. The liquid solution spectrum at 200 K indicated almost complete quenching of the ESR signals at low temperatures, while the room temperature spectrum consisted of the diradical signal at *g*_0 _= 2.0164, which was accompanied by the signal of monoradical at *g*_0 _= 2.0116 that partially reappeared. The presence of the radical is likely due to a partial reduction of the dication by tris(4‐Br‐phenyl)amine, that is, the reduced form of BAHA. In fact, the dioxidized form of **3c** slowly transformed into [**3c^•^**]^+^ when left in the solution at room temperature after spectrophotometric or ESR‐monitored titration with BAHA. Thus, in order to analyze the spin state of the dication, we attempted the separation of this species from the other oxidation product by precipitation of [**3c**]^2+^ with hexane immediately after NIR‐controlled preparation. Sadly, a variable temperature ESR experiment performed for the solid sample of the dication did not deliver reliable data, allowing calculation of the singlet‐triplet energy gap using the Bleaney–Bowers equation.^[^ ^71^, ^72^ ^]^ The solid material gave an unresolved signal centered at *g *= 2.0122 whose integral intensity weakly responded to the temperature variations in the range of 150–370 K. That allowed only a rough estimation of the singlet‐triplet gap as 0.5 kcal mol^−1^ with the singlet ground state (Figure S73 in Supporting Information). On the other hand, a gradual spin quenching upon temperature decrease was observed in solution by ^1^H NMR in CD_2_Cl_2_ that was complete at 200 K (Figure 7E), in line with the singlet ground state of [**3c**]^2+^. Notably, the narrowing of the signals allowed observation of the peak splitting due to H‐H coupling of the pyrrole protons as well as correlations in a COSY spectrum. Chemical shifts of the pyrrole β‐H indicated either nonaromatic or weakly antiaromatic character of the dication in the singlet state. The diamagnetism of the sample at 200 K in solution was also in accordance with the lack of the triplet signal in the ESR spectrum under such conditions (Figure 7D). Moreover, DFT calculations using CAM‐B3LYP functional, including GD3BJ dispersion correction (see Table S12 in Supporting Information) revealed that dication in the singlet state was 1.3 kcal mol^−1^ more stable than the diradical form [**3c^••^**]^2+^, in line with the experimental observations. Boltzmann population analysis indicated that such an energy difference resulted in about 1% of the triplet species at 140 K, 4% at 200 K, and more than 10% at 300 K.

**Figure 8:**
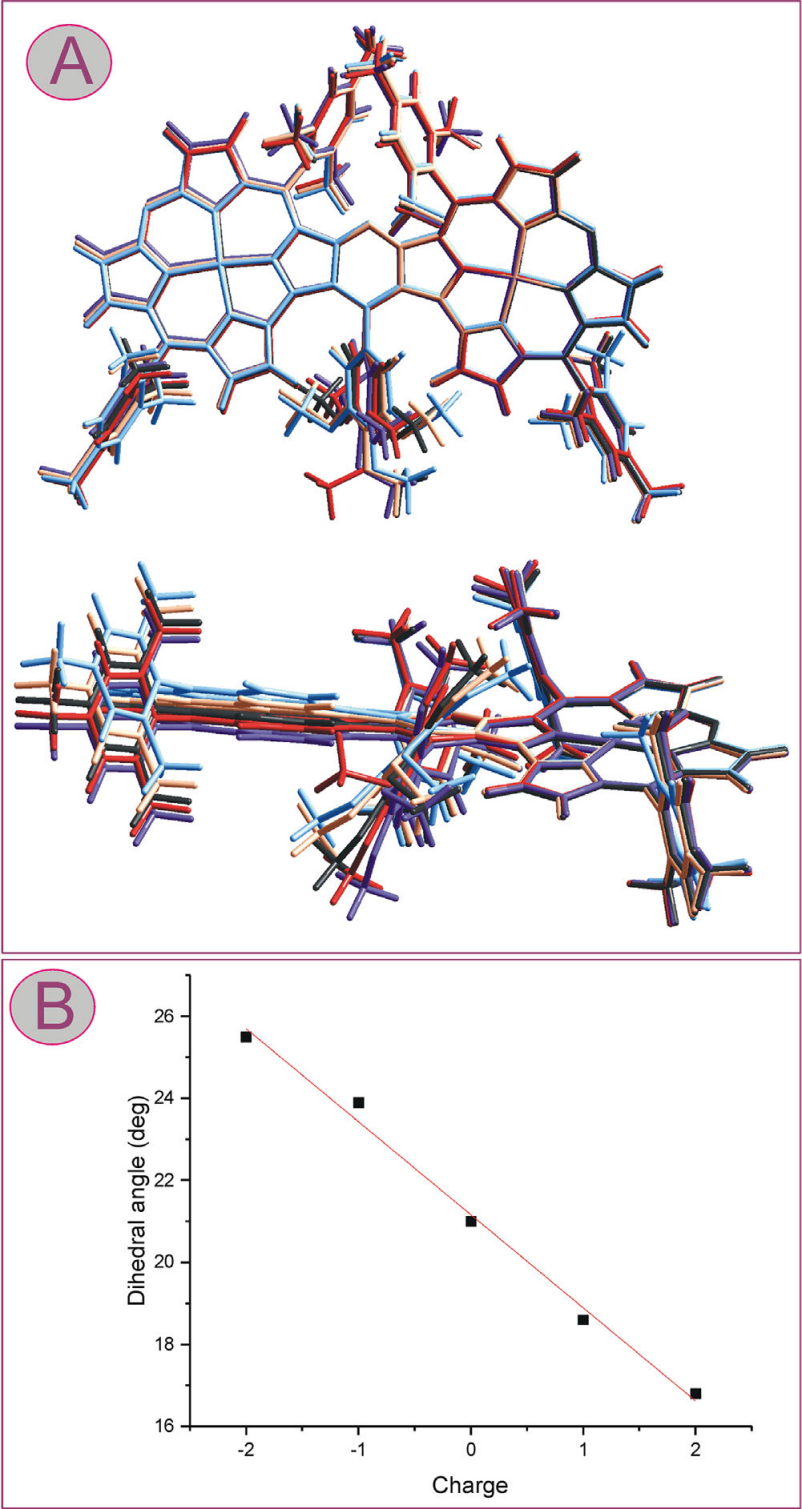
(A) Front and side views of superimposed DFT‐optimized structures of **3c** (black), [**3c^•^**]^+^ (red), [**3c**]^2+^ (violet), [**3c^•^**]^−^ (orange), and [**3c^••^**]^2‐^ (light blue). (B) Correlation of the overall charge of the species with the dihedral angles between the mean planes of the macrocyclic subunits of the dimer.

The structure, spin density distribution, and aromaticity of **3c** and its oxidation and reduction products were analyzed using DFT methods. The optimized structures were similar to each other, but with an increasing charge of the species, a monotonic decrease of the dihedral angle between the mean planes of the subunits was observed, reflecting a correlation between the overall charge of the dimer and its deviation from planarity (Figure 8).

**Figure 9:**
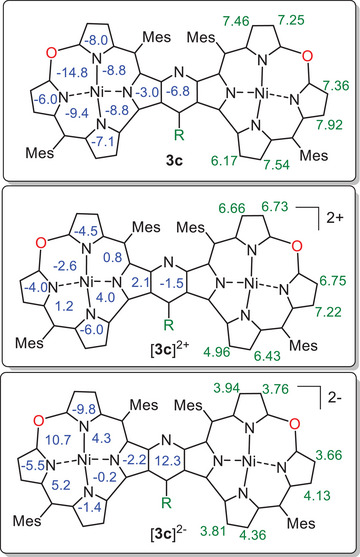
GIAO calculated NICS(0) indices (blue numbers, in ppm) and calculated pyrrole proton chemical shifts (green numbers, in ppm) for **3c**, [**3c**]^2+^, and [**3c**]^2‐^. The NICS(0) were estimated in a mean plane of the macrocycle at the midpoint of each ring. R = 3,4,5‐(MeO)C_6_H_2_.

**Scheme 2:**
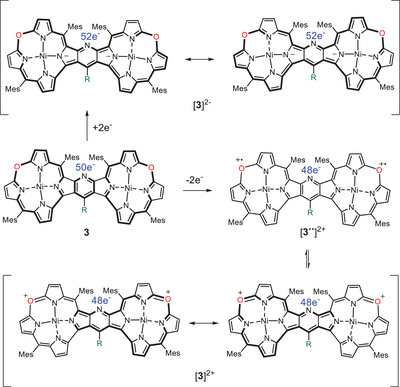
The number of electrons, including those at the *meso*‐oxygen, and schematic distribution of the electron pairs in **3** and in its two‐electron oxidation and reduction products.

The aromatic properties of the neutral and diamagnetic **3c** were corroborated by the GIAO calculation of the chemical shifts of the pyrrole protons as well as NICS indices (Figures 9 and S74). The calculated chemical shifts satisfactorily matched the experimental values, and negative values of NICS indicated the presence of diatropic current in the macrocycle subunits. Apparently, there was no significant increase of the aromatic current due to dimerization and fusing with the pyridine and both experimental and calculated chemical shifts of the pyrrole protons were, on average, close to those of the monomeric 10‐oxacorroles. Thus, the delocalization pattern in the pyridine‐fused bis(oxacorroles) may consist of two uncoupled 18πe aromatic systems not involving the aromatic bridge (Scheme 2).

On the other hand, the two‐electron oxidation of the dimer strongly reduced diatropic current, which can still be observed only locally in the pyrrole moieties, while NICS indices calculated inside the macrocycle indicated the presence of neither dia nor paratropic ring current in [**3c**]^2+^ (Figures 9 and S75). The lack of the macrocyclic ring current was in line with both calculated and experimental chemical shifts of the pyrrole β‐H resonating in a region typical for the separated pyrrole moieties (Figure 7E). Although one of these protons (18,18′‐H) resonated below δ 5 ppm, such an upfield shift was due to the shielding effect of the aromatic ring current of the substituent at the C_γ_ of the pyridine bridge rather than the macrocyclic paratropic current. The observed absence of the macrocyclic ring current may be due to a disruption of the conjugation path in the oxacorrole subunits related to the abstraction of two electrons from the *meso‐*oxygens, critical for the π‐electron delocalization. On the other hand, the diamagnetic character of the dication in the singlet state requires strong interaction between the spin sites, which, in turn, implies a long‐range delocalization that allows spin‐pairing. Intriguingly, the NICS indicated a lack of aromaticity at the bridging pyridine, which may be accounted for by its nonstandard inclusion in the “potentially paratropic” delocalization path of the 24πe system in [**3c**]^2+^ (Scheme 2).

Although we were unable to separate the two‐electron reduced species [**3c**]^2‐^, the electronic spectrum with the weak but notable broad band at about 2000 nm observed upon reduction of **3c** (Figure 6A, blue trace) suggested the antiaromatic character of the dianion. The GIAO calculations indeed indicated that a weak paratropic macrocyclic ring current may occur in the dianion, which was inferred from positive values of NICS in the middle of the six‐membered rings inside the macrocycle (Figure 9 and S76). On the other hand, the indices are rather small and, as in the case of the dication, the local aromaticity of the pyrroles was apparent from the negative NICS values. Nevertheless, the highest positive value of NICS(0) at the midpoint of the bridging pyridine may suggest its inclusion into the 40πe delocalization path in the dimer that was responsible for the presence of the paratropic current upon the addition of two electrons (Scheme 2).

**Figure 10:**
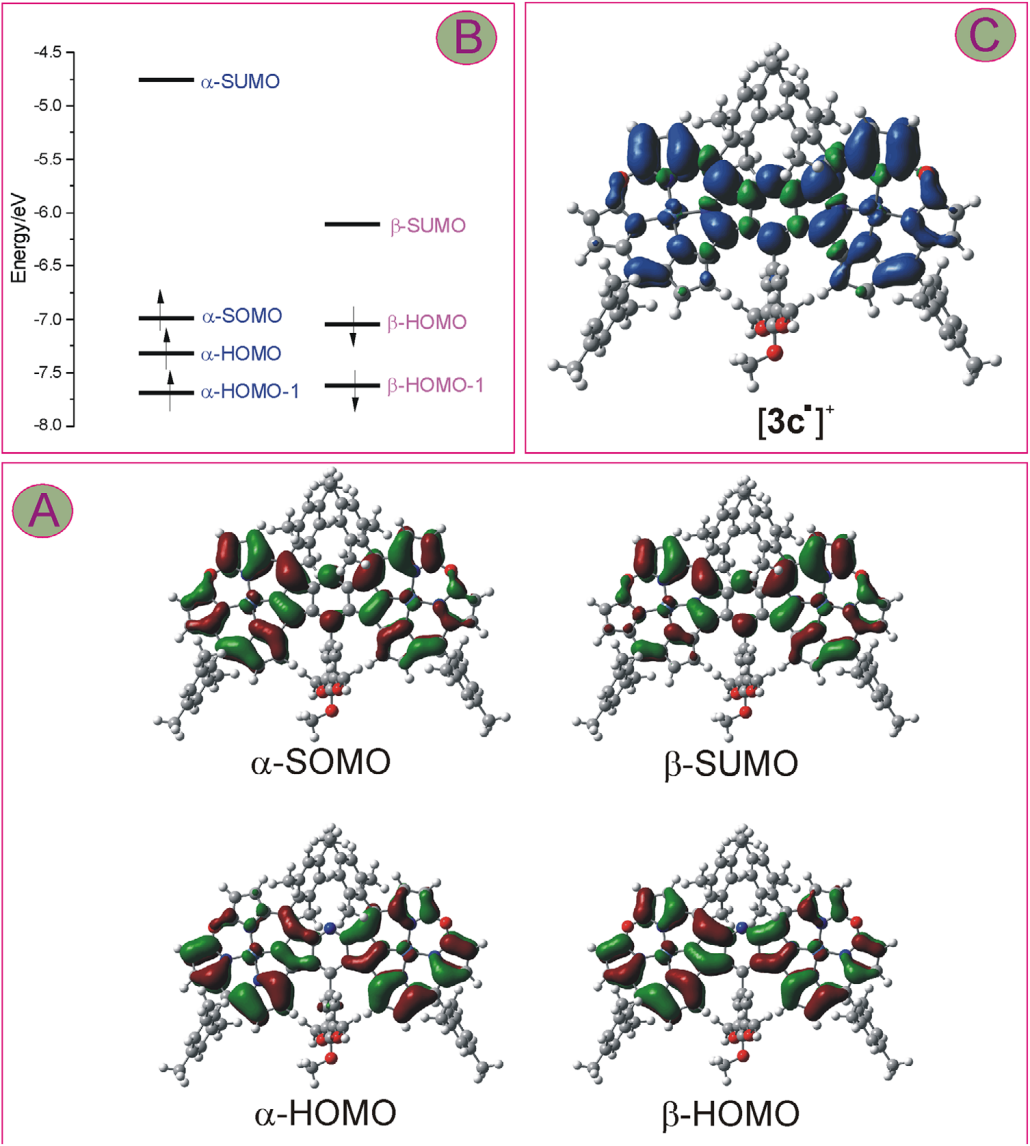
Frontier orbitals (surface isovalue 0.02) calculated for [**3c** ^•^]^+^ (A) and their energies (B). In the panel (C), the spin distribution over the molecular skeleton of the cation radical [**3c** ^•^]^+^ is presented (surface isovalue 0.0004).

**Figure 11:**
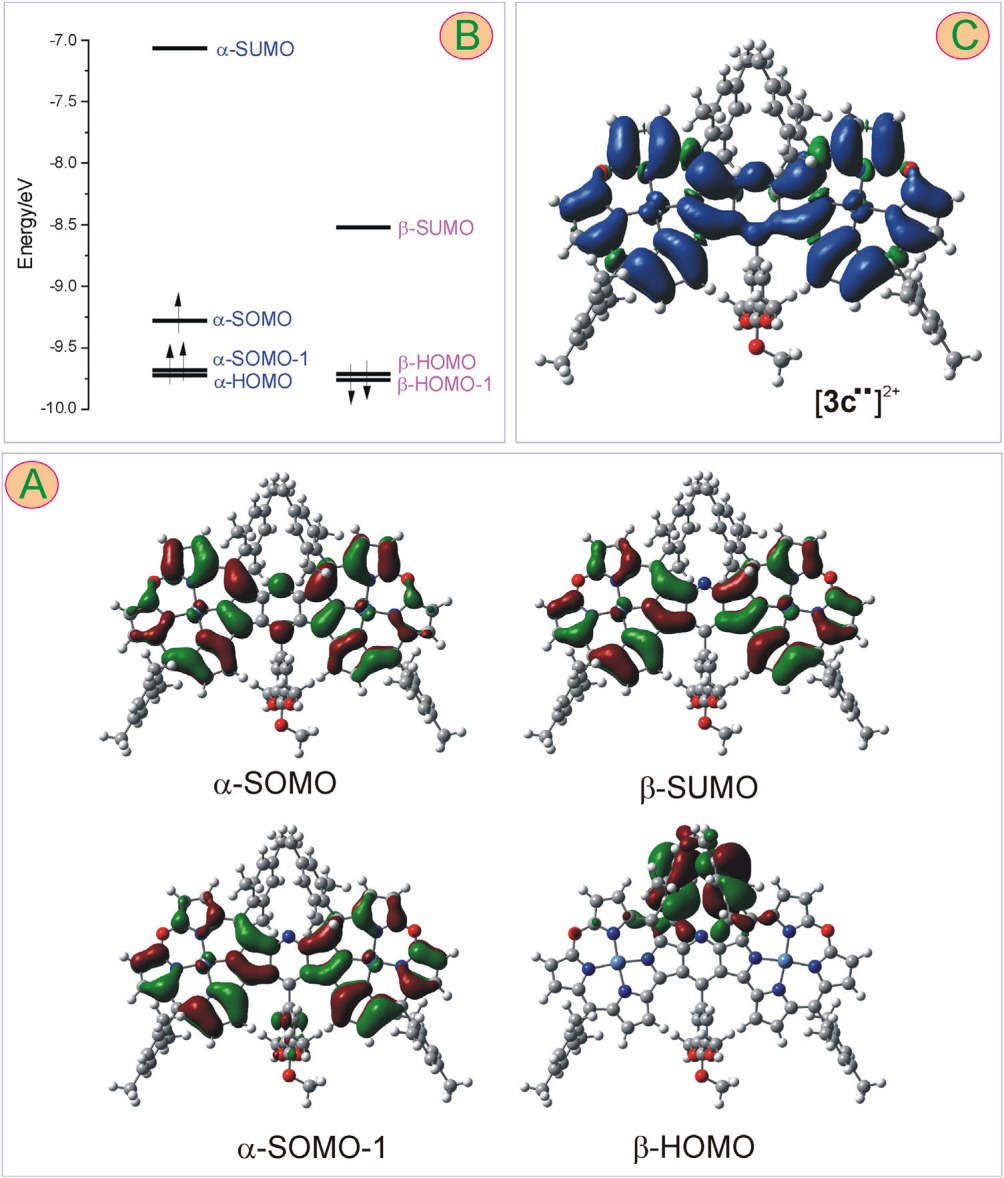
Frontier orbitals (surface isovalue 0.02) calculated for [**3c** ^••^]^+^ (A) and their energies (B). In the panel (C), the spin distribution over the molecular skeleton of the cation radical [**3c** ^••^]^+^ is presented (surface isovalue 0.0004).

DFT calculations for the paramagnetic oxidation products of **3c**, i.e., radical [**3c^•^**]^+^ and diradical [**3c^••^**]^2+^ indicated relatively even spin distributions over the macrocyclic subunits (Figures 10 and 11). For the cation radical, however, there were significant differences in the spin density within each oxacorrole subunit, with its considerably lower concentration at pyrrole carbons C12, C13, and C17 (Figure 10C). The calculations indicated a small contribution of the nickel to the singly occupied molecular orbital (SOMO) as well as a small amount of the spin density at the metal center (Figure 10A), in line with the conclusion drawn from the ESR analysis. Thus, the suggestion regarding the first oxidation of **3** being ligand‐centered was also corroborated by theoretical calculations. For the dication in the triplet state, the calculated spin density was also distributed evenly onto the pyridine‐fused bis(oxacorrole) skeleton (Figure 11C). It was reflected by the spatial distribution of amplitudes of both frontier spin‐orbitals responsible for the presence of the net spin density, i.e., α‐SOMO and α‐SOMO‐1 (Figure 11A). As in the monocation radical, the contribution of nickel to these frontier orbitals was minor, and the net spin density on the metal site was insignificant. Importantly, the *meso*‐aryls, as well as the aryl at C_γ_ of the bridging pyridine, had no contribution to the SOMO orbitals, neither in [**3c^•^**]^+^ nor in [**3c^••^**]^2+^. Indeed, not only methyl signals of the methoxyls of 3,4,5‐(MeO)_3_Ph or *ortho‐* and *para‐*methyl protons but also the *meta*‐H of *meso‐*mesityls and *ortho*‐H of the trimethoxyphenyl gave rise to reasonably sharp signals in the ^1^H NMR spectra of the doubly oxidized product of **3c**, even at 300 K (Figures 7E and S77 in Supporting Information).

**Figure 12:**
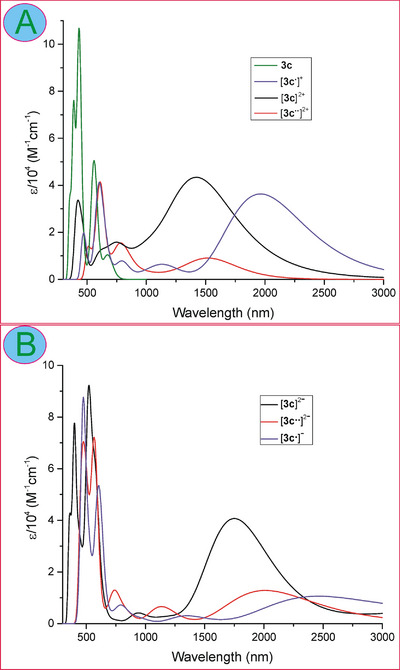
TD DFT calculated UV/Vis/NIR absorption spectra for **3c** (green trace) and its oxidized (A) and reduced (B) forms.

The time‐dependent DFT calculations for the DFT‐optimized structures of **3c** and its oxidized and reduced forms allowed analyses of the electronic transitions and reproduction of the absorption spectra of these species. The calculated spectral data, including transition energies, wavelengths, oscillator strengths, and assignments, were collected in Tables S2–S12 of the Supporting Information. In Figure 12, the calculated spectra (transitions were convoluted with a Gaussian line shape of 2000 nm half‐width) were presented for the neutral **3c**, monocation [**3c^•^**]^+^, and for dication in both singlet and triplet states as well as for anion‐radical and dianion in both triplet and singlet states. The calculations reproduced well the observed tendencies of strong bathochromic shifts of the lowest‐energy transition upon oxidation, i.e., from ≈700 nm in the neutral dimer to ≈2000 nm in monocation and ≈1400 nm for dication. These bands were ascribed as mostly HOMO→LUMO transitions or β‐HOMO→β‐SUMO for the radical and diradical forms. The high intensities of these bands indicated the allowed character of these transitions, although a significant difference in the oscillator strengths between dicationic forms, resulted in about five times stronger absorbance calculated for the singlet than for the triplet states at the lowest‐energy bands. A similar difference was observed for reduced forms (Figure 12B) with about 4‐times stronger absorbance estimated for the diamagnetic dianion [**3c**]^2‐^ than for the diradical [**3c^••^**]^2‐^. Also, for the two‐electron oxidized pyridine‐fused bis(10‐arylazacorroles) the calculated spectra for the singlet state indicate an order of magnitude higher values of the oscillator strength than the triplet‐state dications.^[^ ^35^ ^]^

## 3 Conclusion

We have shown that readily obtainable antiaromatic norcorrolatonickel(II) can be a starting point for the synthetic path leading to the pyridine‐fused bis[10‐oxacorrolatonickel(II)] owing to its selective reactivity in the nitration reaction. The key synthetic steps of the pyridine annulation can be performed with the aromatic aldehydes of both electron‐donating and electron‐withdrawing substituents. The macrocyclic subunits in the dimeric products, though noncoplanar, do not suffer distortions caused by the steric overcrowding introduced by the *meso*‐ or C_γ_‐substitutions. The aromaticity of the dimers is evident from both experimental NMR data and theoretical considerations. However, in the neutral species, the diatropic current seems to be confined separately to each subunit. Dimerization enhances the electron‐donating properties of the oxacorrole rings, considerably lowering their oxidation potentials, while reductions are somewhat anodically shifted with respect to the monomer, which results in a narrower electrochemical HOMO‐LUMO gap. Despite the symmetric structure of the dimer, the number of oxidation and reduction events is doubled relative to the monomer, indicating strong interactions between the subunits. Also, the considerably different electronic spectra of the neutral, cationic, and dicationic forms from those of the monomeric oxacorrole prove the strong electronic interaction within the dimer. ESR and NMR experiments, as well as DFT calculations, indicate spin pairing in the two‐electron oxidized pyridine‐fused bis[10‐oxacorrolatonickel(II)], which, by necessity, requires delocalization of the electron density over the whole system. However, there is no evidence for the aromatic or antiaromatic properties of the dication in the singlet state. The facile and reversible oxidation, unusual spectroscopic properties of the oxidized species, relative stability of the oxidized forms, as well as the possibility of further modifications of dimers, constitute attractive features of these systems of potential application in electronic materials.

## Supporting Information

Supporting Information is available from the Wiley Online Library or from the author.

## Conflict of Interests

The authors declare no conflict of interest.

## Supporting information



Supporting Information

## Data Availability

The data that support the findings of this study are available in the supplementary material of this article.
