# Supplementary material for: Bis(10‐oxacorrole) with Fused Pyridine as a Linker: Synthesis, Structure, and Interaction between the Subunits in the Neutral, Mono‐, and Dicationic States
Source: Chemistry. 2025 Apr 27;31(31):e202501085. doi: 10.1002/chem.202501085 (PMC12133628; doi:10.1002/chem.202501085)
Supplement: Supplementary file 1 — Supporting Information [file CHEM-31-e202501085-s001.pdf]

Table of Contents

General methods and instrumentation S4
Computational methods S4
Syntheses and characterization S6
Synthesis of the precursors S6
Synthesis of 3-NO_2-oxacorrole 2-NO_2 S6
Synthesis of 2-NH_2 S7
General procedure of synthesis of pyridine-fused bis(oxacorrolatonickel(II)) S8
References S67
Figure S1. ^{1}H NMR (500 MHz, $CDCl_3$, 300 K) of **2-NO_2**.
S11
Figure S2. ^{13}C NMR (125 MHz, $CDCl_3$, 300 K) of **2-NO_2**. S11
Figure S3. ^{1}H-^{1}H COSY (600 MHz, $CDCl_3$, 300 K) of **2-NO_2**. S12
Figure S4. ^{1}H,^{13}C HSQC spectrum (600/150 MHz, $CDCl_3$, 300 K) of **2-NO_2**. S12
Figure S5. ^{1}H,^{13}C HMBC spectrum (600/150 MHz, $CDCl_3$, 300 K) of **2-NO_2**. S13
Figure S6. ^{1}H NMR (400 MHz, $CDCl_3$, 300 K) of **2-NH_2**. S13
Figure S7. ^{13}C NMR (100 MHz, $CDCl_3$, 300 K) of **2-NH_2**. S14
Figure S8. ^{1}H-^{1}H COSY (600 MHz, $CDCl_3$, 300 K) of **2-NH_2**. S14
Figure S9. ^{1}H,^{13}C HSQC spectrum (600/150 MHz, $CDCl_3$, 300 K) of **2-NH_2**. S15
Figure S10. ^{1}H,^{13}C HMBC spectrum (600/150 MHz, $CDCl_3$, 300 K) of **2-NH_2**. S15
Figure S11. ^{1}H NMR spectrum (600 MHz, $CDCl_3$, 300 K) of **3a**. S16
Figure S12. ^{13}C NMR spectrum (150 MHz, $CDCl_3$, 300 K) of **3a**. S16
Figure S13. ^{1}H,^{1}H COSY spectrum (600 MHz, $CDCl_3$, 300 K) of **3a**. S17
Figure S14. ^{1}H,^{1}H ROESY spectrum (600 MHz, $CDCl_3$, 300 K) of **3a**. S17
Figure S15. ^{1}H,^{13}C HSQC spectrum (600/150 MHz, $CDCl_3$, 300 K) of **3a**. S18
Figure S16. ^{1}H,^{13}C HMBC spectrum (600/150 MHz, $CDCl_3$, 300 K) of **3a**. S18
Figure S17. ^{1}H NMR spectrum (600 MHz, $CDCl_3$, 300 K) of **3b**. S19
Figure S18. ^{13}C NMR spectrum (150 MHz, $CDCl_3$, 300 K) of **3b**. S19
Figure S19. ^{1}H,^{1}H COSY spectrum (600 MHz, $CDCl_3$, 300 K) of **3b**. S20
Figure S20. ^{1}H,^{1}H ROESY spectrum (600 MHz, $CDCl_3$, 300 K) of **3b**. S20
Figure S21. ^{1}H,^{13}C HSQC spectrum (600/150 MHz, $CDCl_3$, 300 K) of **3b**. S21
Figure S22. ^{1}H,^{13}C HMBC spectrum (600/150 MHz, $CDCl_3$, 300 K) of **3b**. S21
Figure S23. ^{1}H NMR spectrum (600 MHz, $CDCl_3$, 300 K) of **3c**. S22
Figure S24. ^{13}C NMR spectrum (150 MHz, $CDCl_3$, 300 K) of **3c**. S22
Figure S25. ^{1}H,^{1}H COSY spectrum (600 MHz, $CDCl_3$, 300 K) of **3c**. S23
Figure S26. ^{1}H,^{1}H ROESY spectrum (600 MHz, $CDCl_3$, 300 K) of **3c**. S23
Figure S27. ^{1}H,^{13}C HSQC spectrum (600/150 MHz, $CDCl_3$, 300 K) of **3c**. S24
Figure S28. ^{1}H,^{13}C HMBC spectrum (600/150 MHz, $CDCl_3$, 300 K) of **3c**. S24
Figure S29. ^{1}H NMR spectrum (600 MHz, $CDCl_3$, 300 K) of **3d**. S25
Figure S30. ^{13}C NMR spectrum (150 MHz, $CDCl_3$, 300 K) of **3d**. S25
Figure S31. ^{1}H,^{1}H COSY spectrum (600 MHz, $CDCl_3$, 300 K) of **3d**. S26
Figure S32. ^{1}H,^{1}H ROESY spectrum (600 MHz, $CDCl_3$, 300 K) of **3d**. S26
Figure S33. ^{1}H,^{13}C HSQC spectrum (600/150 MHz, $CDCl_3$, 300 K) of **3d**. S27
Figure S34. ^{1}H,^{13}C HMBC spectrum (600/150 MHz, $CDCl_3$, 300 K) of **3d**. S27
Figure S35. ^{1}H NMR spectrum (600 MHz, $CDCl_3$, 300 K) of **3e**. S28
Figure S36. ^{13}C NMR spectrum (150 MHz, $CDCl_3$, 300 K) of **3e**. S28
Figure S37. ^{1}H,^{1}H COSY spectrum (600 MHz, $CDCl_3$, 300 K) of **3e**. S29
Figure S38. ^{1}H,^{1}H ROESY spectrum (600 MHz, $CDCl_3$, 300 K) of **3e**. S29
Figure S39. ^{1}H,^{13}C HSQC spectrum (600/150 MHz, $CDCl_3$, 300 K) of **3e**. S30

Figure S40. $^{1}H,^{13}C$ HMBC spectrum (600/150 MHz, CDCl3, 300 K) of **3e**. S30
Figure S41. API-HRMS spectrum of **2-NO$_2$**. S31
Figure S42. API-HRMS spectrum of **2-NH$_2$**. S31
Figure S43. API-HRMS spectrum of **3a**. S32
Figure S44. ESI-HRMS spectrum of **3a** (experimental: red, upper trace; simulated: black, bottom trace). S32
Figure S45. API-HRMS spectrum of **3b**. S33
Figure S46. ESI-HRMS spectrum of **3b** (experimental: red, upper trace; simulated: black, bottom trace). S33
Figure S47. API-HRMS spectrum of **3c**. S34
Figure S48. ESI-HRMS spectrum of **3c** (experimental: red, upper trace; simulated: black, bottom trace). S34
Figure S49. API-HRMS spectrum of **3d**. S35
Figure S50. ESI-HRMS spectrum of **3d** (experimental: red, upper trace; simulated: black, bottom trace). S35
Figure S51. API-HRMS spectrum of **3e**. S36
Figure S52. ESI-HRMS spectrum of **3e** (experimental: red, upper trace; simulated: black, bottom trace). S36
Figure S53. UV-Vis spectrum of **2-NO$_2$** in dichloromethane. S37
Figure S54. UV-Vis spectrum of **2-NH$_2$** in dichloromethane. S37
Figure S55. UV-Vis spectrum of **3a** in dichloromethane. S38
Figure S56. UV-Vis spectrum of **3b** in dichloromethane. S38
Figure S57. UV-Vis spectrum of **3c** in dichloromethane. S39
Figure S58. UV-Vis spectrum of **3d** in dichloromethane. S39
Figure S59. UV-Vis spectrum of **3e** in dichloromethane. S40
Figure S60. Differential pulse (DP) and cyclic (CV) voltammograms recorded for **2-NO$_2$** in DCM with $[Bu_4N]PF_6$ as supporting electrolyte. S40
Figure S61. Differential pulse (DP) and cyclic (CV) voltammograms recorded for **2-NH$_2$** in DCM with $[Bu_4N]PF_6$ as supporting electrolyte. S41
Figure S62. Differential pulse (DP) and cyclic (CV) voltammograms recorded for **3a** in DCM with $[Bu_4N]PF_6$ as supporting electrolyte. S41
Figure S63. Differential pulse (DP) and cyclic (CV) voltammograms recorded for **3b** in DCM with $[Bu_4N]PF_6$ as supporting electrolyte. S42
Figure S64. Differential pulse (DP) and cyclic (CV) voltammograms recorded for **3c** in DCM with $[Bu_4N]PF_6$ as supporting electrolyte. S42
Figure S65. Differential pulse (DP) and cyclic (CV) voltammograms recorded for **3d** in DCM with $[Bu_4N]PF_6$ as supporting electrolyte. S43
Figure S66. Differential pulse (DP) and cyclic (CV) voltammograms recorded for **3e** in DCM with $[Bu_4N]PF_6$ as supporting electrolyte. S43
Figure S67. UV-Vis-NIR spectra recorded upon electrochemical oxidation of **2-NO$_2$** (0.1 M $[Bu_4N]PF_6$, DCM). S44
Figure S68. UV-Vis-NIR spectra recorded upon electrochemical oxidation of **3c** (0.1 M $[Bu_4N]PF_6$, DCM). Upper panel presents spectral changes during anodic scan and the bottom panel shows spectral changes during cathodic scan. S45
Figure S69. UV-Vis-NIR spectra recorded upon addition of tris(4-bromophenyl)ammoniumyl hexachloroantimonate (BAHA) to the DCM solution of **3c** with the amount of the added oxidant specified in the legend. S46
Figure S70. Two views of the ORTEP representations (ellipsoids set on the 50% probability level) of the asymmetric unit of the **2-NO$_2$** crystal structure. S48
Figure S71. Two views of the ORTEP representations (ellipsoids set on the 50% probability level) of the asymmetric unit of the **3a** crystal structure. S49

Figure S72. Two views of the ORTEP representations (ellipsoids set on the 50% probability level) of the asymmetric unit of the **3c** crystal structure. S50
Figure S73. Top: solid state ESR spectra of [**3c**]$^{2+}$ at specified temperatures. Bottom: temperature dependence of the product of temperature and doubly integrated intensity of the ESR signals from the spectra taken upon the variable-temperature ESR experiment for the solid sample of [3c]$^{2+}$. The black squares represent experimental data, and the red line is the best fit curve calculated on the basis of the data fitting to the Bleaney-Bowers equation with the exchange integral J = –0.46 kcal/mol. S51
Figure S74. GIAO calculated NICS(x) values (x = –1, 0, 1) and calculated pyrrole proton chemical shifts (green numbers; in ppm) for **3c**. The NICS(x) were estimated x Å over a mean plane of the macrocycle in the midpoint of each ring specified with the blue letter. S52
Figure S75. GIAO calculated NICS(x) values (x = –1, 0, 1) and calculated pyrrole proton chemical shifts (green numbers; in ppm) for [**3c**]$^{2+}$. The NICS(x) were estimated x Å over a mean plane of the macrocycle in the midpoint of each ring specified with the blue letter. S52
Figure S76. GIAO calculated NICS(x) values (x = –1, 0, 1) and calculated pyrrole proton chemical shifts (green numbers; in ppm) for [**3c**]$^{2-}$. The NICS(x) were estimated x Å over a mean plane of the macrocycle in the midpoint of each ring specified with the blue letter. S53
Figure S77. Variable temperature ^{1}H NMR (600 MHz) experiment for [**3c**]$^{2+}$ in CD_2Cl_2.
S52
Table S1. Crystal data for **2-NO$_2$**, **3a** and **3c**.
S47
Table S2. TD DFT calculated electronic transitions for **3c** (in vacuo). S54
Table S3. TD DFT calculated electronic transitions for **3c** (PCM, dichloromethane) S55
Table S4. TD DFT calculated electronic transitions for [**3c**$^{\bullet}$]$^{+}$ (in vacuo). S56
Table S5. TD DFT calculated electronic transitions for [**3c**$^{\bullet}$]$^{+}$ (DCM). S57
Table S6. TD DFT calculated electronic transitions for triplet [**3c**$^{\bullet\bullet}$]$^{2+}$ (in vacuo) S58
Table S7. TD DFT calculated electronic transitions for triplet [**3c**$^{\bullet\bullet}$]$^{2+}$ (DCM). S59
Table S8. TD DFT calculated electronic transitions for singlet [**3c**]$^{2+}$ (*in vacuo*). S60
Table S9. TD DFT calculated electronic transitions for singlet [**3c**]$^{2+}$ (DCM). S61
Table S10. TD DFT calculated electronic transitions for [**3c**$^{\bullet}$]$^{-}$ (*in vacuo*) S62
Table S11. TD DFT calculated electronic transitions for triplet [**3c**$^{\bullet\bullet}$]$^{2-}$ (*in vacuo*). S63
Table S12. TD DFT calculated electronic transitions for triplet [**3c**]$^{2-}$ (in vacuo). S65
Table S13. Computational details for the optimized structures of compounds. S66

General methods and instrumentation

All reagents and solvents were obtained from commercial suppliers and used without further purification unless specified otherwise. Analytical TLCs were performed with silica gel 60 F254 plates. Column chromatography was carried out by using silica gel 60 (200–300 mesh ASTM).

NMR spectra were recorded on Bruker 400, 500 or 600 MHz spectrometers (operating at ^{1}H frequency of 400, 500 or 600 MHz and 100, 125 and 150 MHz for ^{13}C, respectively). ^{1}H NMR spectra were referenced to the residual solvent signal ($CDCl_3$, 7.26 ppm). ^{13}C NMR spectra were recorded with ^{1}H broadband decoupling and referenced to solvent signals ($^{13}CDCl_3$, 77.2 ppm). Standard pulse programs from the Bruker library were used for homo- and heteronuclear 2D experiments.

High resolution mass spectra (HRMS) were recorded using Finnigan LCQ Advantage MAX and Bruker qTOF Compact mass spectrometers.

UV-Vis-NIR spectra were recorded on Varian Cary 60 and Jasco V-770 spectrophotometers. Electrochemical measurements were performed by means of Autolab (Metrohm) potentiostat/galvanostat system for dichloromethane solutions with a glassy carbon, a platinum wire, and Ag/Ag^{+} as the working, auxiliary, and reference electrodes, respectively. Tetrabutylammonium hexafluorophosphate was used as a supporting electrolyte. The potentials were referenced with the ferrocene/ferrocenium couple used as an internal standard. Spectroelectrochemical measurements were conducted in dichloromethane solution with $[Bu_4N]PF_6$ supporting electrolyte by means of optically transparent thin-layer electrochemical (OTTLE) cell consisting of platinum gauze as working and auxiliary electrodes and a silver wire as a pseudoreference electrode sealed between transparent fluorite windows.

X-ray diffraction data are summarized in Table S1. Data reduction and analysis were carried out with the CrysAlis programs. Structures were solved using the SHELXT program[1] and refined by the full-matrix least-squares method on all F^2 data by using the SHELXL-2018/3 incorporated in the Olex program.[2] All hydrogen atoms, including those located in the difference density map, were placed in calculated positions and refined as the riding model.

Computational methods

Density functional theory (DFT) calculations were performed by using the Gaussian 16 program.[3] DFT optimizations were carried out using ωB97xD functional[4] and 6-31G(d,p) basis set. Dications were additionally also optimized using CAM-B3LYP functional[5], including GD3BJ dispersion correction.[6] Optimizations were performed in vacuo. Energy minima were obtained as no imaginary frequencies were found. TD-DFT calculations were done using B3LYP functional[7,8] and the same basis set. All were solved for 60 states and including implicit solvent (PCM; dichloromethane). The electronic transitions and UV-vis spectra were analyzed by means of the GaussSum program[9]. The transitions were convoluted by Gaussian curves with 2000 cm^{-1} half line width. Population analysis has been done using NBO.[10] NMR chemical shifts were calculated using the GIAO method [B3LYP/6-31G(d,p)] with TMS shieldings as a reference for NMR. NICS(-1,0,1) parameters were calculated at centers of selected cycles and 1 Å below and above their plane. The computational details are collected in Table S13.

Syntheses and characterization

Synthesis of the precursors Starting 3-nitro-5,14-dimesitylnorcorrolatonickel(II) complex **1-NO_2** was obtained from 5,14-dimesitylnorcorrolatonickel(II) complex **1** as described previously.[11]

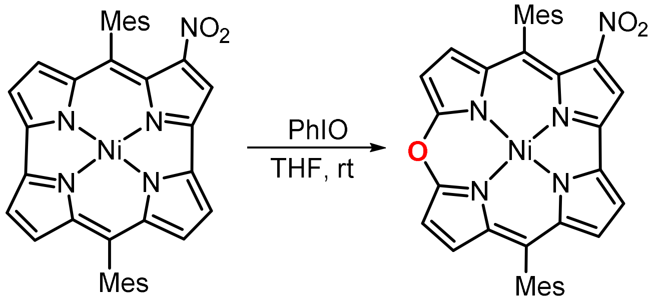

1-NO_2　　**2-NO_2**

Synthesis of 3-NO_2-oxacorrole 2-NO_2

A solution of 3-NO_2-norcorrole **1-NO_2** (61.5 mg, 0.1 mmol) and PhIO (55 mg, 0.25 mmol) in 15 ml THF was stirred at room temperature for 3 h; then the solvent was evaporated under vacuum. The residue was subjected to silica-gel column chromatography with petroleum ether/ dichloromethane (v/v = 2.5:1) as eluent. All the red fractions were collected together and concentrated. The product 3-NO_2-oxacorrole **2-NO_2** was isolated by precipitation and filtration from CH_3OH. Yield: 39 mg (61%).

2-NO_2: ^1H NMR (600 MHz, $CDCl_3$, 300 K) δ: 8.30 (s, 1H, pyrrH), 7.81 (d, 1H, ^3J = 4.86 Hz, pyrrH), 7.76 (m, 2H, pyrrH), 7.58 (d, 1H, ^3J = 4.86 Hz, pyrrH), 7.53 (d, 1H, ^3J = 4.30 Hz, pyrrH), 7.48 (d, 1H, ^3J = 4.65 Hz, pyrrH), 7.11 (s, 2H, ArH), 7.06 (s, 2H, ArH), 2.49 (s, 3H, -CH_3), 2.47 (s, 3H, -CH_3), 1.98 (s, 6H, -CH_3), 1.95 (s, 6H, -CH_3). ^{13}C NMR (150 MHz, $CDCl_3$, 300 K) δ: 157.1, 153.7, 148.2, 144.6, 140.4, 138.4, 138.2, 137.9, 137.8 (pyrrC), 137.6, 136.8, 134.4, 134.0, 133.4 (pyrrC), 133.4, 133.2, 133.0, 129.8, 128.1, 128.0, 127.6 (pyrrC), 125.4, 116.9 (pyrrC), 114.0 (pyrrC), 114.0, 109.2 (pyrrC), 21.6 (-CH_3), 21.5 (-CH_3), 21.0 (-CH_3), 20.8 (-CH_3). UV-vis (CH_2Cl_2) λ_{max}/nm (logε): 401 (4.80), 529 (3.97), 569 (4.29), 626 (3.74), 673 (3.78). API-HRMS calc. for $C_{36}H_{30}N_5NiO_3^+$ $[M+H]^+$: 638.1697, Found: 638.1693.

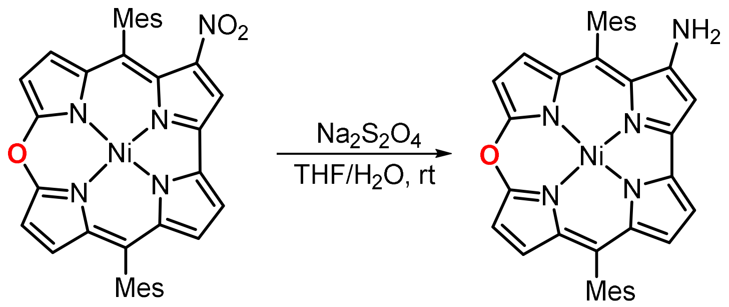

2-NO$_2$ **2-NH$_2$**

Synthesis of 2-NH$_2$

A solution of 3-NO$_2$-oxacorrole **2-NO$_2$** (10 mg, 0.016 mmol), $Na_2S_2O_4$ (33 mg, 0.192 mmol) in a mixture of THF (2 mL) and water (1 mL) was stirred at room temperature for 20 min; then the solvent was evaporated under vacuum. The residue was subjected to alumina column chromatography with dichloromethane as eluent. All the brown fractions were collected together and concentrated. The product 3-NH$_2$-oxacorrole **2-NH$_2$** was isolated by precipitation and filtration from CH_3OH. Yield: 6.7 mg (70%).

2-NH$_2$: ^{1}H NMR (600 MHz, $CDCl_3$, 300 K) δ: 7.81 (d, 1H, ^3J = 4.56 Hz, pyrrH), 7.75 (d, 1H, ^3J = 4.35 Hz, pyrrH), 7.54 (d, 2H, ^3J = 4.30 Hz, pyrrH), 7.52 (d, 2H, ^3J = 4.56 Hz, pyrrH), 7.47 (d, 2H, ^3J = 4.35 Hz, pyrrH), 7.40 (d, 2H, ^3J = 4.30 Hz, pyrrH), 7.17 (s, 2H, ArH), 7.10 (s, 2H, ArH), 6.91 (s, 1H, pyrrH), 4.08 (s, 2H, -NH$_2$), 2.50 (s, 6H, -CH$_3$), 2.02 (s, 6H, -CH$_3$), 1.98 (s, 6H, - CH$_3$). ^{13}C NMR (150 MHz, $CDCl_3$, 300 K) δ: 155.0, 151.9, 150.5, 150.2, 145.5, 138.6, 138.2, 137.9, 137.9, 134.0, 133.6 (pyrrC), 133.4, 133.2, 132.9, 130.1, 130.0, 129.0, 127.9, 127.8 (pyrrC), 127.5, 125.1 (pyrrC), 122.8, 116.8 (pyrrC), 110.5 (pyrrC), 104.9 (pyrrC), 96.7 (pyrrC), 21.5 (-CH$_3$), 21.4 (-CH$_3$), 20.8 (-CH$_3$), 20.5 (-CH$_3$). UV-vis (CH_2Cl_2) λ_{max}/nm (logε): 392 (4.83), 443 (4.29), 466 (4.27), 491 (4.44), 569 (3.78), 663 (3.62), 714 (3.68). API-HRMS calc. for $C_{36}H_{31}N_5NiO^+$ [M]$^+$: 607.1877, Found: 607.1878.

General procedure of synthesis of pyridine-fused bis(oxacorrolatonickel(II))

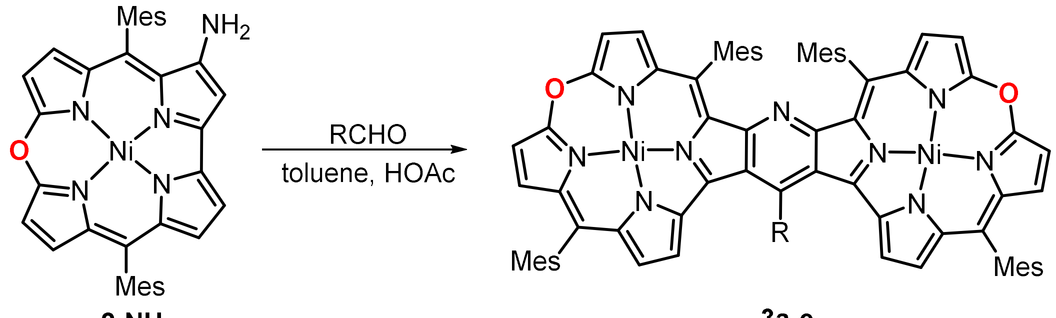

A solution of 3-NH_2-oxacorrole **2-NH₂** (20 mg, 0.033 mmol), and aromatic aldehyde (0.132 mmol, 4 eq.) in 4 mL toluene in the presence of HOAc (150 ul) was refluxed at 110℃ for 30 min, then chloranil (8 mg) was added and the reaction mixture was further stirred for 10 min. Then, the solvent was evaporated under vacuum. The residue was chromatographed on a silica gel column with petroleum ether /dichloromethane (v/v = 5:1) as eluent to afford the desired product **3a-e**.

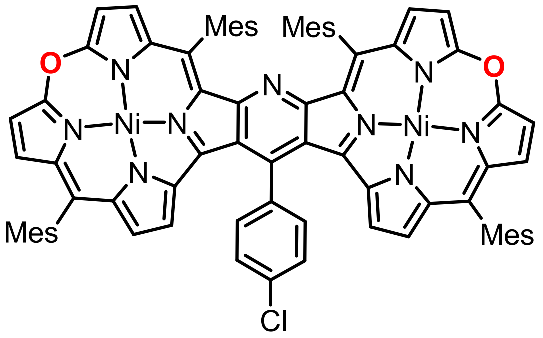

3a: 11 mg, yield 51%. ^{1}H NMR (600 MHz, $CDCl_3$, 300 K) δ: 8.10 (d, 2H, ^{3}J = 7.95, ArH), 7.96 (d, 2H, ^{3}J = 7.95, ArH), 7.93 (d, 2H, ^{3}J = 4.44 Hz, pyrrH) 7.67 (d, 2H, ^{3}J = 4.44, pyrrH), 7.50 (d, 2H, ^{3}J = 4.40, pyrrH), 7.38 (d, 2H, ^{3}J = 4.40, pyrrH), 7.24 (d, 2H, ^{3}J = 4.40, pyrrH), 7.09 (s, 4H, ArH), 7.01 (s, 4H, ArH), 5.80 (d, 2H, 3J = 4.40, pyrrH) 2.74 (s, 6H, -CH_3), 2.49 (s, 6H, - CH_3), 1.93 (s, 12H, -CH_3), 1.77 (s, 12H, -CH_3). ^{13}C NMR (150 MHz, $CDCl_3$, 300 K) δ: 154.5, 154.2, 150.5, 144.8, 142.9, 138.5, 138.1, 138.0, 137.8, 136.3, 135.9, 134.7, 133.8 (pyrrC), 133.8, 132.6, 132.3, 130.8, 130.5 (pyrrC), 130.3, 129.9, 128.0, 127.9, 127.2, 125.4 (pyrrC), 122.2, 119.2 (pyrrC), 110.9 (pyrrC), 105.9 (pyrrC), 22.2 (-CH_3), 21.5 (-CH_3), 20.9 (-CH_3), 20.7 (-CH_3). UV-vis (CH_2Cl_2) λ_{max}/nm (logε): 331 (4.56), 383 (4.79), 456 (4.96), 560 (4.48), 608 (4.75), 682 (4.29), 752 (4.37). API-HRMS calc. for $C_{79}H_{61}ClN_9Ni_2O_2^+$ $[M+H]^+$: 1318.3338, found: 1318.3337. ESI-HRMS calc. for $C_{79}H_{60}ClN_9Ni_2O_2^+$ $[M]^+$: 1317.3260, found: 1317.3554.

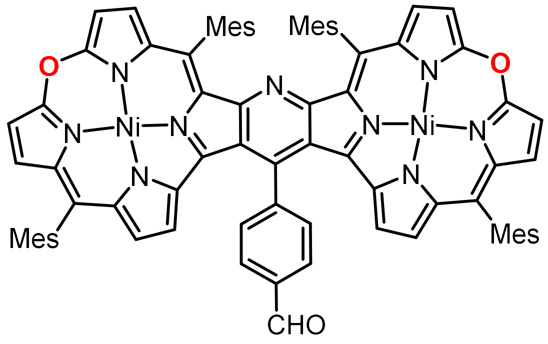

3b: 8 mg, yield 37%. ^{1}H NMR (600 MHz, $CDCl_3$, 300 K) δ: 10.43 (s, 1H, -CHO), 8.49 (d, 2H, ^{3}J = 7.87 Hz, ArH), 8.37 (d, 2H, ^{3}J = 7.87 Hz, ArH), 7.93 (d, 2H, ^{3}J = 4.52 Hz, pyrrH), 7.67 (d, 2H, ^{3}J = 4.52 Hz, pyrrH), 7.50 (d, 2H, pyrrH), 7.27 (d, 2H, ^{3}J = 4.40Hz, pyrrH), 7.25 (d, 2H, ^{3}J = 4.50 Hz, pyrrH), 7.07 (s, 4H, ArH), 7.02 (s, 4H, ArH), 5.60 (d, 2H, ^{3}J = 4.30 Hz, pyrrH), 2.74 (s, 6H, $-CH_3$), 2.37 (s, 6H, $-CH_3$), 1.91 (s, 12H, $-CH_3$), 1.77 (s, 12H, $-CH_3$). ^{13}C NMR (150 MHz, $CDCl_3$, 300 K) δ: 191.9 (-CHO), 169.8, 154.5, 154.1, 150.6, 146.2, 144.6, 142.5, 141.0, 138.1, 137.8, 137.5, 136.0, 134.0, 133.8 (pyrrC), 132.3, 131.7, 130.7 (pyrrC), 130.6, 130.4, 129.9, 128.0, 127.9, 127.1, 125.4 (pyrrC), 124.7, 124.2, 121.5, 119.3, 118.8 (pyrrC), 111.5 (pyrrC), 106.0 (pyrrC), 22.2 ($-CH_3$),21.4($-CH_3$), 20.9 ($-CH_3$), 20.7 ($-CH_3$). UV-vis (CH_2Cl_2) λ_{max}/nm (logε): 331 (4.50), 382 (4.76), 457 (4.92), 559 (4.44), 608 (4.71), 680 (4.26), 752 (4.36). API-HRMS calc. for $C_{80}H_{62}N_9Ni_2O_3^+$ $[M+H]^+$: 1312.3677, Found: 1312.3672. ESI-HRMS calc. for calc. for $C_{80}H_{61}N_9Ni_2O_3^+$ $[M]^+$: 1311.3599, found: 1311.3599.

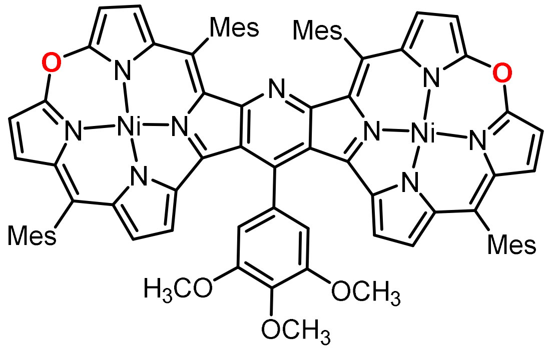

3c: 9 mg, yield 40%. ^{1}H NMR (500 MHz, $CDCl_3$, 300 K) δ: 7.92 (d, 2H, ^{3}J = 4.59 Hz, pyrrH), 7.66 (d, 2H, ^{3}J = 4.62 Hz, pyrrH), 7.48 (d, 2H, ^{3}J = 4.35 Hz, pyrrH), , 7.36 (d, 2H, ^{3}J = 4.38 Hz, pyrrH), 7.35 (s, 2H, ArH), 7.23 (d, 2H, ^{3}J = 4.35 Hz, pyrrH), 7.11 (s, 4H, ArH), 7.01 (s, 4H, ArH), 6.09 (d, 2H, ^{3}J = 4.38 Hz, pyrrH), 4.21(s, 3H, - OCH_3), 3.81 (s, 6H, $-OCH_3$), 2.74 (s, 6H, $-CH_3$), 2.50 (s, 6H, $-CH_3$), 1.94 (s, 12H, $-CH_3$), 1.77 (s, 12H, $-CH_3$). ^{13}C NMR (125 MHz, $CDCl_3$, 300 K) δ: 155.1, 154.2, 150.3, 144.9, 143.0, 140.2, 138.0, 137.9, 137.6, 136.6, 135.7, 134.7, 134.6, 133.7, 133.6 (pyrrC), 132.0, 130.3 (pyrrC), 130.2, 130.0, 129.7, 127.8, 127.7, 127.1, 125.3, 122.1, 119.7 (pyrrC), 110.6 (pyrrC), 108.2 (pyrrC), 105.6 (pyrrC), 61.8 ($-OCH_3$), 56.7 ($-OCH_3$), 22.0 ($-CH_3$), 21.3 ($-CH_3$), 20.7 ($-CH_3$), 20.5 ($-CH_3$). UV-vis (CH_2Cl_2) λ_{max}/nm (logε): 382 (4.48), 457 (4.65), 560 (4.20), 608 (4.44), 680 (3.96), 751 (4.06). API-HRMS calc. for $C_{82}H_{68}N_9Ni_2O_5^+$ $[M+H]^+$: 1374.4045, Found: 1374.4048. ESI-HRMS calc. for $C_{82}H_{67}N_9Ni_2O_5^+$ $[M]^+$: 1373.3967, Found: 1373.4264.

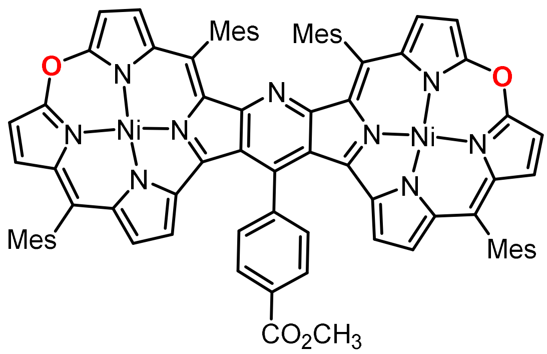

3d: 8 mg, yield 36%. ^{1}H NMR (600 MHz, $CDCl_3$, 300 K) δ: 8.64 (d, 2H, ^3J = 7.84 Hz, ArH), 8.26 (d, 2H, ^3J = 7.84 Hz, ArH), 7.92 (d, 2H, ^3J = 4.49 Hz, pyrrH), 7.66 (d, 2H, ^3J = 4.49 Hz, pyrrH), 7.50 (d, 2H, ^3J = 4.36 Hz, pyrrH), 7.29 (d, 2H, ^3J = 4.37 Hz, pyrrH), 7.25 (d, 2H, ^3J = 4.37 Hz, pyrrH), 7.08 (s, 4H, ArH), 7.02 (s, 4H, ArH), 5.63 (d, 2H, ^3J = 4.37 Hz, pyrrH), 4.12 (s, 6H, $-CO_2CH_3$), 2.74 (s, 6H, $-CH_3$), 2.48 (s, 6H, $-CH_3$), 1.92 (s, 12H, $-CH_3$), 1.77 (s, 12H, $-CH_3$). ^{13}C NMR (150 MHz, $CDCl_3$, 300 K) δ: 166.9 ($-\underline{CO_2}CH_3$), 154.5, 154.2, 150.6, 144.7, 142.8, 138.1, 138.0, 137.8, 135.9, 134.7, 133.8 (pyrrC), 132.3, 131.7, 131.6, 131.4, 130.6 (pyrrC), 130.5, 130.3, 129.9, 127.9, 127.9, 127.2, 125.4 (pyrrC), 121.8, 119.2 (pyrrC), 110.9 (pyrrC), 106.0 (pyrrC), 52.7 ($-CO_2\underline{CH_3}$), 22.2 ($-CH_3$), 21.4 ($-CH_3$), 20.9 ($-CH_3$), 20.7 ($-CH_3$). UV-vis (CH_2Cl_2) λ_{max}/nm (logε): 382 (4.61), 458 (4.77), 560 (4.29), 608 (4.55), 680 (4.09), 752 (4.17). API-HRMS calc. for $C_{81}H_{64}N_9Ni_2O_4^+$ $[M+H]^+$: 1342.3783, Found: 1342.3787. ESI-HRMS calc. for $C_{81}H_{63}N_9Ni_2O_4^+$ $[M]^+$: 1341.3704, Found: 1341.3944.

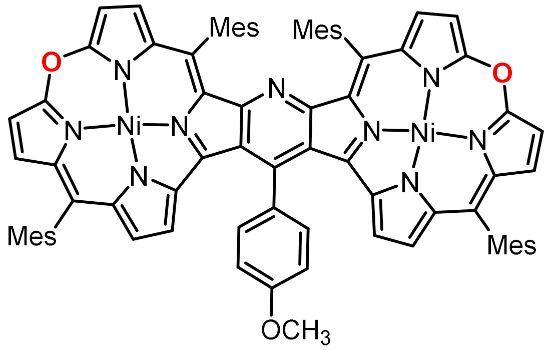

3e: 10 mg, yield 46%. ^{1}H NMR (600 MHz, $CDCl_3$, 300 K) δ: 8.03 (d, 2H, ^3J = 7.68 Hz, ArH), 7.92 (d, 2H, ^3J = 4.46 Hz, pyrrH), 7.66 (d, 2H, ^3J = 4.47 Hz, pyrrH), 7.47 (s, 2H, ^3J = 7.60 Hz, ArH), 7.35 (d, 2H, ^3J = 4.45 Hz, pyrrH), 7.23 (d, 2H, ^3J = 4.38 Hz, pyrrH), 7.09 (s, 4H, ArH), 7.01 (s, 4H, ArH), 5.96 (d, 2H, ^3J = 4.33 Hz, pyrrH), 4.11 (s, 3H, $-OCH_3$), 2.74 (s, 6H, $-CH_3$), 2.49 (s, 6H, $-CH_3$), 1.93 (s, 12H, $-CH_3$), 1.77 (s, 12H, $-CH_3$). ^{13}C NMR (150 MHz, $CDCl_3$, 300 K) δ: 161.5, 154.5, 150.4, 145.0, 143.5, 138.2, 138.0, 137.8, 135.8, 134.8, 133.9, 133.8, 133.7 (pyrrC), 132.3, 132.1, 132.1, 130.5, 130.3 (pyrrC), 130.1, 129.8, 127.9, 127.8, 127.3, 125.3 (pyrrC), 123.0, 119.8 (pyrrC), 116.0, 110.8 (pyrrC), 105.7 (pyrrC), 56.0 ($-OCH_3$), 22.2 ($-CH_3$), 21.5 ($-CH_3$), 20.9 ($-CH_3$), 20.7 ($-CH_3$). UV-vis (CH_2Cl_2) λ_{max}/nm (logε): 383 (4.71), 457 (4.88), 560 (4.41), 607 (4.68), 680 (4.20), 752 (4.29). API-HRMS calc. for $C_{80}H_{64}N_9Ni_2O_3^+$ $[M+H]^+$: 1314.3834, Found: 1314.3839. ESI-HRMS calc. for $C_{80}H_{63}N_9Ni_2O_3^+$ $[M]^+$: 1313.3755, Found: 1313.4151.

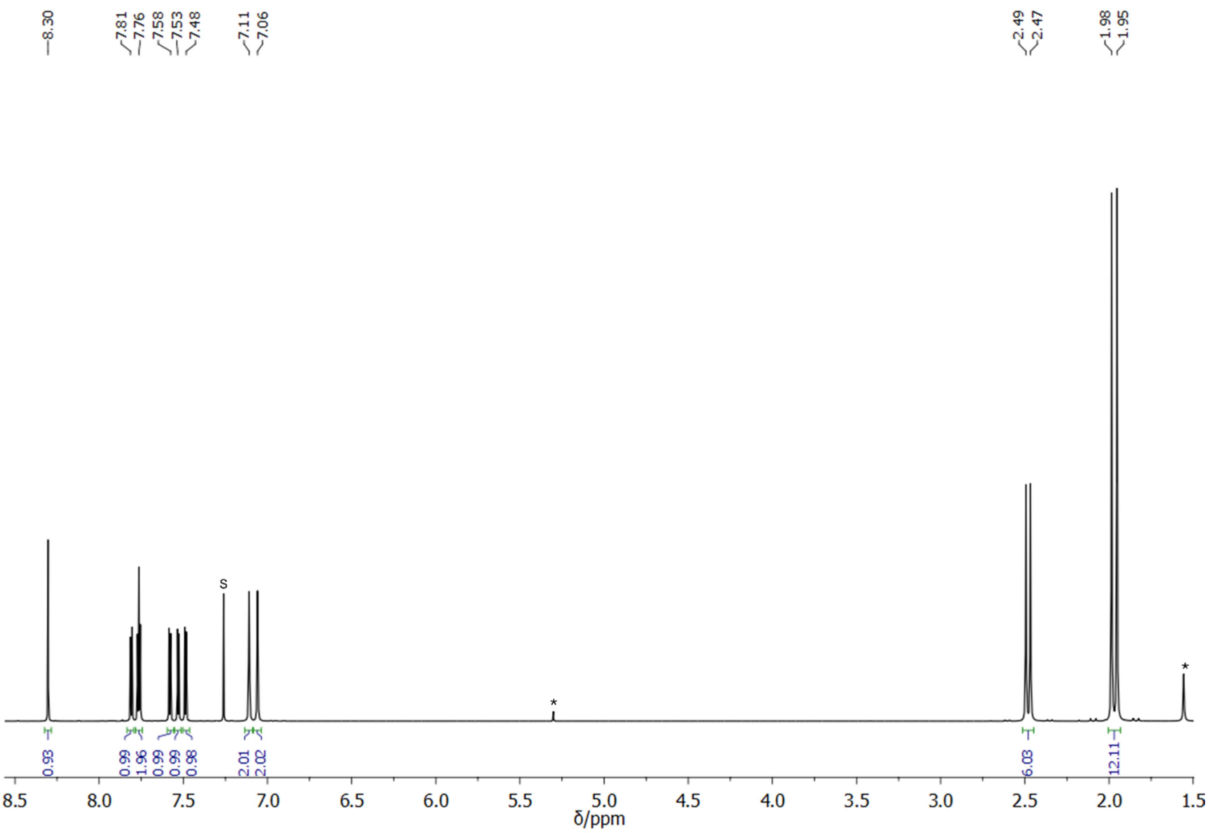

Figure S1. ^{1}H NMR (500 MHz, $CDCl_3$, 300 K) of **2-NO$_2$**.

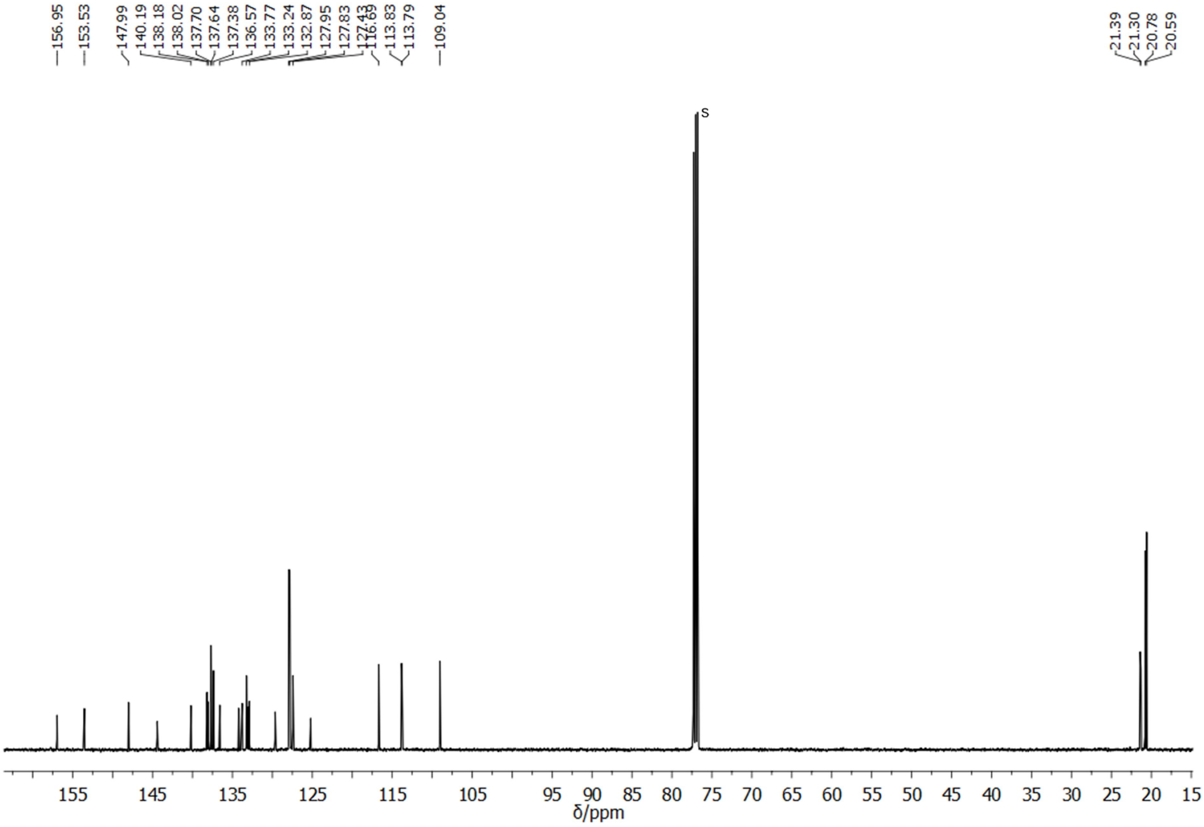

Figure S2. ^{13}C NMR (125 MHz, $CDCl_3$, 300 K) of **2-NO$_2$**.

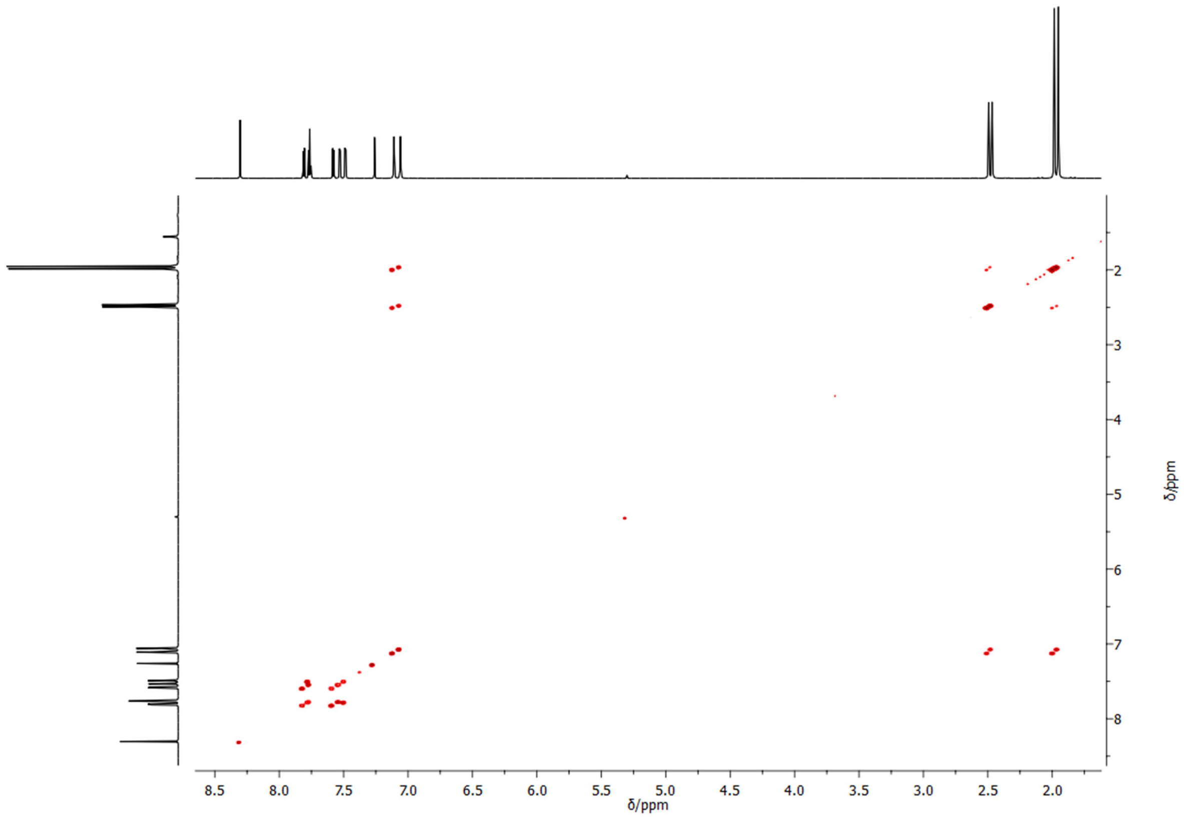

Figure S3. ^1H-^1H COSY (600 MHz, $CDCl_3$, 300 K) of **2-NO$_2$**.

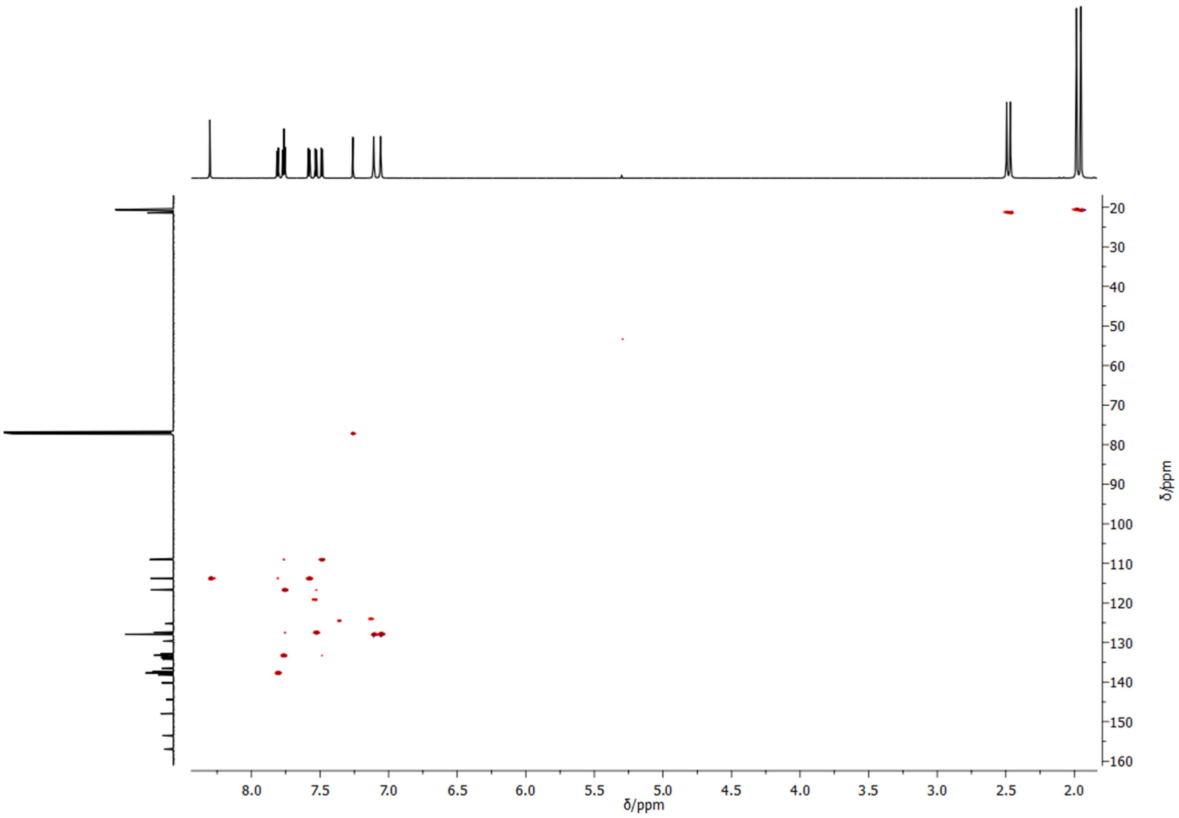

Figure S4. ^1H,^{13}C HSQC spectrum (600/150 MHz, $CDCl_3$, 300 K) of **2-NO$_2$**.

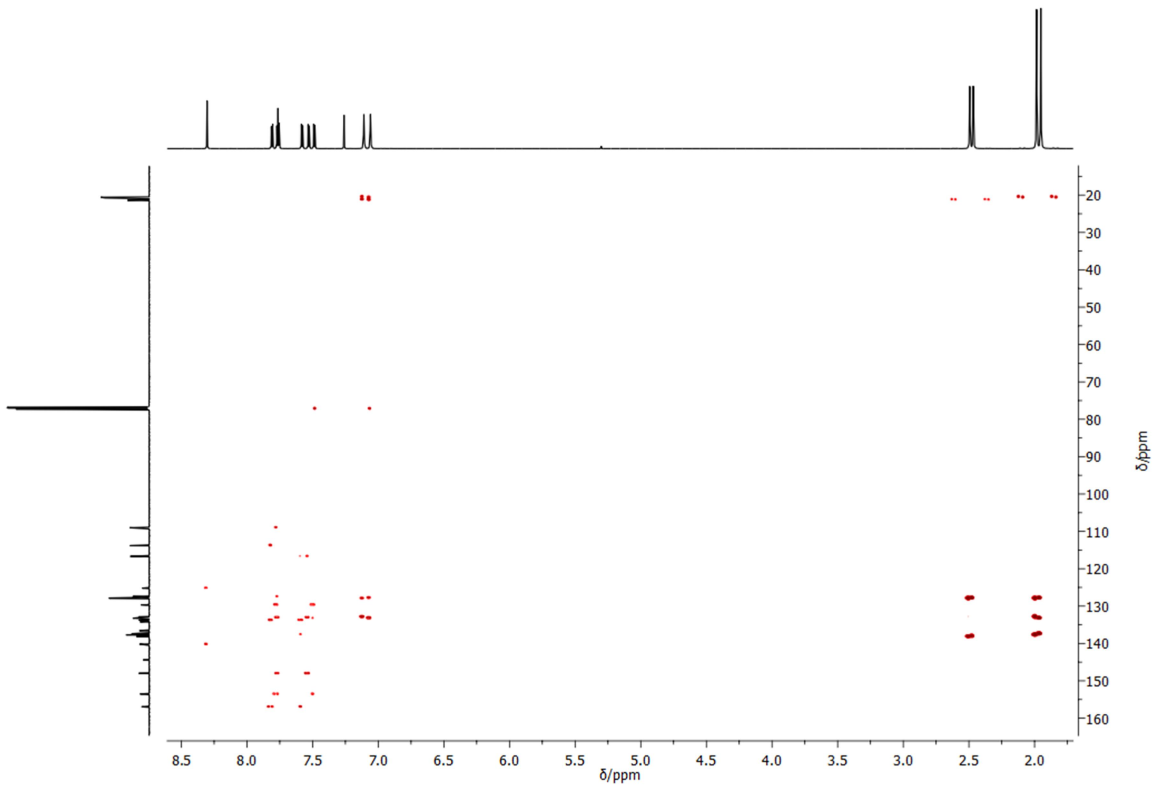

Figure S5. ^{1}H,^{13}C HMBC spectrum (600/150 MHz, $CDCl_3$, 300 K) of **2-NO$_2$**.

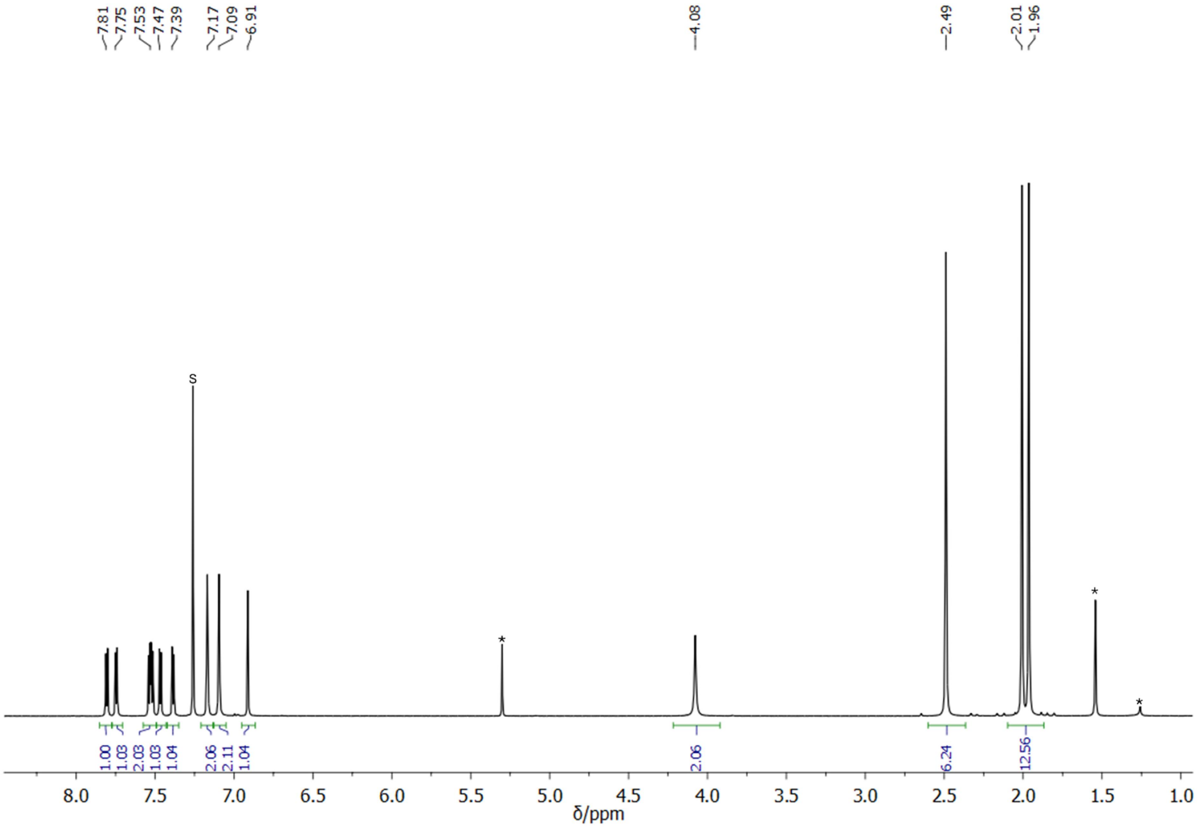

Figure S6. ^{1}H NMR (400 MHz, $CDCl_3$, 300 K) of **2-NH$_2$**.

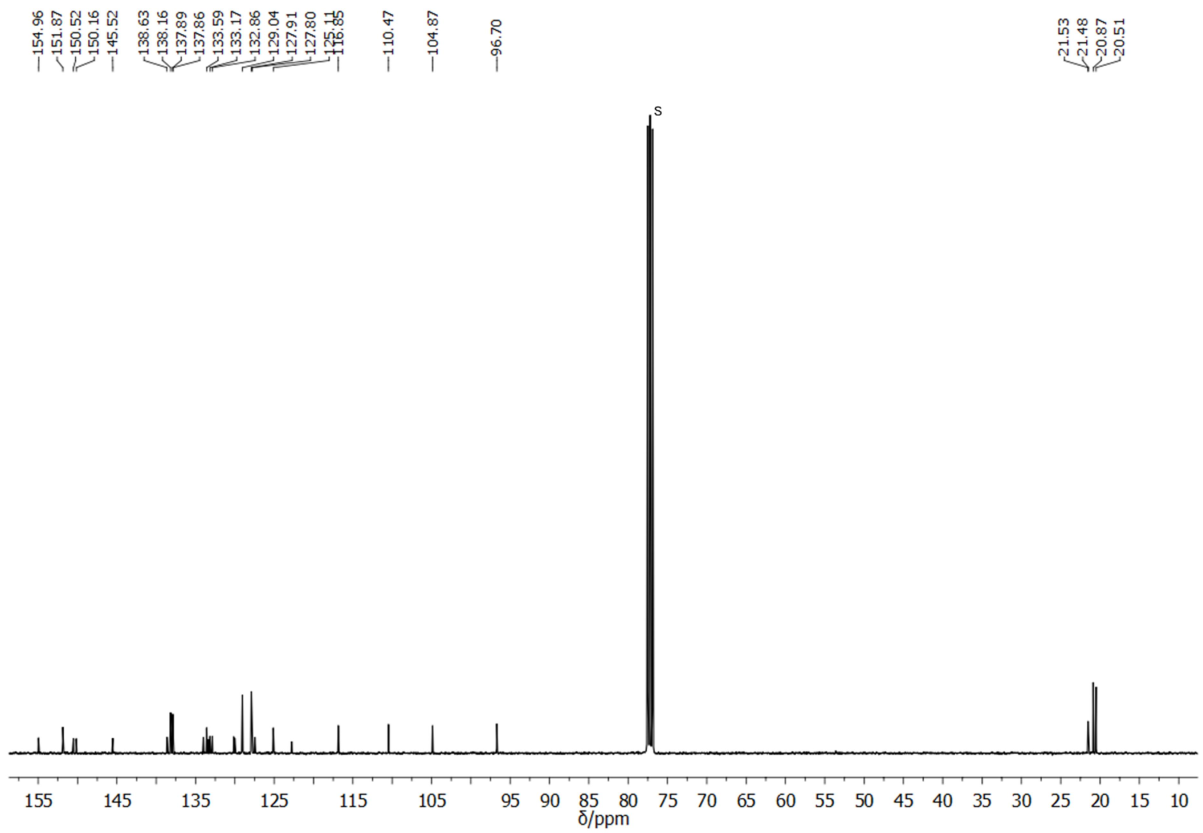

Figure S7. ^{13}C NMR (100 MHz, $CDCl_3$, 300 K) of **2-NH$_2$**.

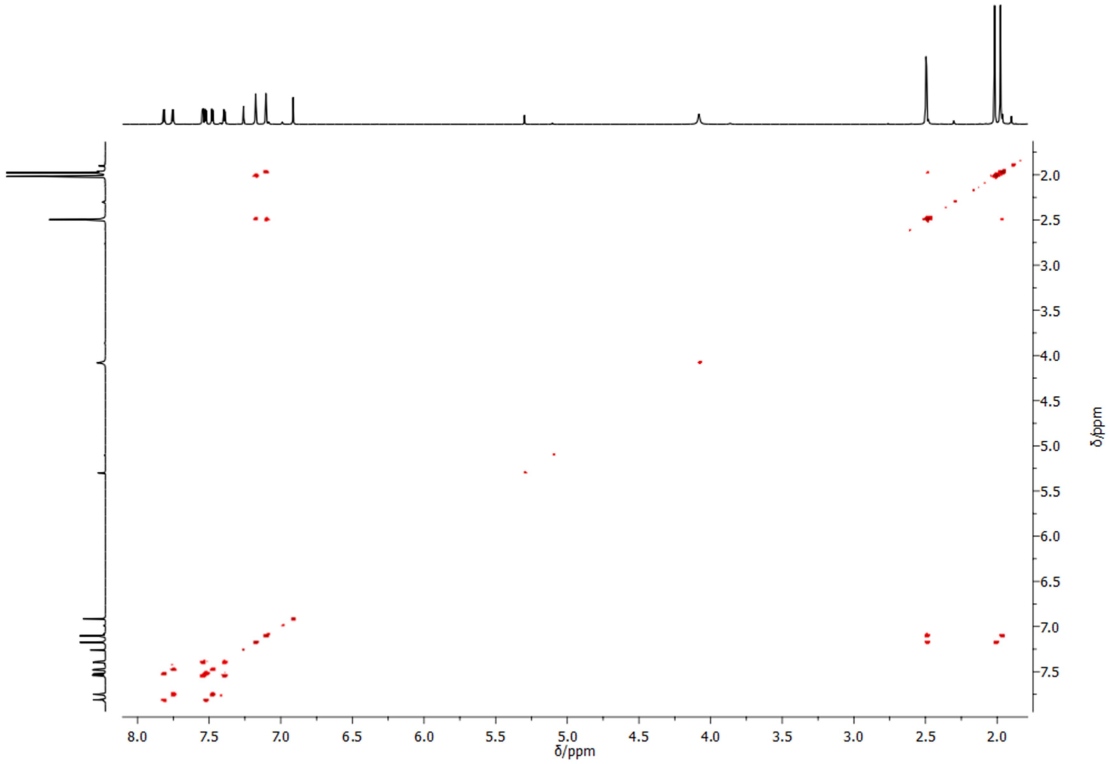

Figure S8. ^{1}H-^{1}H COSY (600 MHz, $CDCl_3$, 300 K) of **2-NH$_2$**.

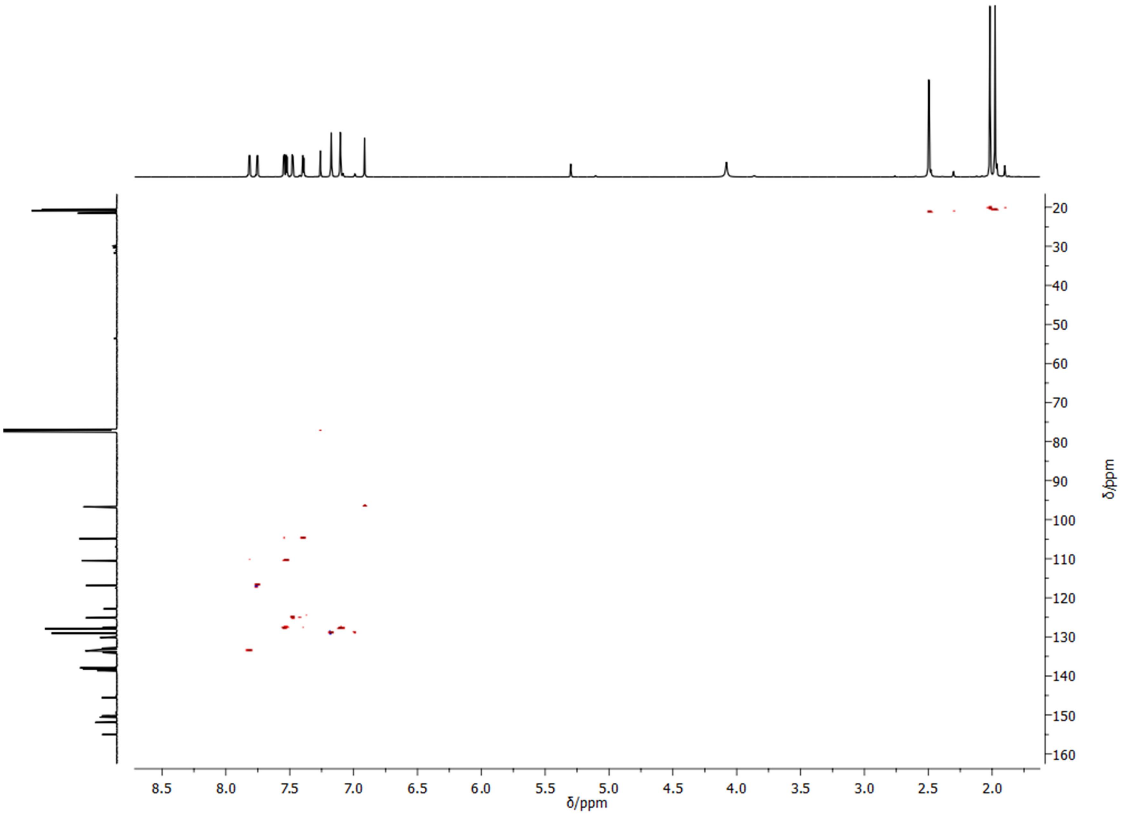

Figure S9. $^{1}H,^{13}C$ HSQC spectrum (600/150 MHz, $CDCl_3$, 300 K) of **2-NH$_2$**.

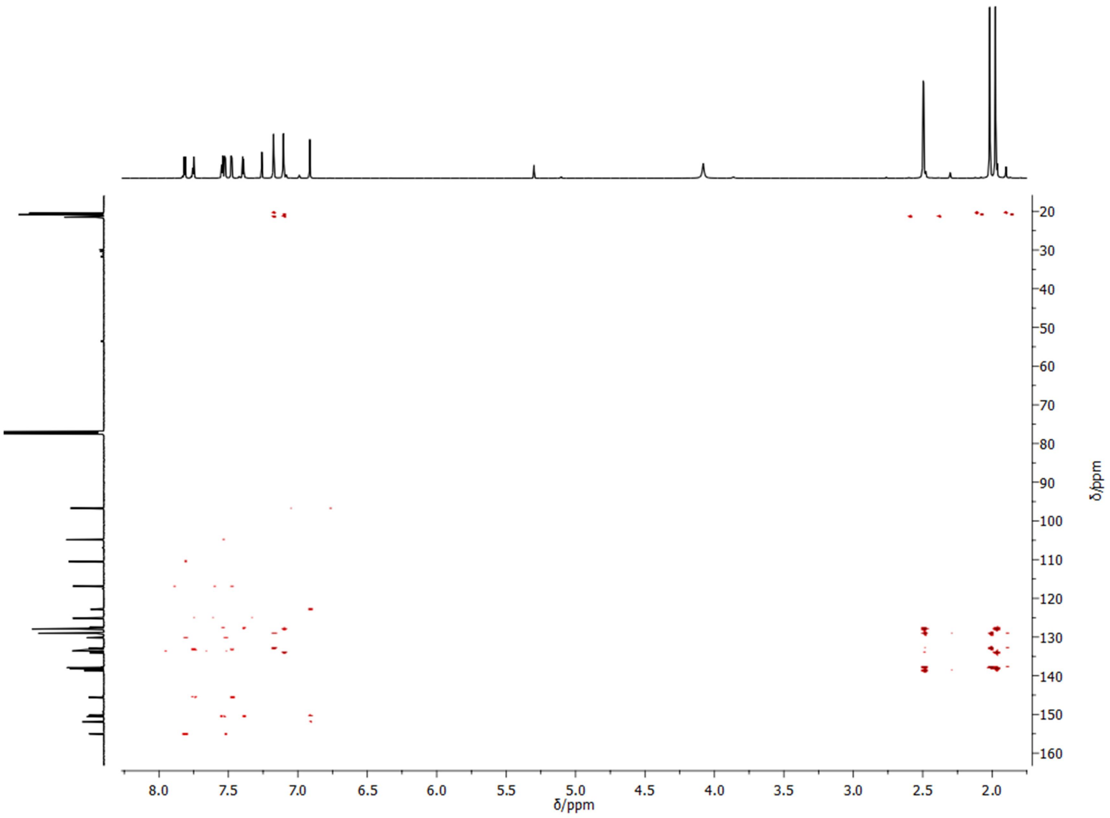

Figure S10. $^{1}H,^{13}C$ HMBC spectrum (600/150 MHz, $CDCl_3$, 300 K) of **2-NH$_2$**.

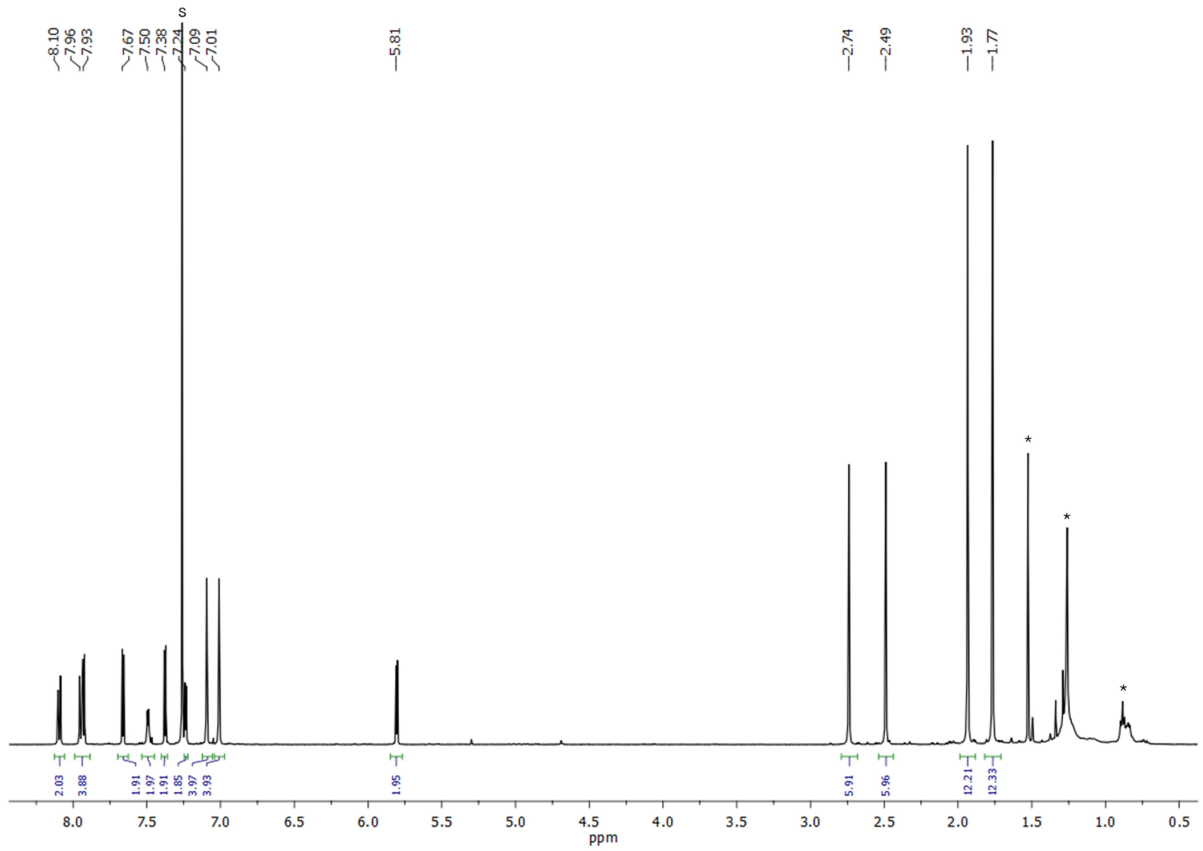

Figure S11. ^{1}H NMR spectrum (600 MHz, $CDCl_3$, 300 K) of **3a**.

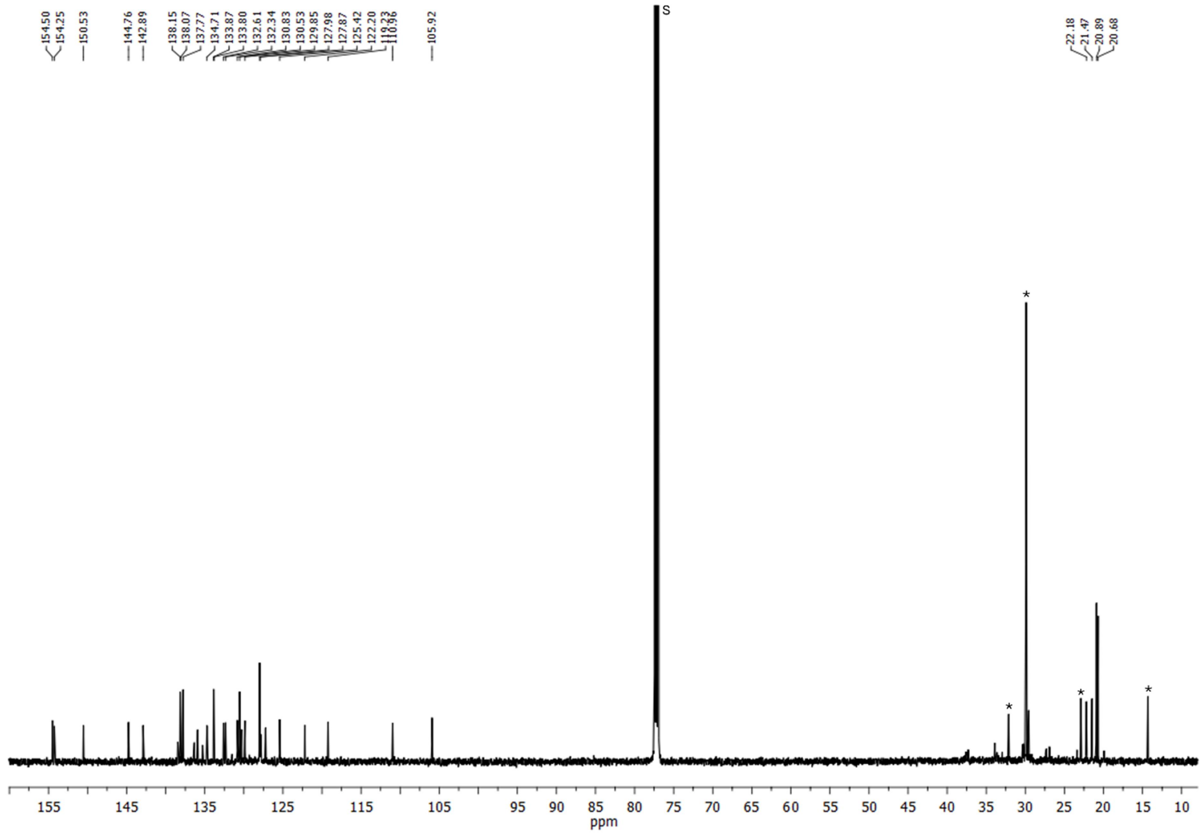

Figure S12. ^{13}C NMR spectrum (150 MHz, $CDCl_3$, 300 K) of **3a**.

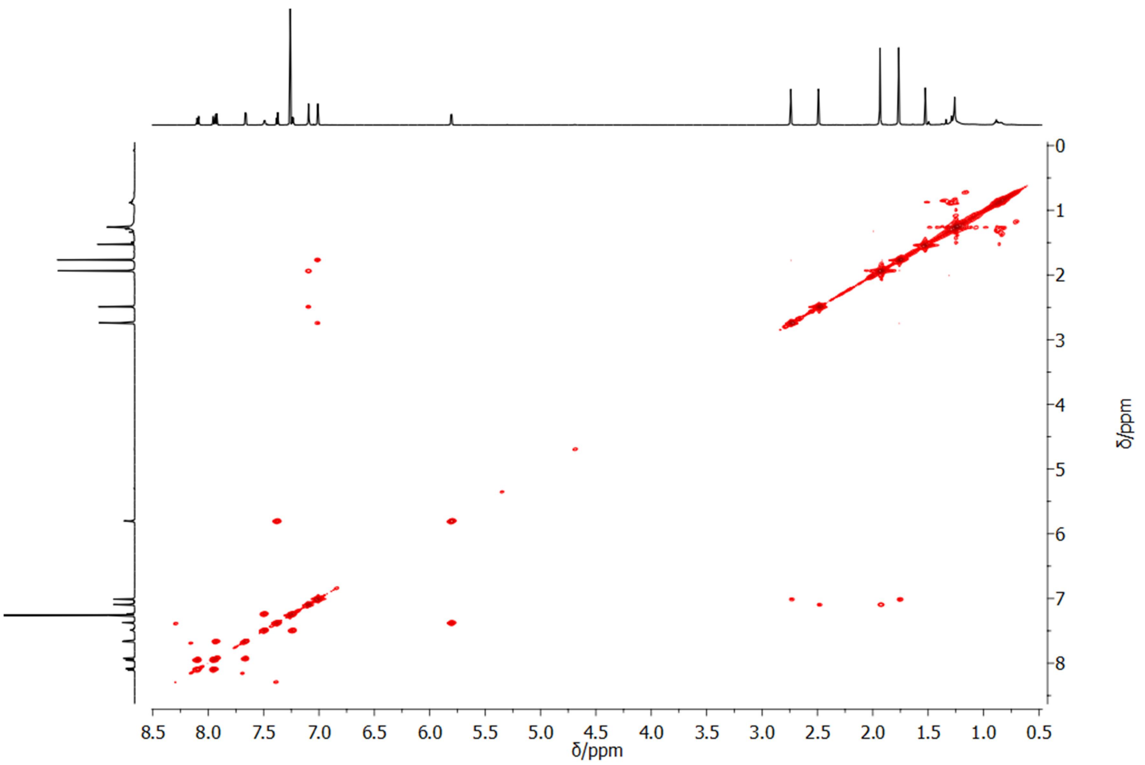

Figure S13. $^{1}H,^{1}H$ COSY spectrum (600 MHz, $CDCl_3$, 300 K) of **3a**.

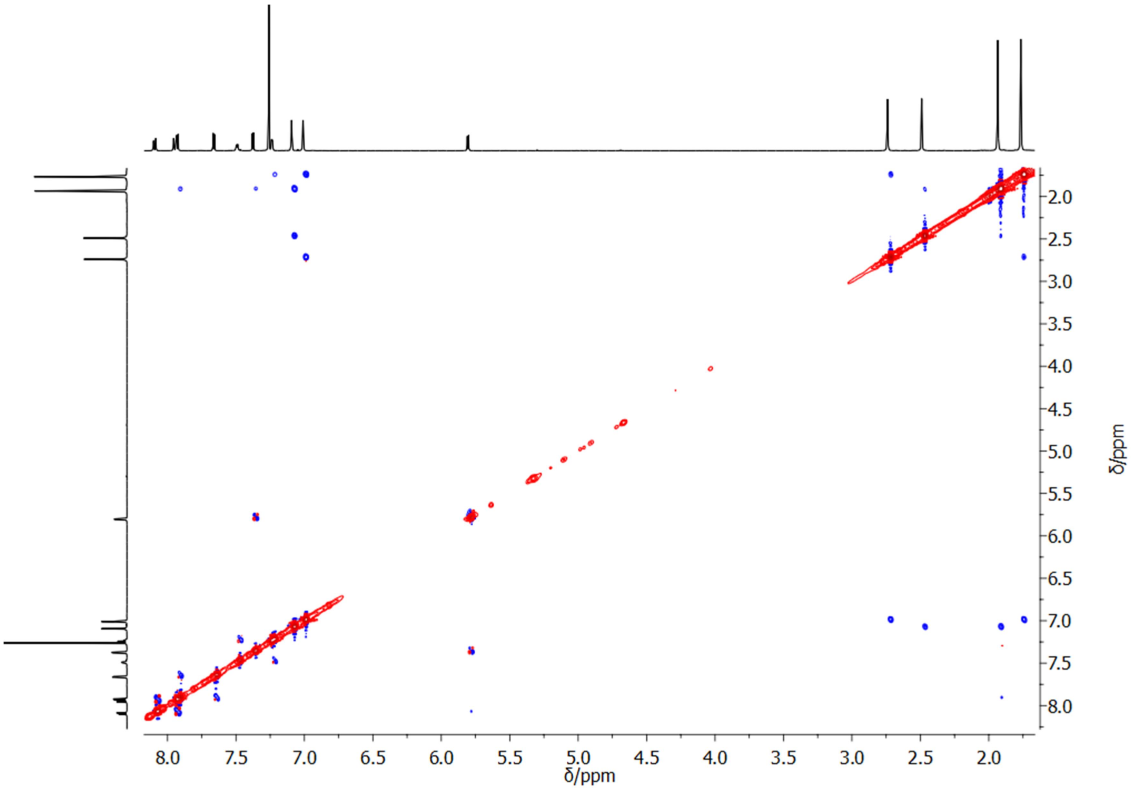

Figure S14. $^{1}H,^{1}H$ ROESY spectrum (600 MHz, $CDCl_3$, 300 K) of **3a**.

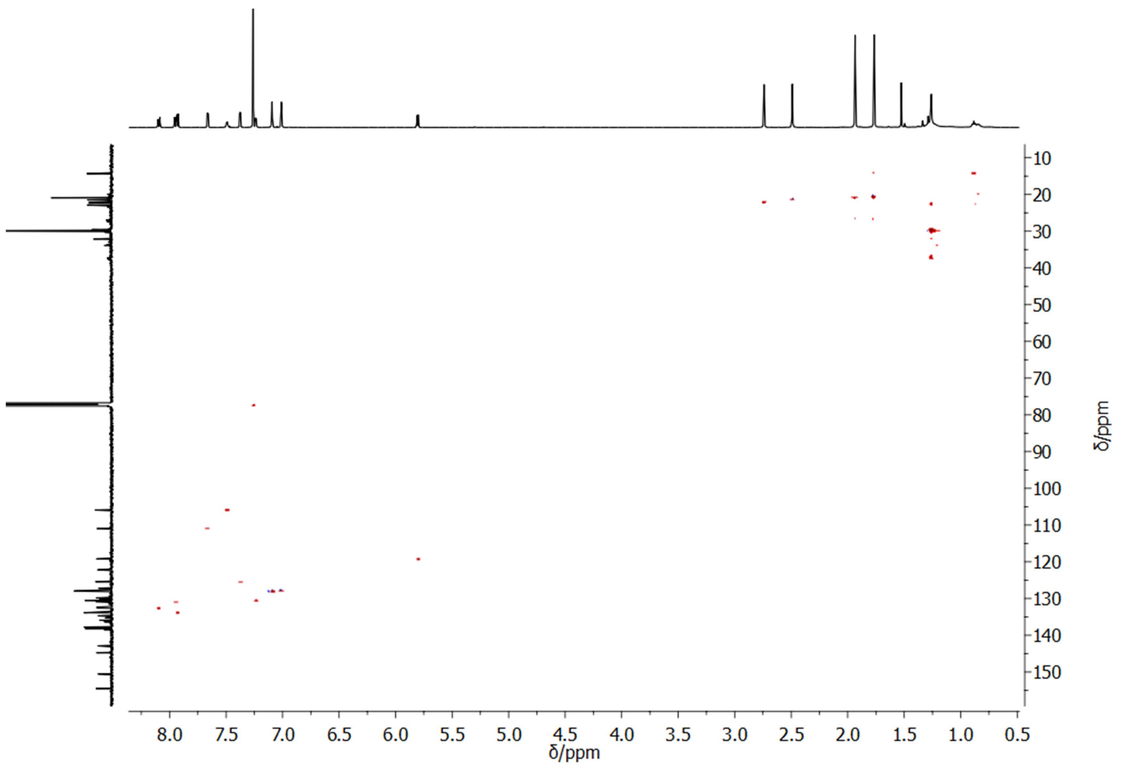

Figure S15. $^{1}H,^{13}C$ HSQC spectrum (600/150 MHz, $CDCl_3$, 300 K) of **3a**.

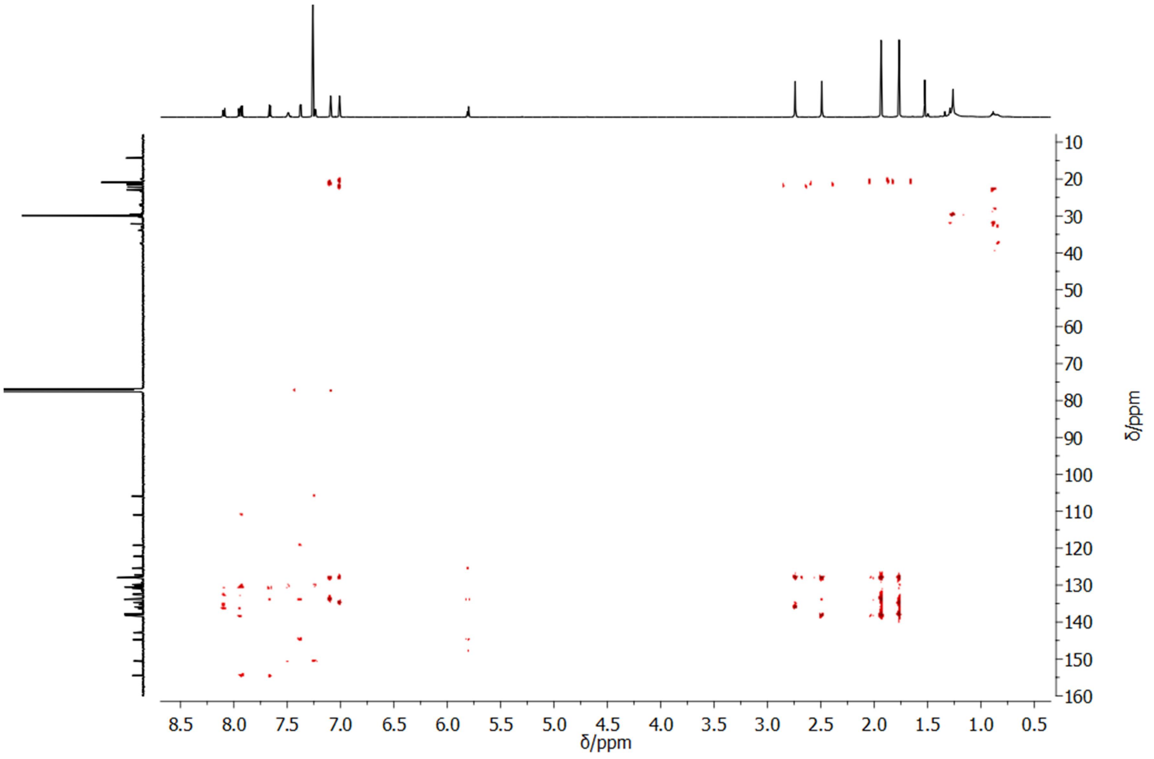

Figure S16. $^{1}H,^{13}C$ HMBC spectrum (600/150 MHz, $CDCl_3$, 300 K) of **3a**.

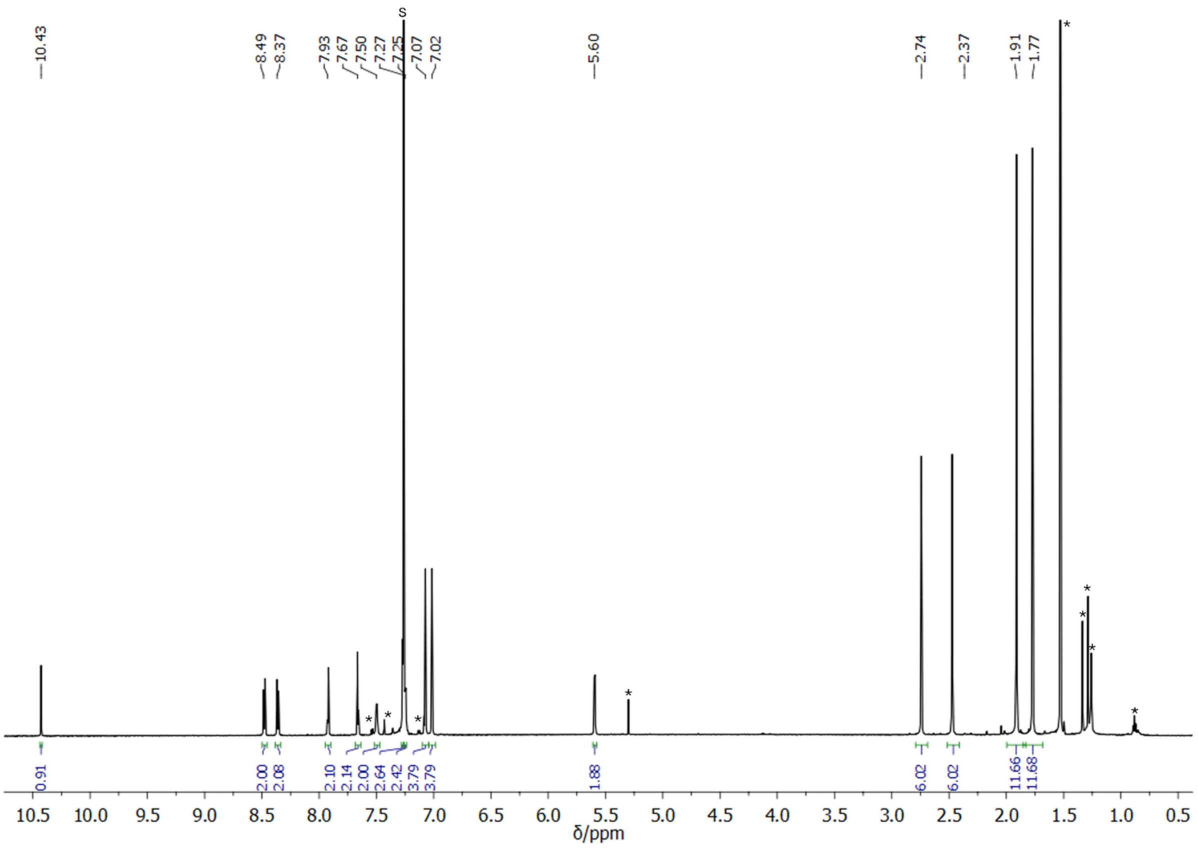

Figure S17. ^{1}H NMR spectrum (600 MHz, $CDCl_3$, 300 K) of **3b**.

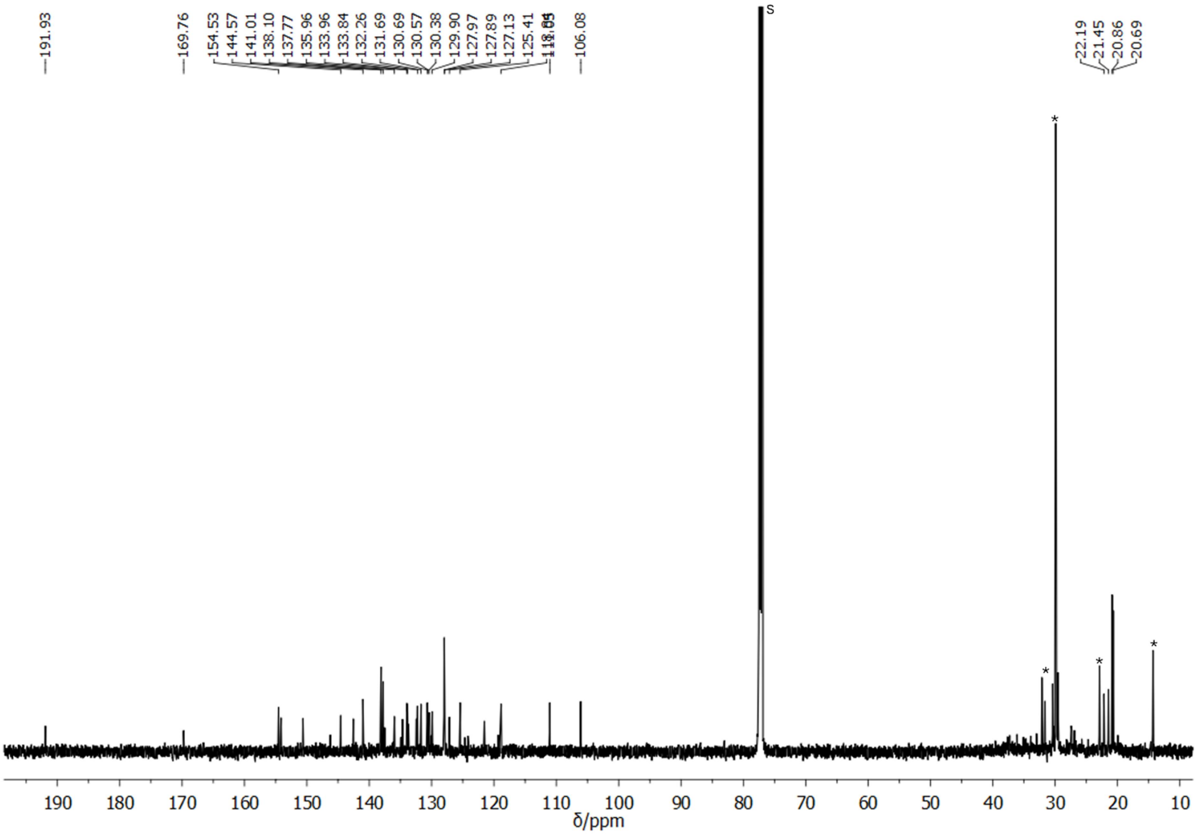

Figure S18. ^{13}C NMR spectrum (150 MHz, $CDCl_3$, 300 K) of **3b**.

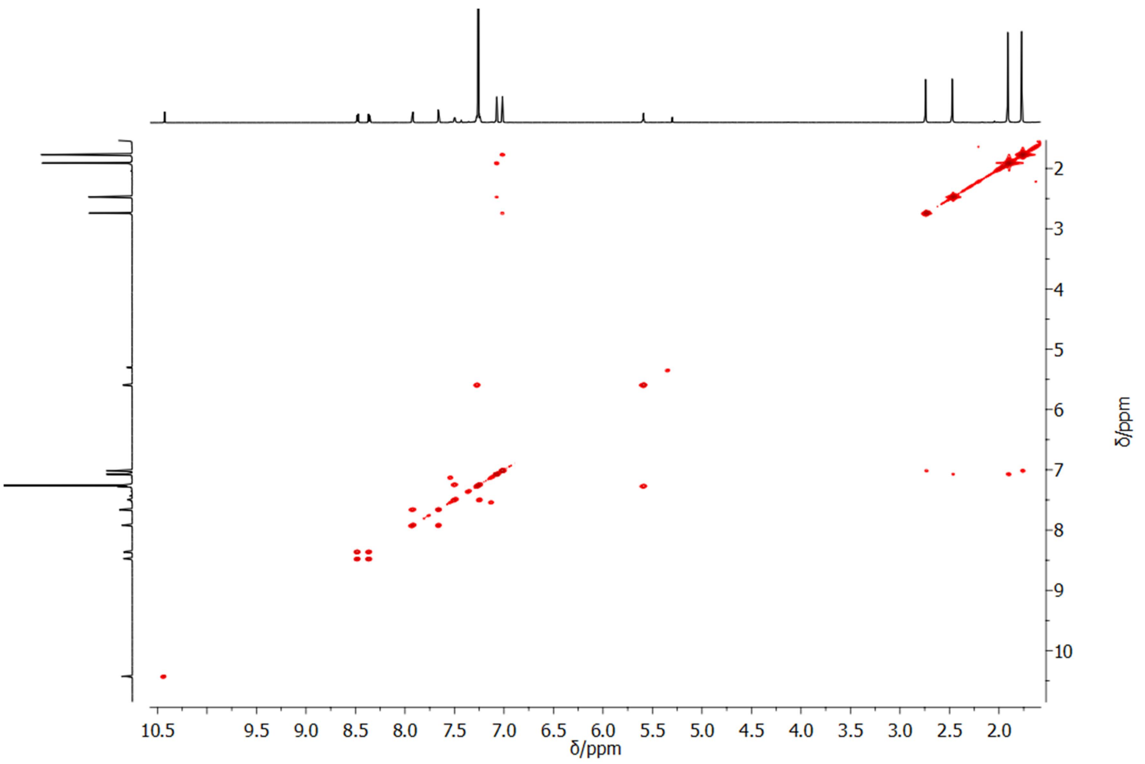

Figure S19. ^{1}H,^{1}H COSY spectrum (600 MHz, $CDCl_3$, 300 K) of **3b**.

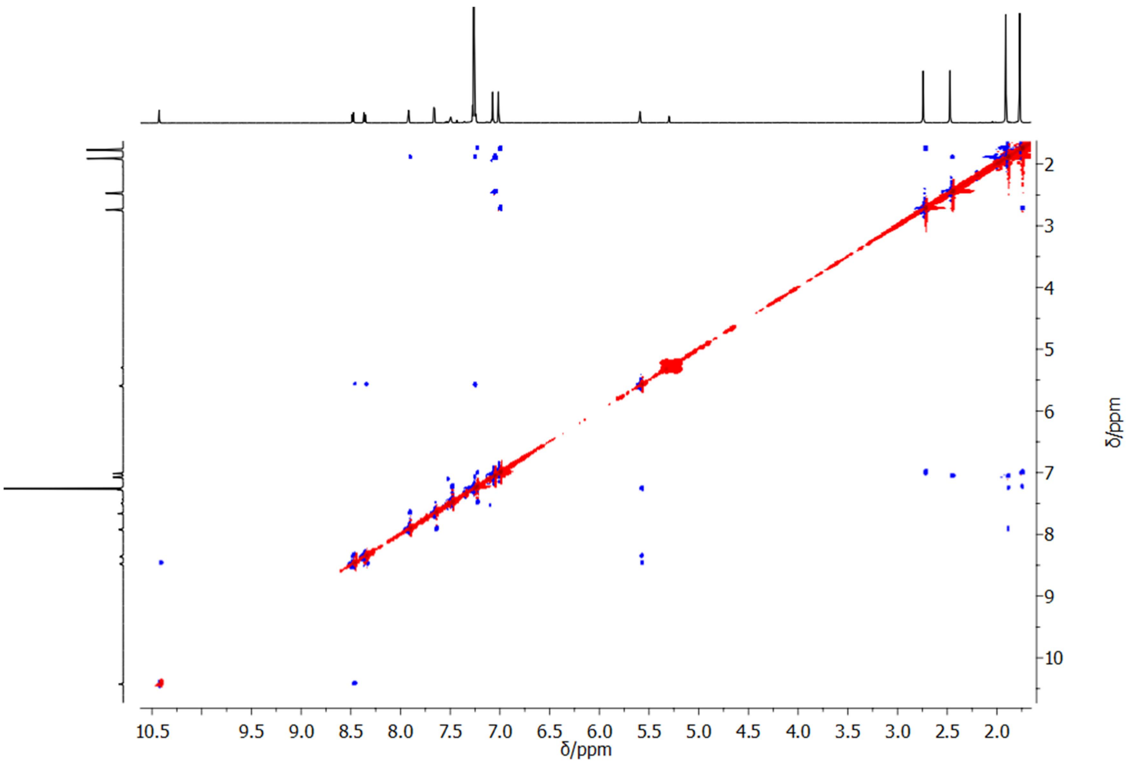

Figure S20. ^{1}H,^{1}H ROESY spectrum (600 MHz, $CDCl_3$, 300 K) of **3b**.

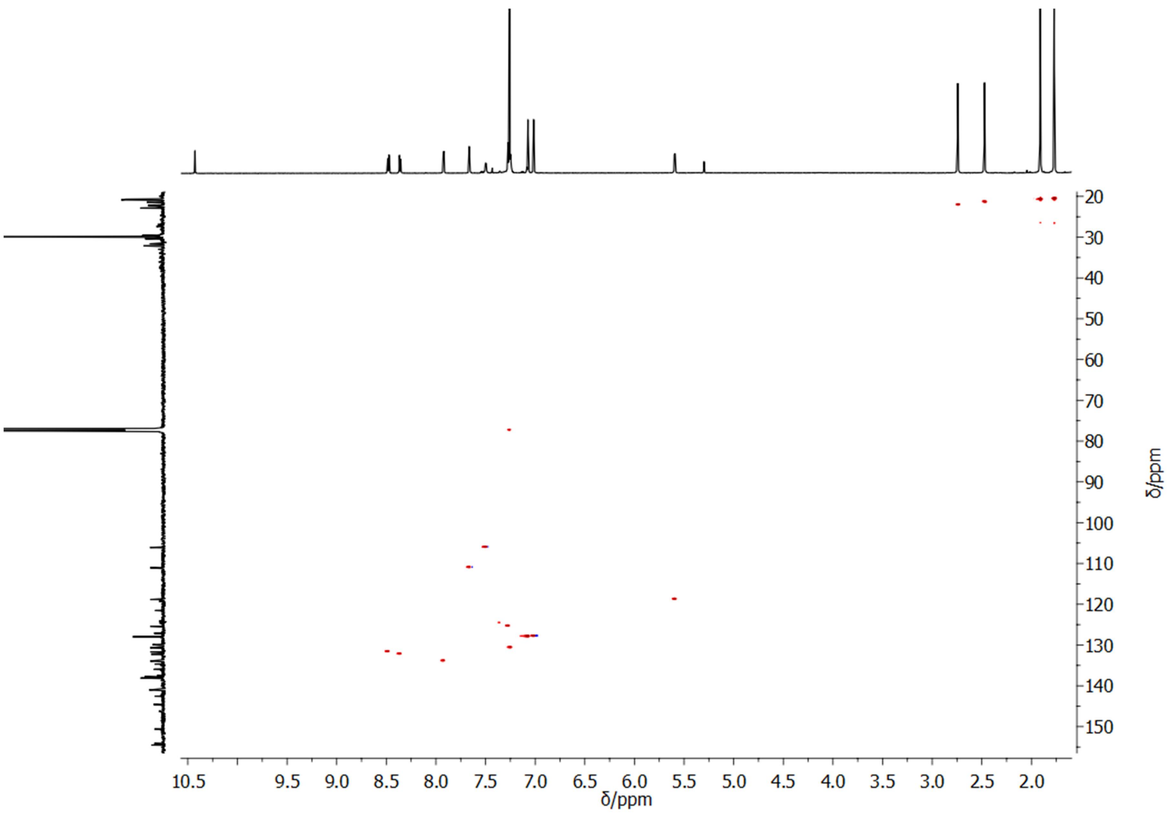

Figure S21. $^{1}H,^{13}C$ HSQC spectrum (600/150 MHz, $CDCl_3$, 300 K) of **3b**.

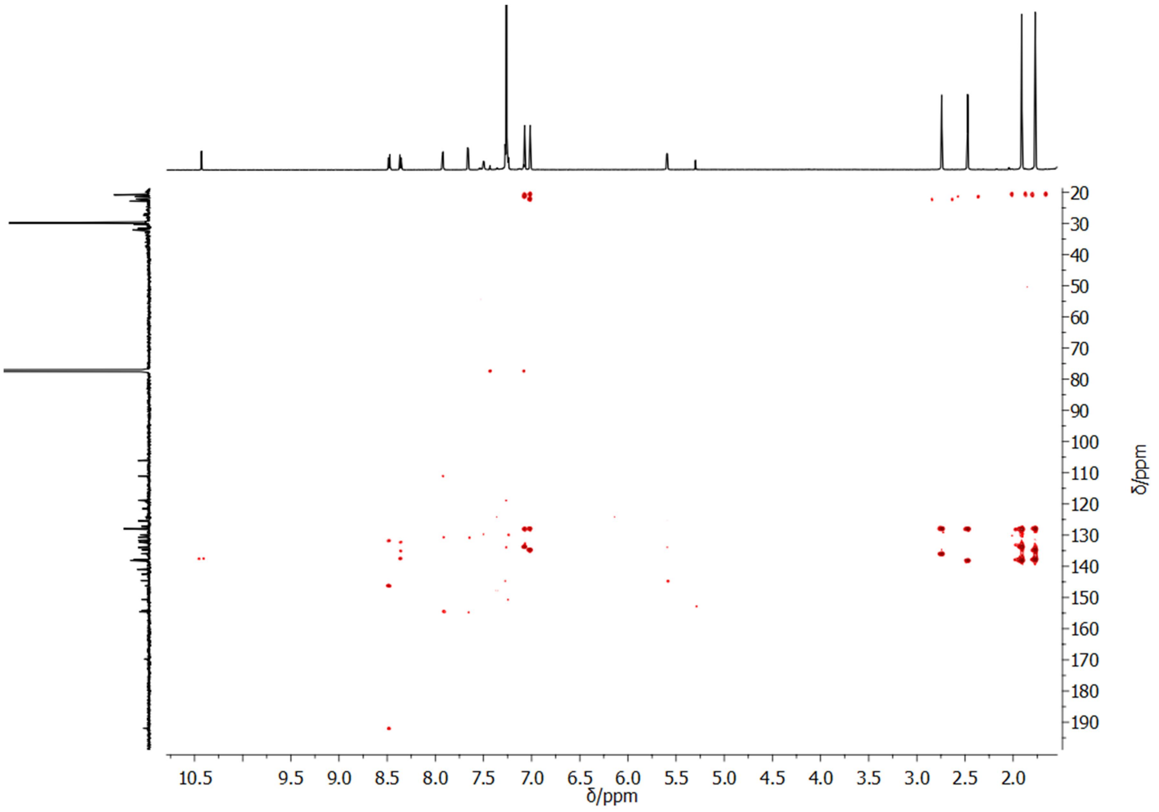

Figure S22. $^{1}H,^{13}C$ HMBC spectrum (600/150 MHz, $CDCl_3$, 300 K) of **3b**.

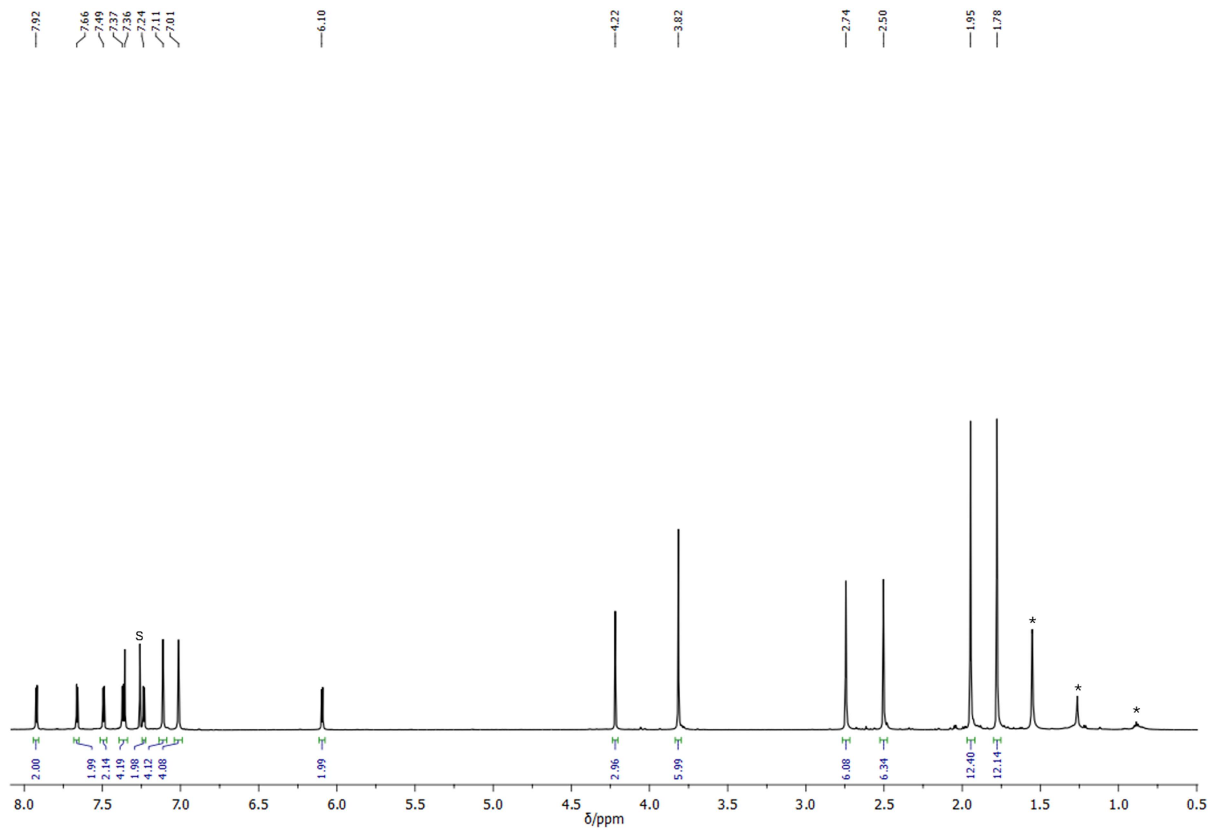

Figure S23. ^{1}H NMR spectrum (600 MHz, $CDCl_3$, 300 K) of **3c**.

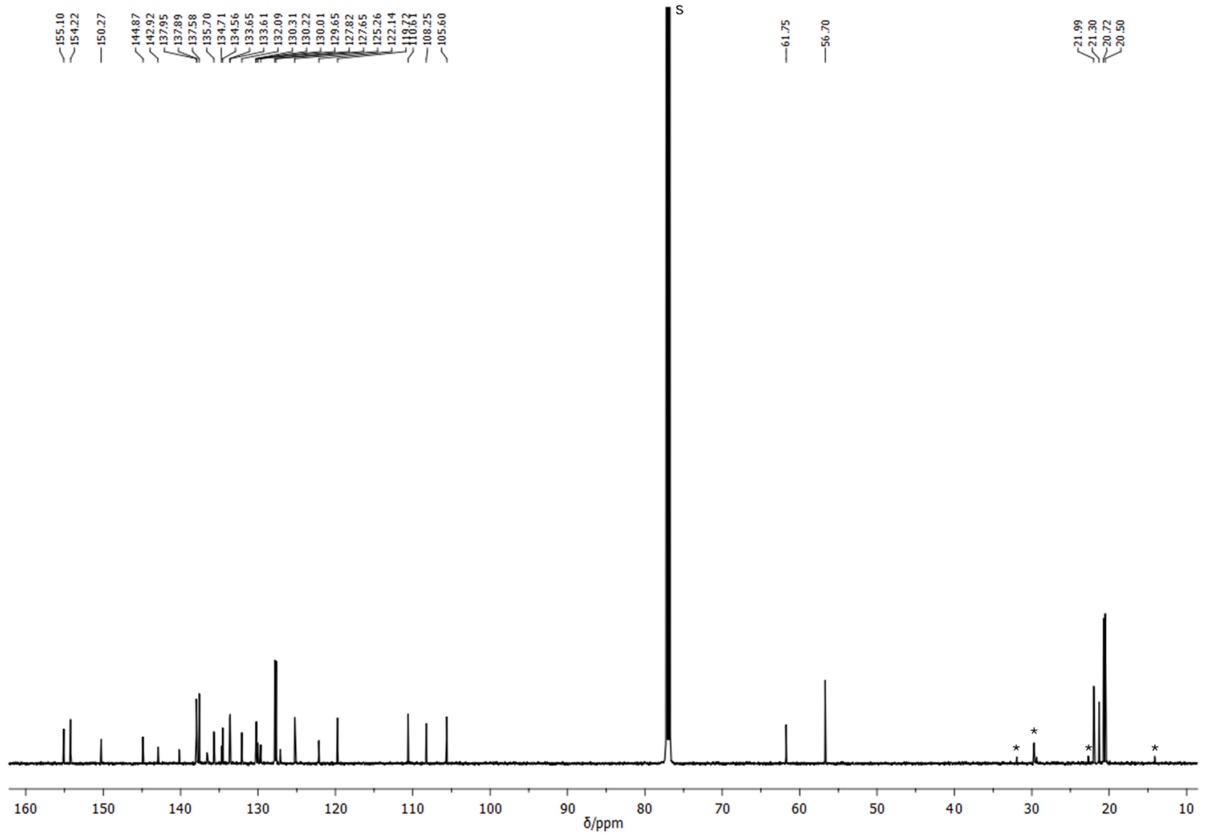

Figure S24. ^{13}C NMR spectrum (150 MHz, $CDCl_3$, 300 K) of **3c**.

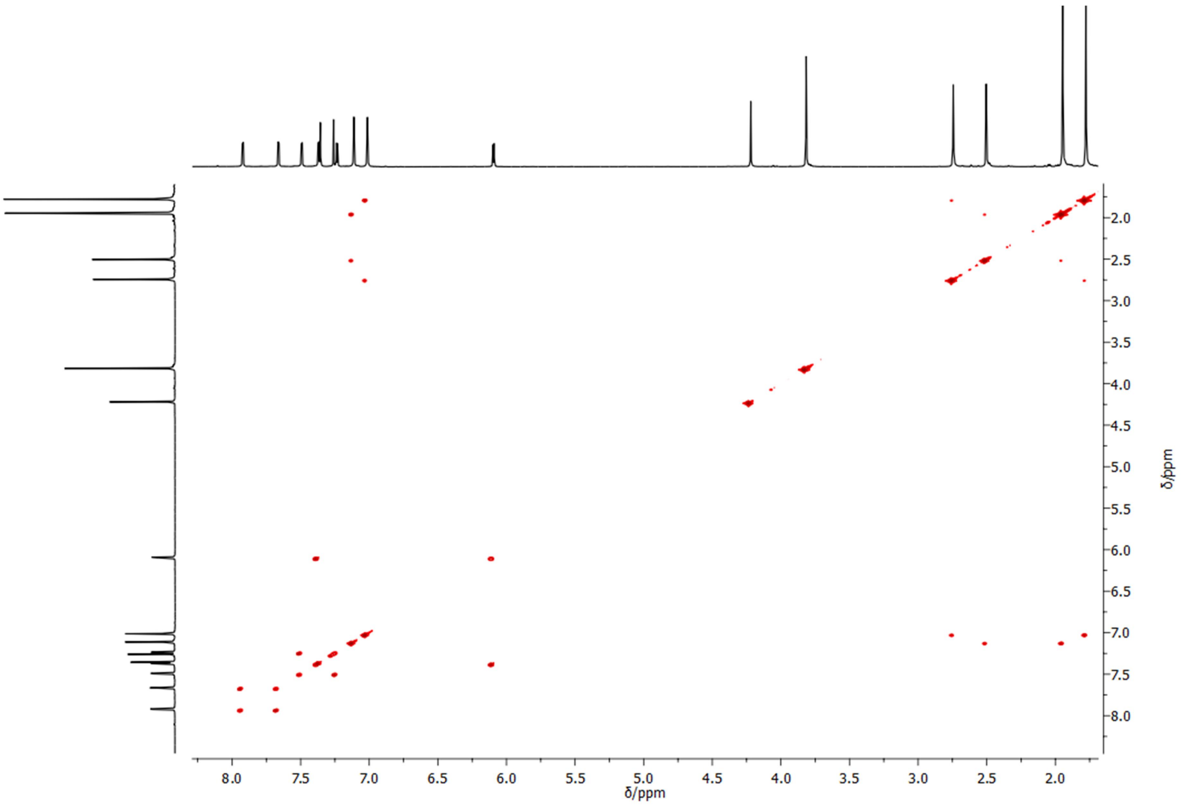

Figure S25. $^{1}H,^{1}H$ COSY spectrum (600 MHz, $CDCl_3$, 300 K) of **3c**.

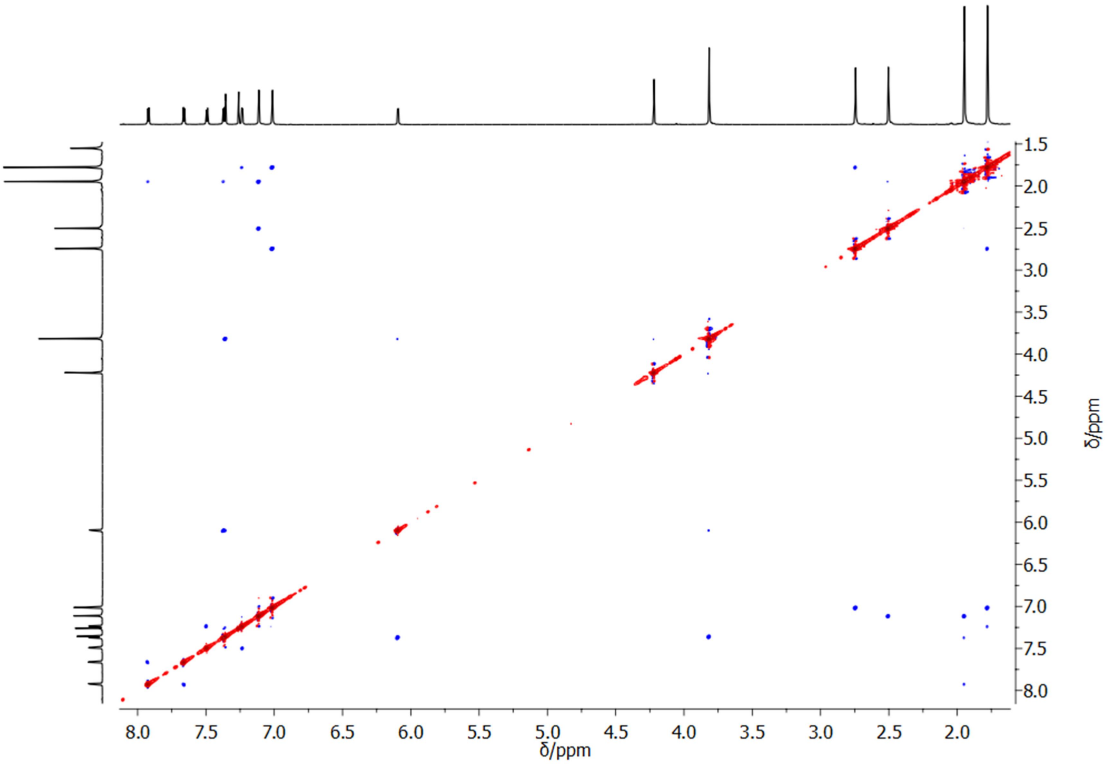

Figure S26. $^{1}H,^{1}H$ ROESY spectrum (600 MHz, $CDCl_3$, 300 K) of **3c**.

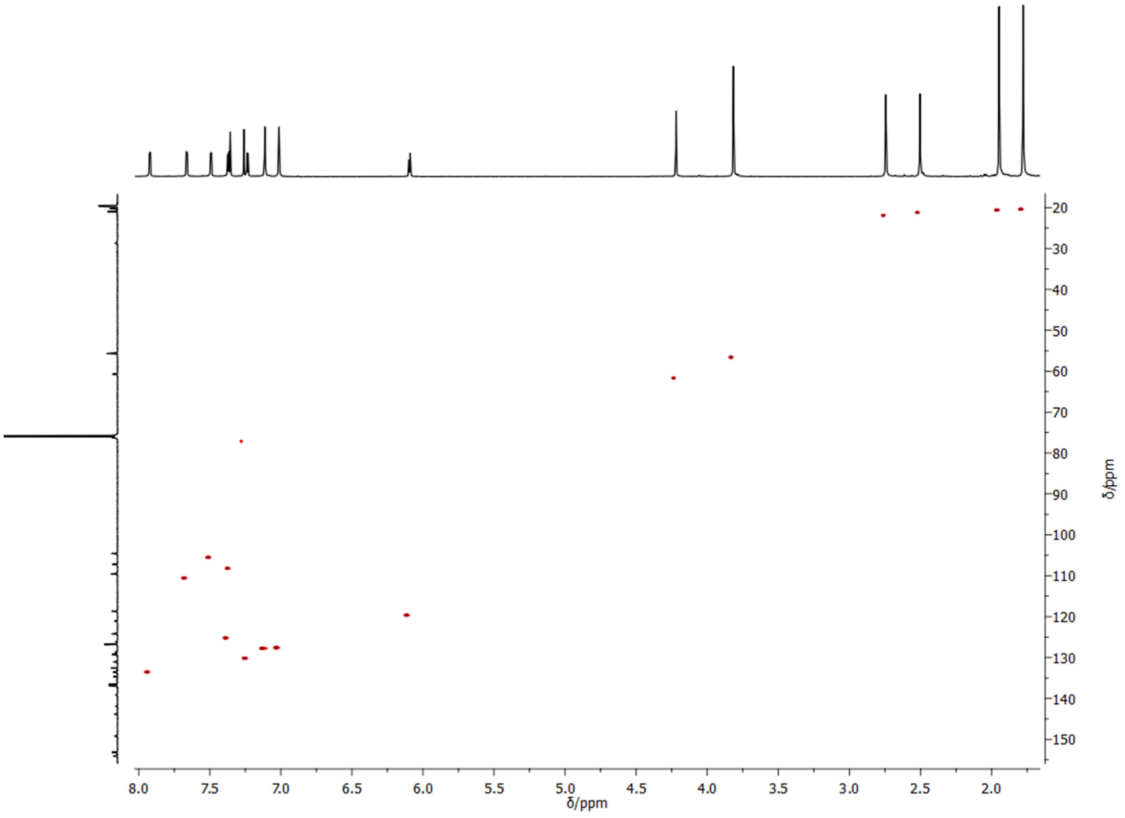

Figure S27. $^{1}H,^{13}C$ HSQC spectrum (600/150 MHz, $CDCl_3$, 300 K) of **3c**.

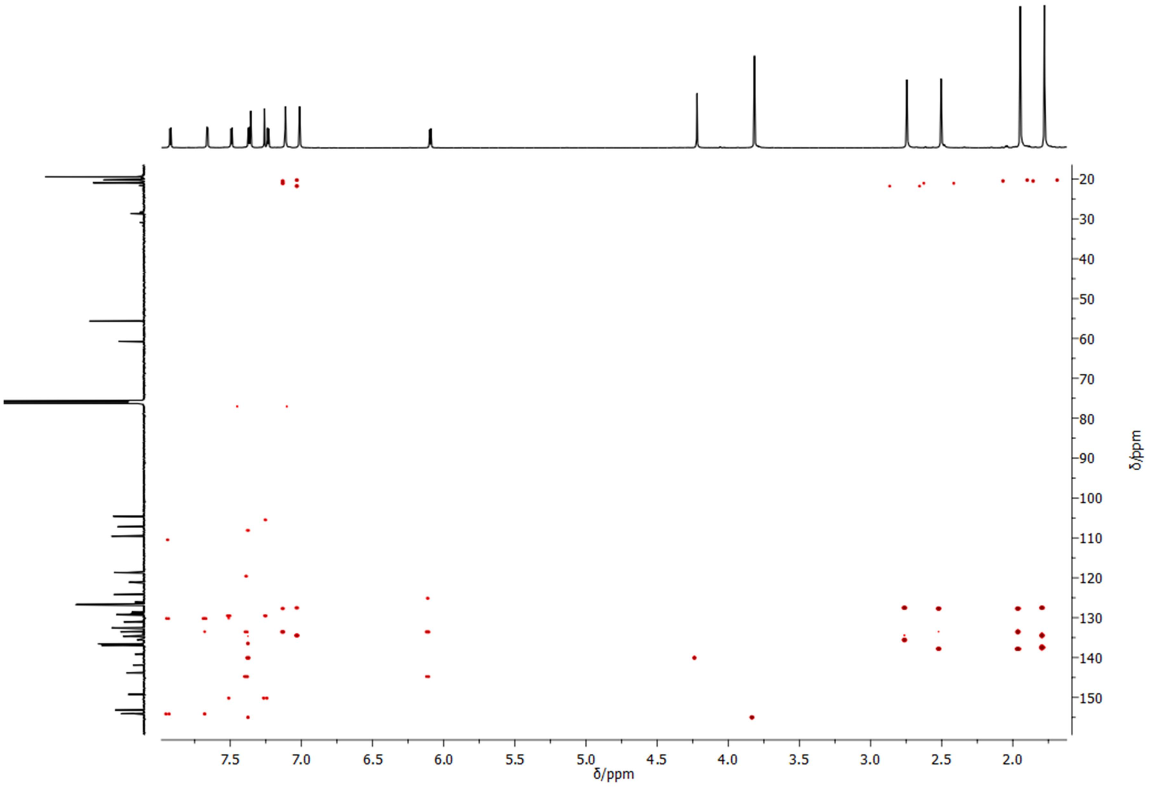

Figure S28. $^{1}H,^{13}C$ HMBC spectrum (600/150 MHz, $CDCl_3$, 300 K) of **3c**.

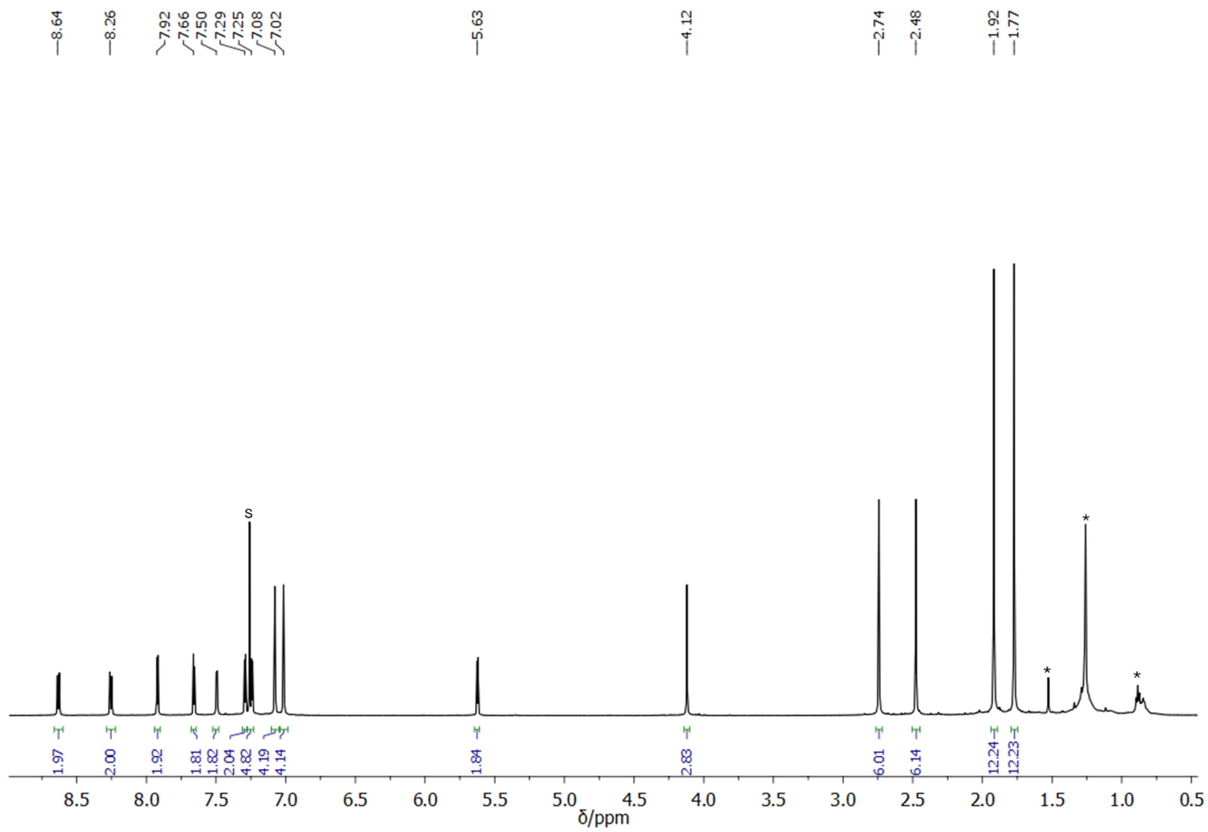

Figure S29. ^{1}H NMR spectrum (600 MHz, $CDCl_3$, 300 K) of **3d**.

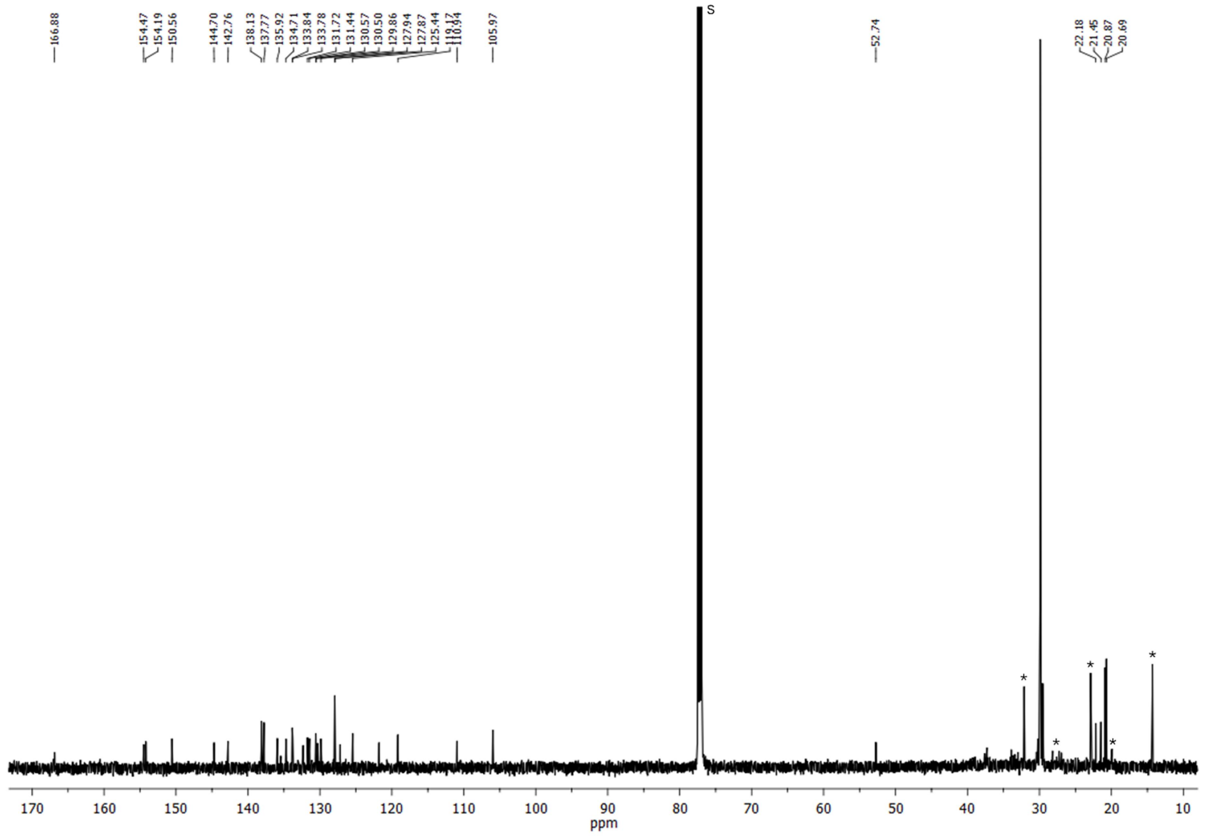

Figure S30. ^{13}C NMR spectrum (150 MHz, $CDCl_3$, 300 K) of **3d**.

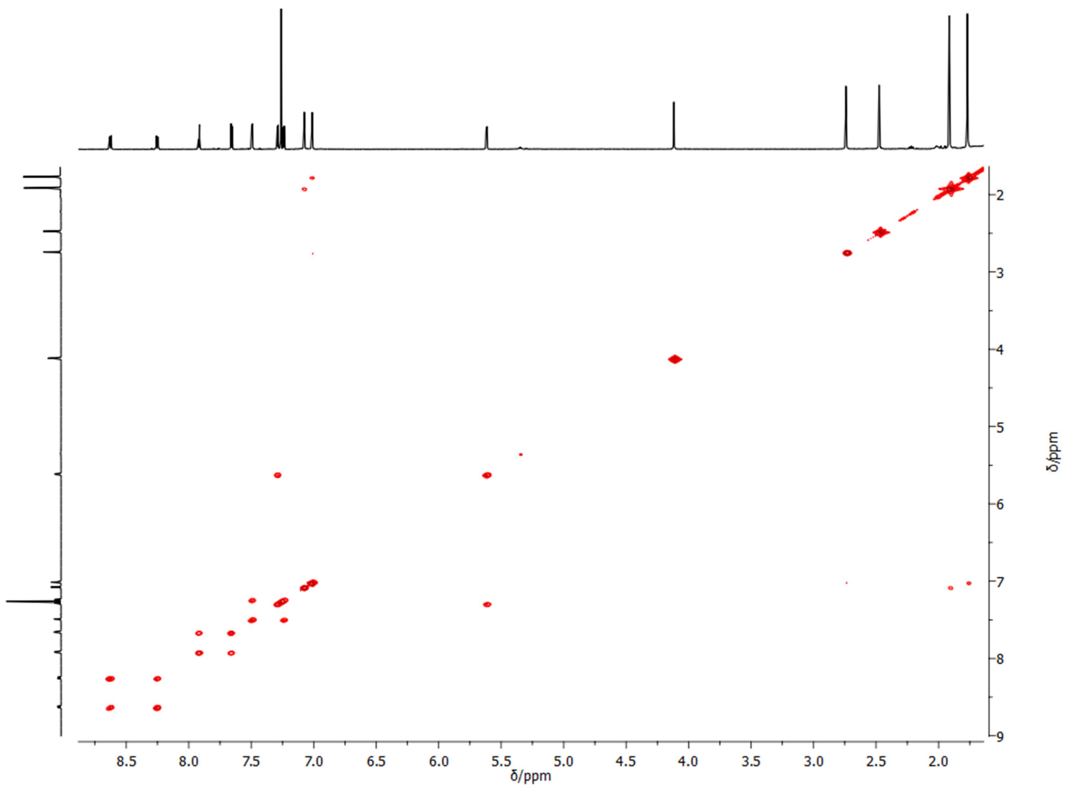

Figure S31. ^{1}H,^{1}H COSY spectrum (600 MHz, $CDCl_3$, 300 K) of **3d**.

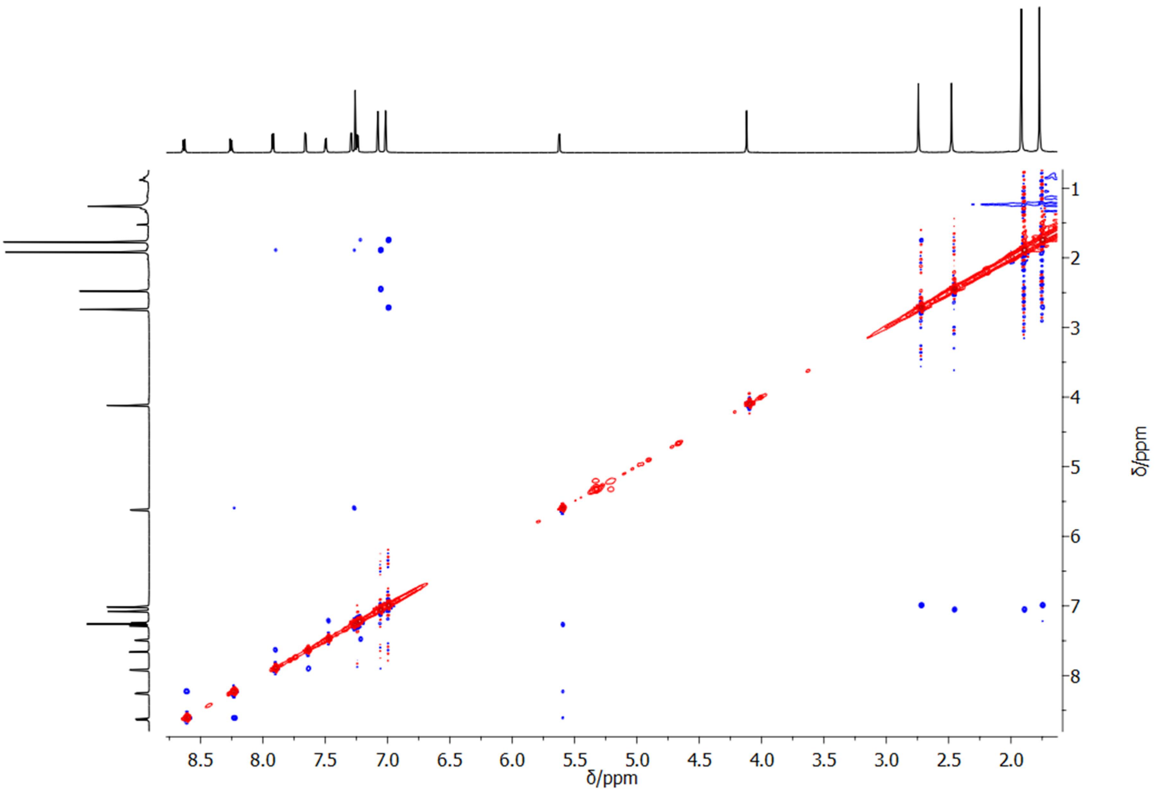

Figure S32. ^{1}H,^{1}H ROESY spectrum (600 MHz, $CDCl_3$, 300 K) of **3d**.

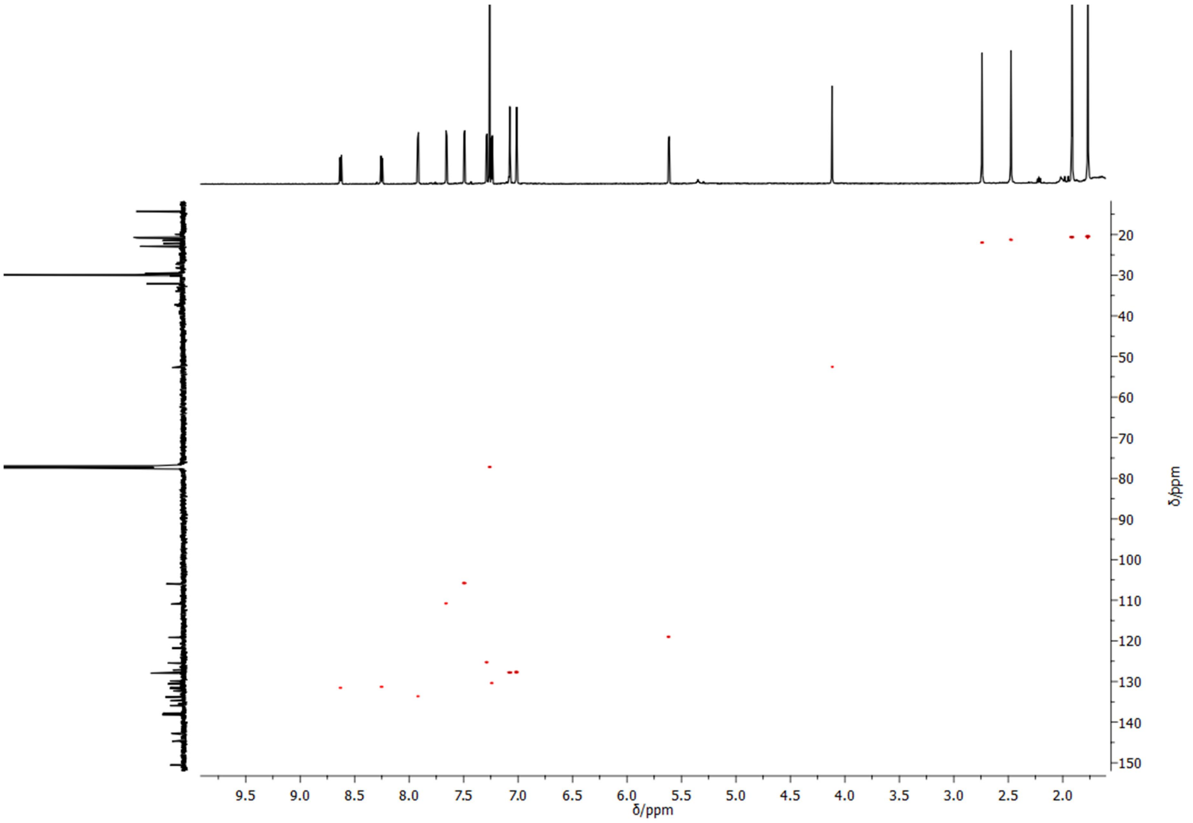

Figure S33. ^{1}H,^{13}C HSQC spectrum (600/150 MHz, $CDCl_3$, 300 K) of **3d**.

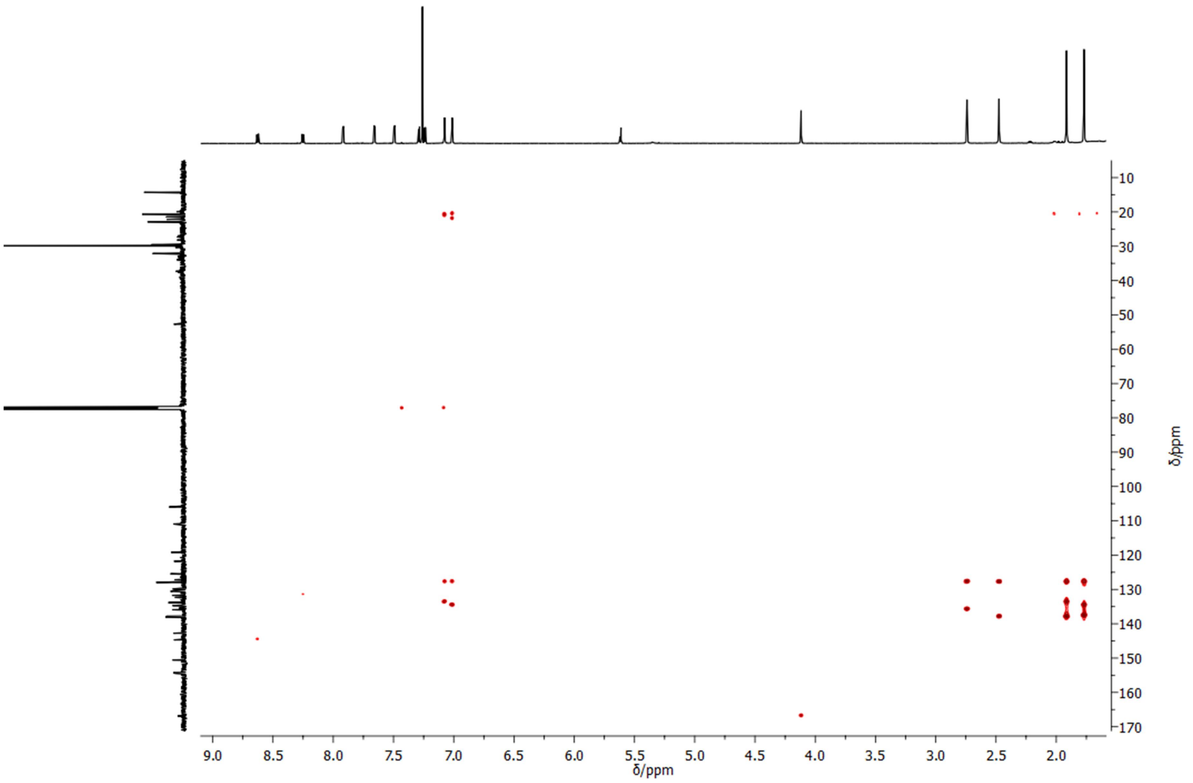

Figure S34. ^{1}H,^{13}C HMBC spectrum (600/150 MHz, $CDCl_3$, 300 K) of **3d**.

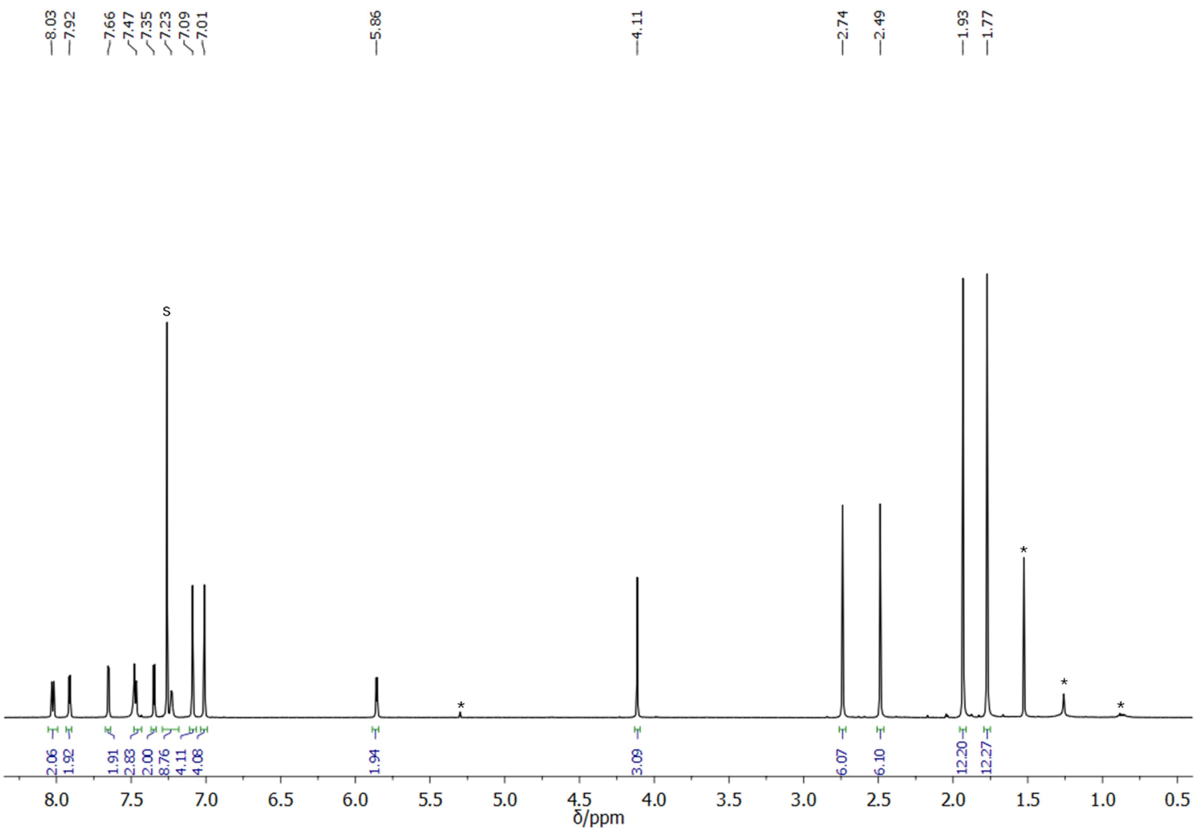

Figure S35. ^{1}H NMR spectrum (600 MHz, $CDCl_3$, 300 K) of **3e**.

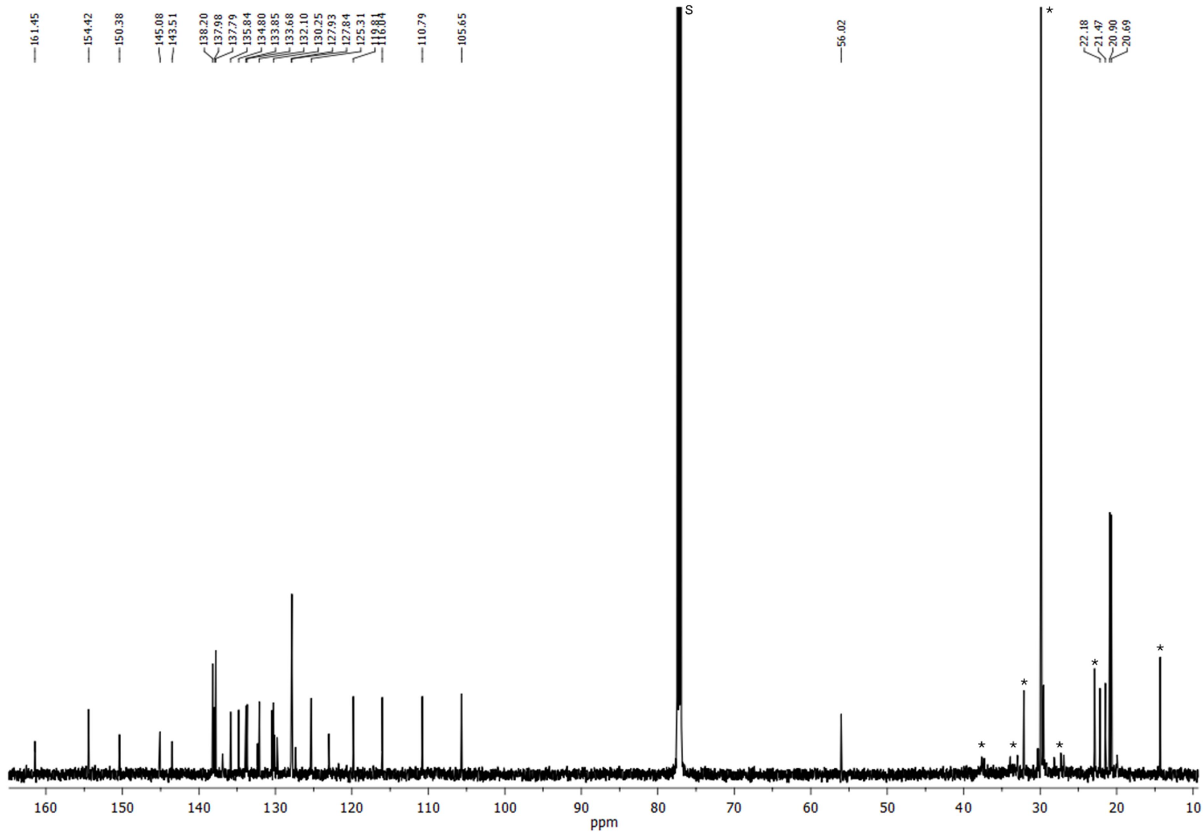

Figure S36. ^{13}C NMR spectrum (150 MHz, $CDCl_3$, 300 K) of **3e**.

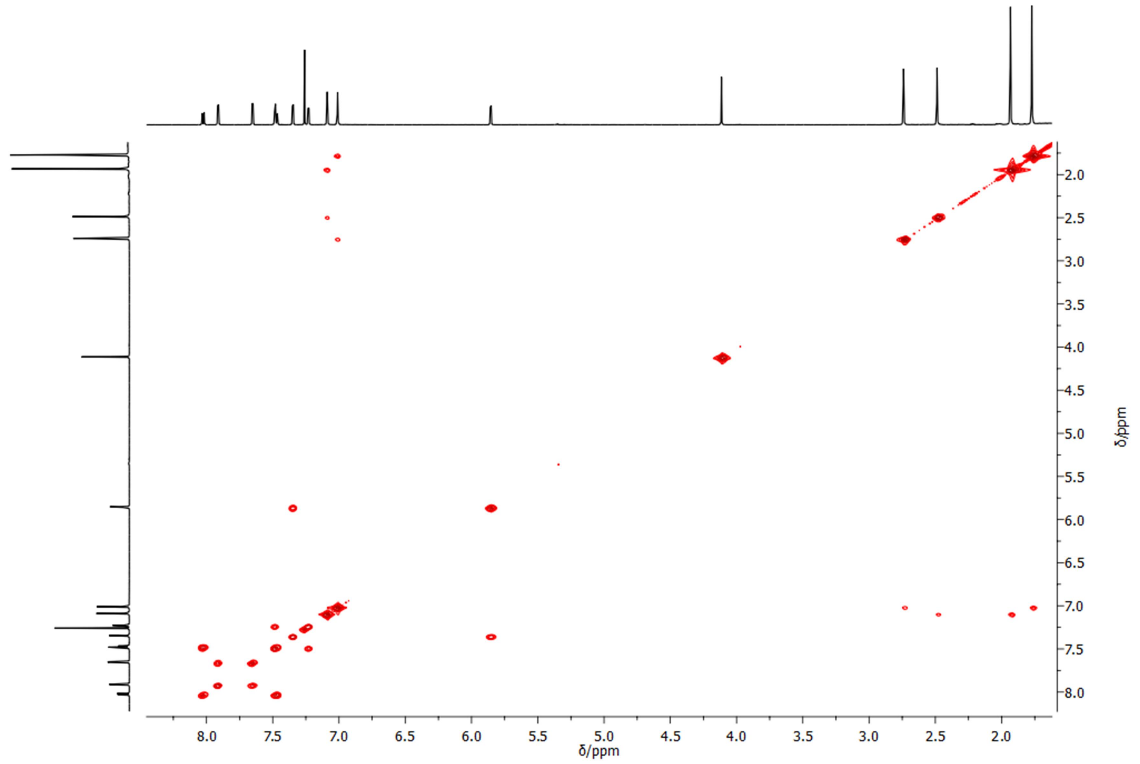

Figure S37. ^{1}H,^{1}H COSY spectrum (600 MHz, $CDCl_3$, 300 K) of **3e**.

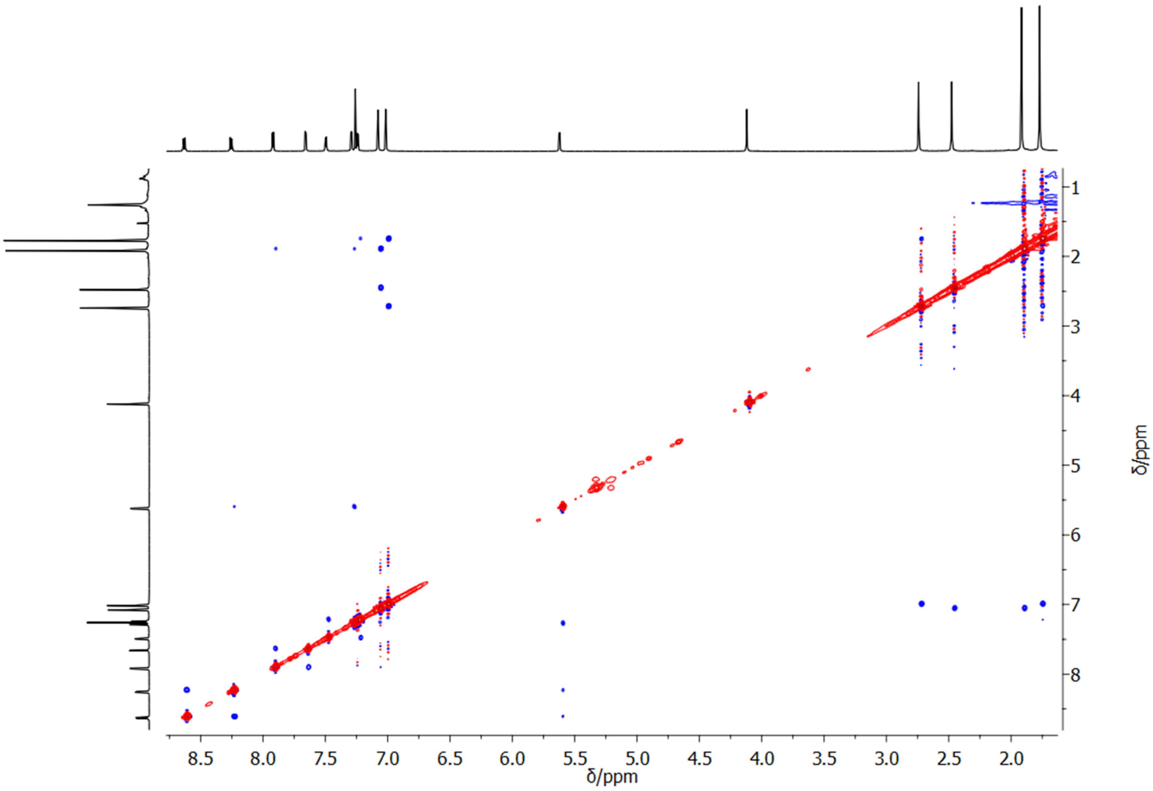

Figure S38. ^{1}H,^{1}H ROESY spectrum (600 MHz, $CDCl_3$, 300 K) of **3e**.

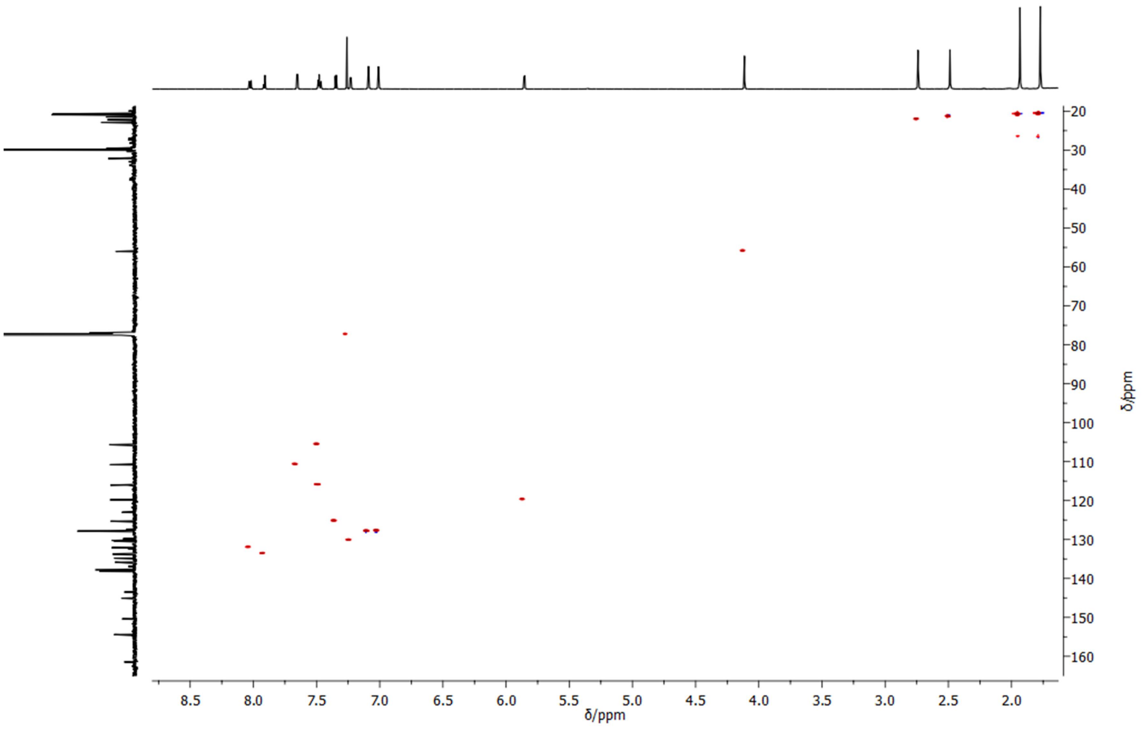

Figure S39. ^{1}H,^{13}C HSQC spectrum (600/150 MHz, $CDCl_3$, 300 K) of **3e**.

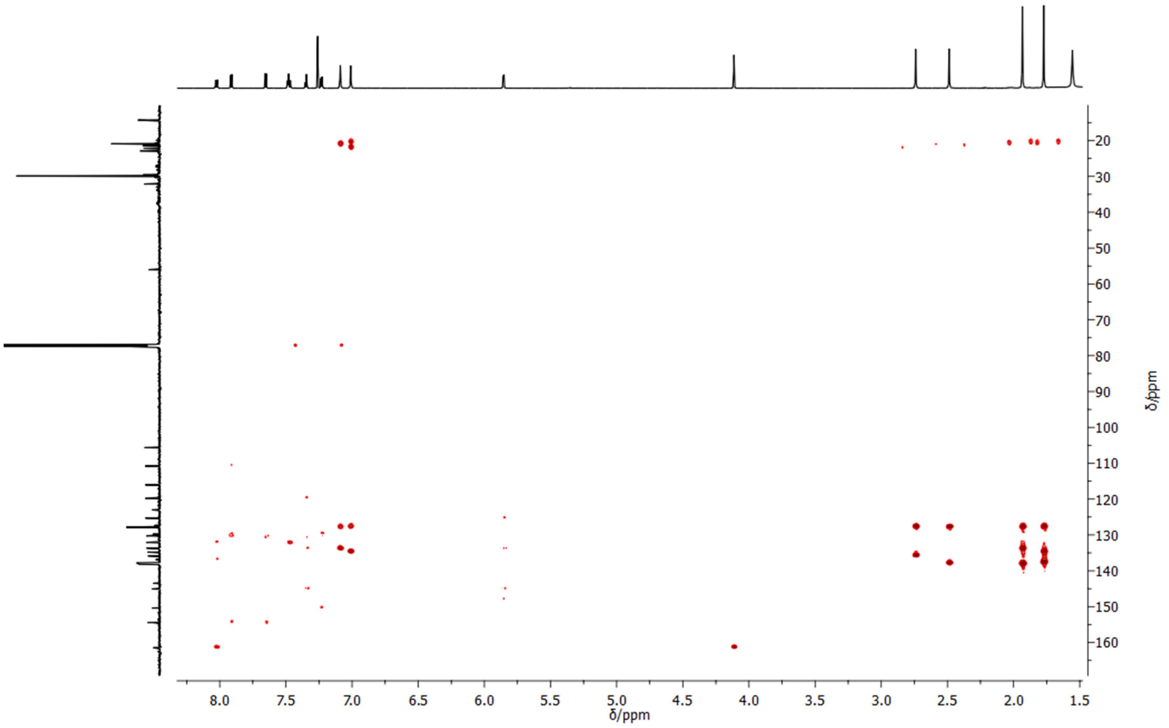

Figure S40. ^{1}H,^{13}C HMBC spectrum (600/150 MHz, CDCl3, 300 K) of **3e**.

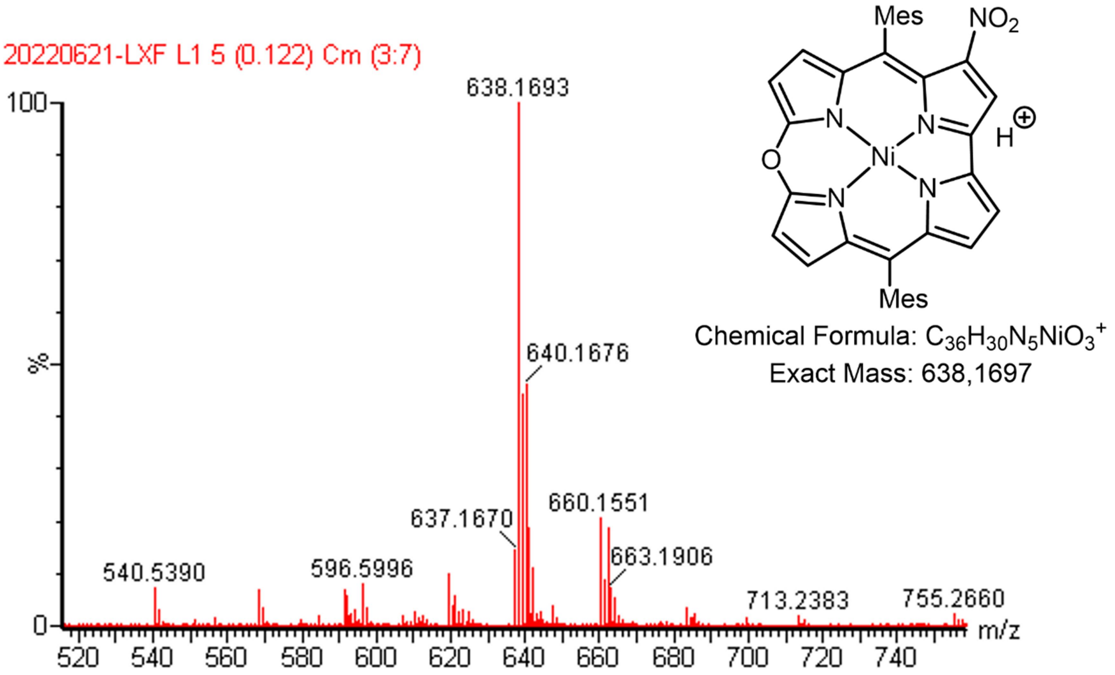

Figure S41. API-HRMS spectrum of **2-NO$_2$**.

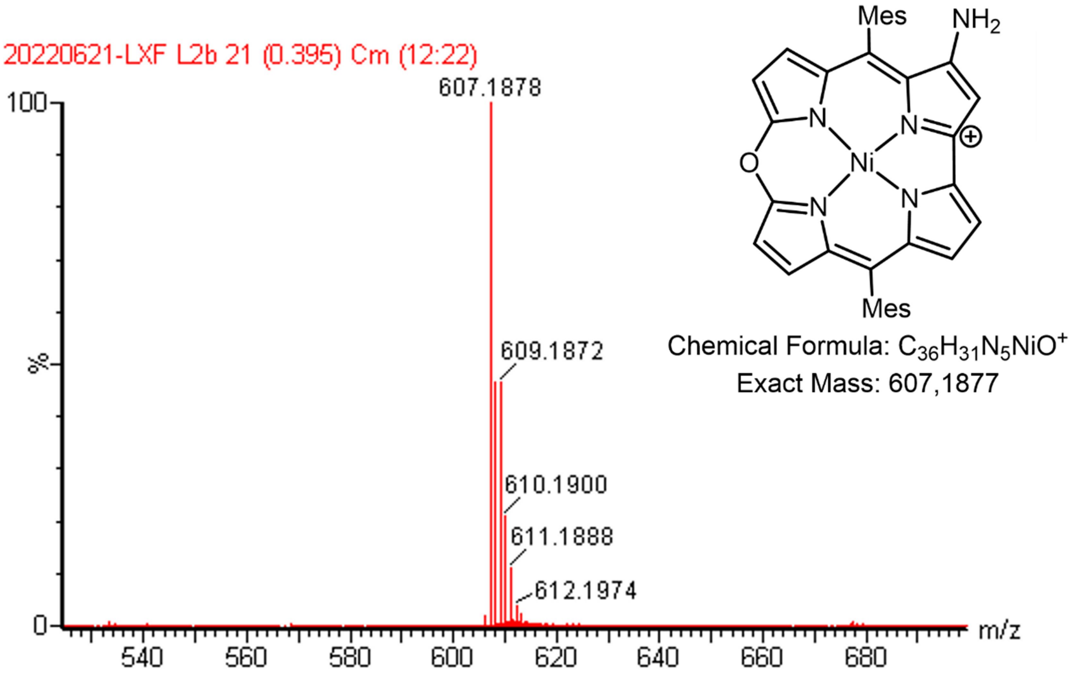

Figure S42. API-HRMS spectrum of **2-NH$_2$**.

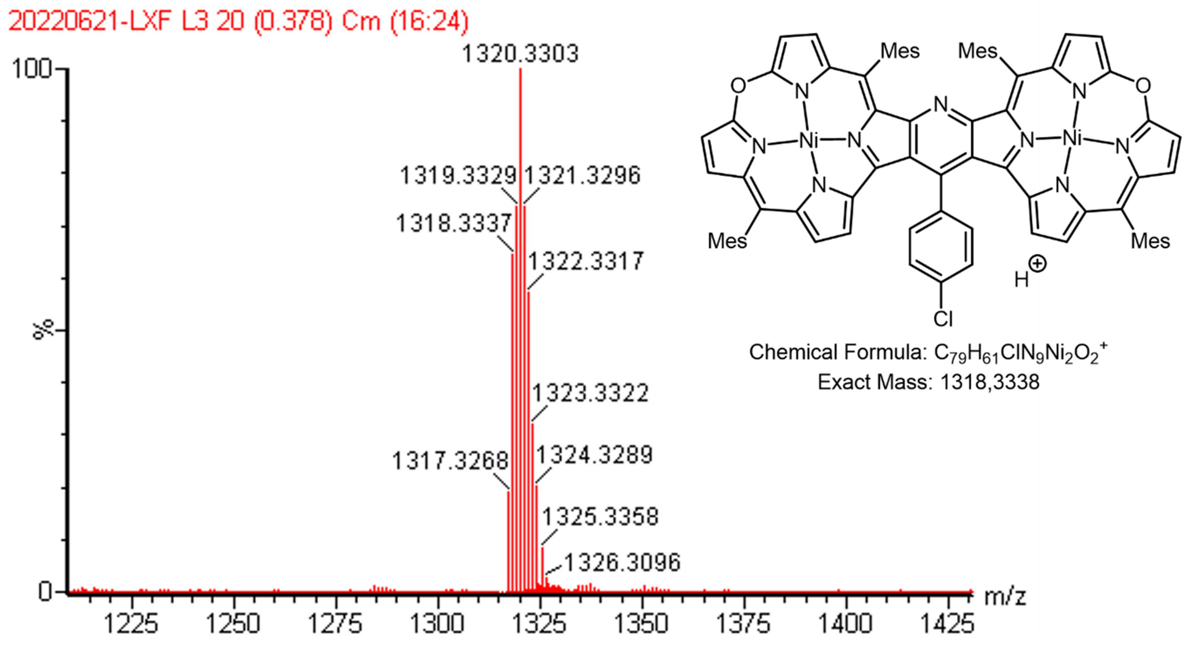

Figure S43. API-HRMS spectrum of **3a**.

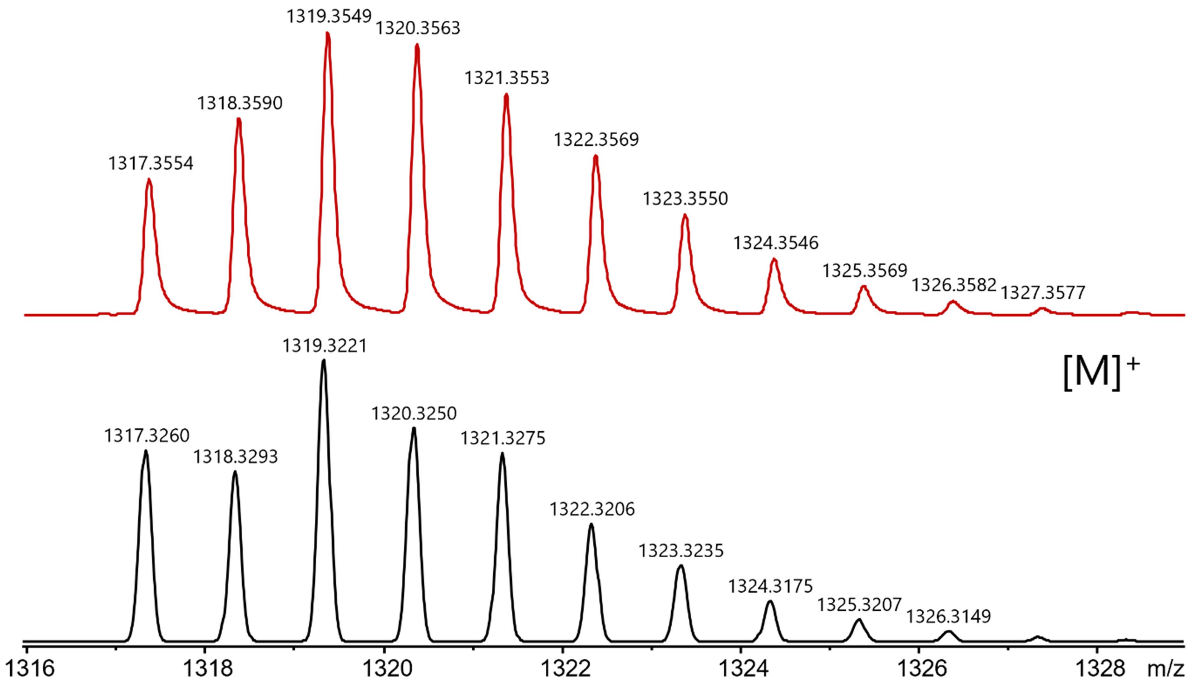

Figure S44. ESI-HRMS spectrum of **3a** (experimental: red, upper trace; simulated: black, bottom trace).

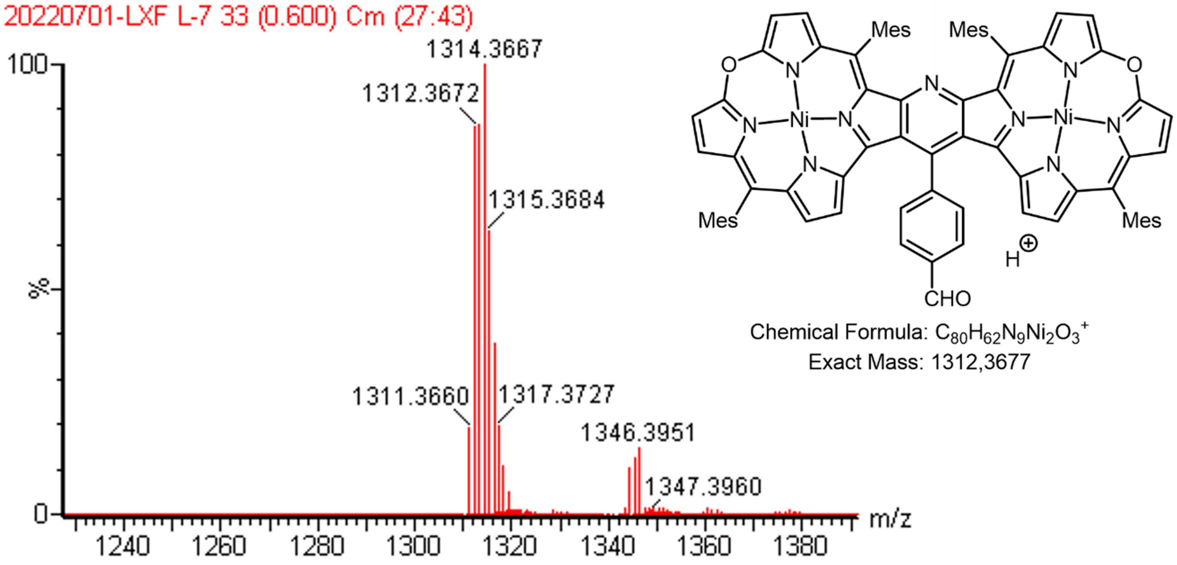

Figure S45. API-HRMS spectrum of **3b**.

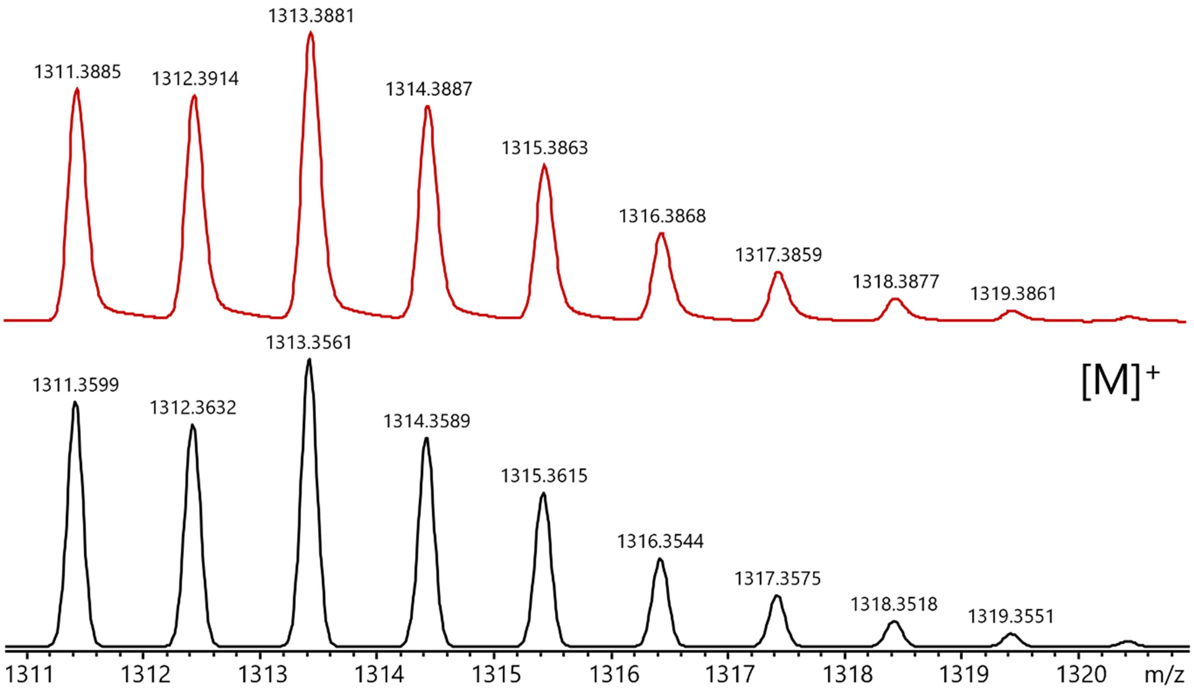

Figure S46. ESI-HRMS spectrum of **3b** (experimental: red, upper trace; simulated: black, bottom trace).

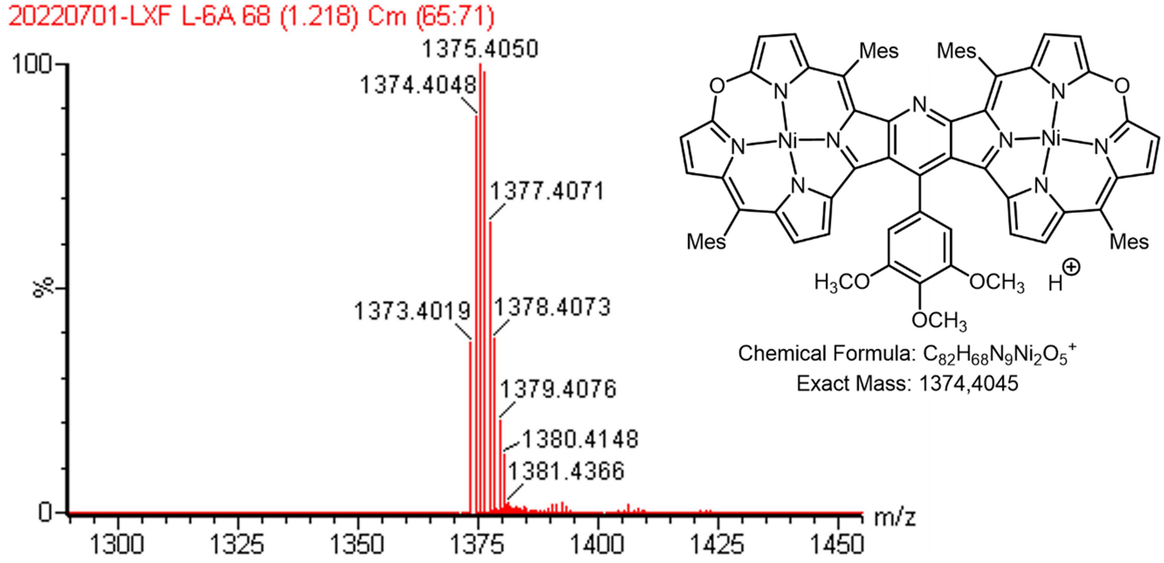

Figure S47. API-HRMS spectrum of **3c**.

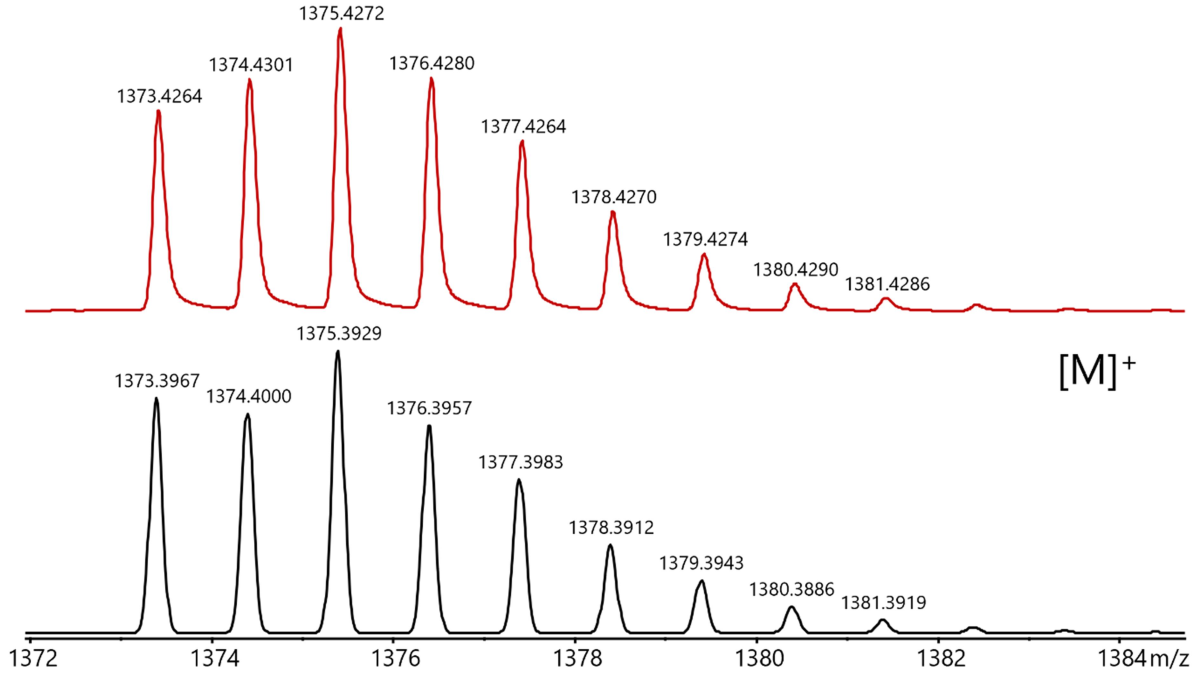

Figure S48. ESI-HRMS spectrum of **3c** (experimental: red, upper trace; simulated: black, bottom trace).

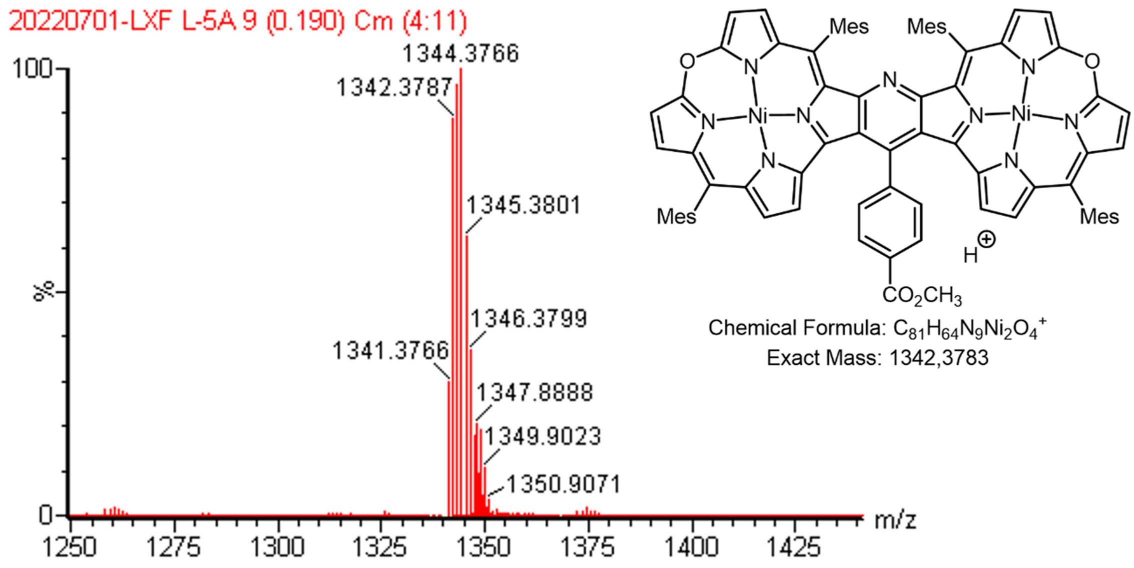

Figure S49. API-HRMS spectrum of **3d**.

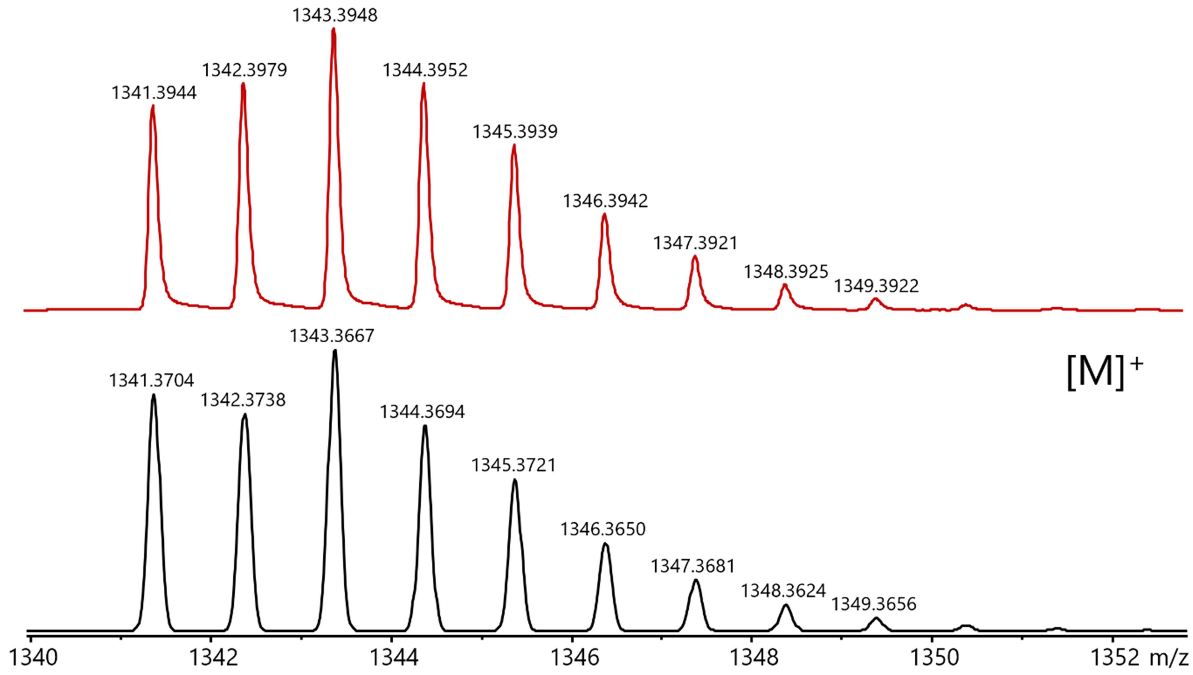

Figure S50. ESI-HRMS spectrum of **3d** (experimental: red, upper trace; simulated: black, bottom trace).

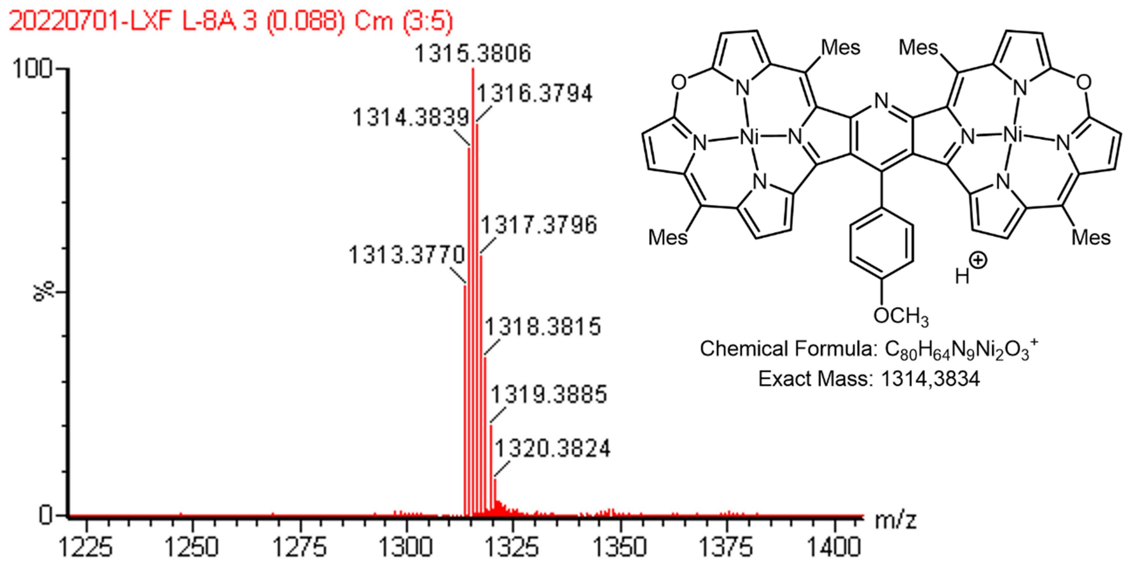

Figure S51. API-HRMS spectrum of **3e**.

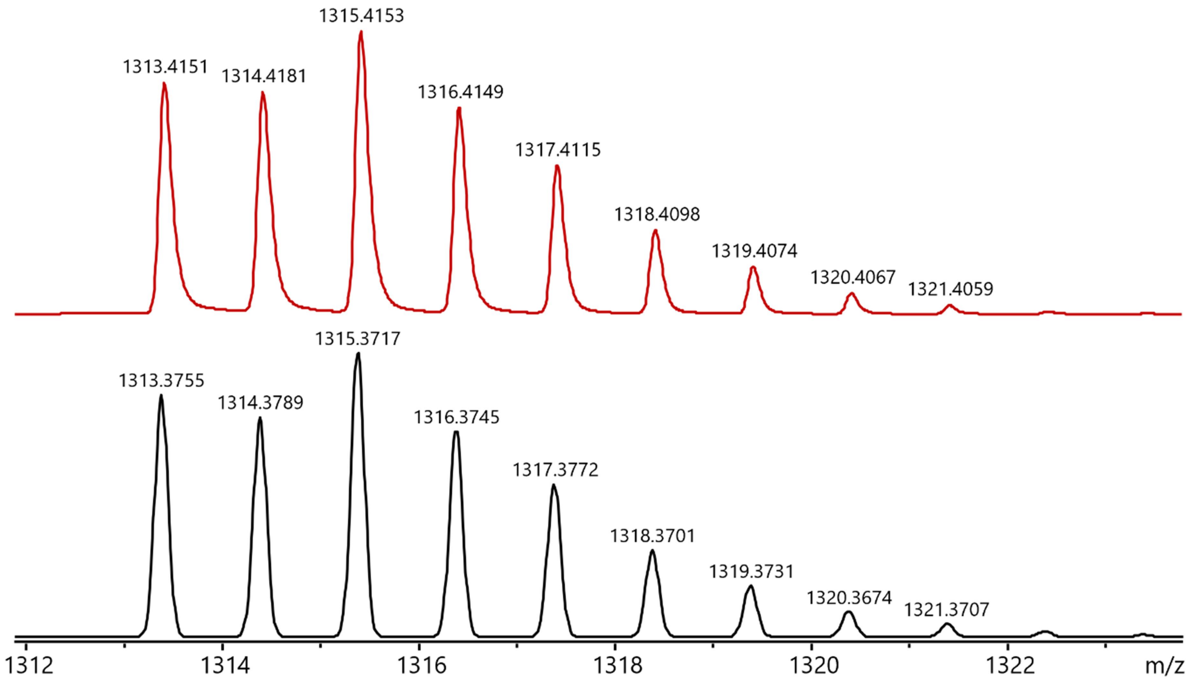

Figure S52. ESI-HRMS spectrum of **3e** (experimental: red, upper trace; simulated: black, bottom trace).

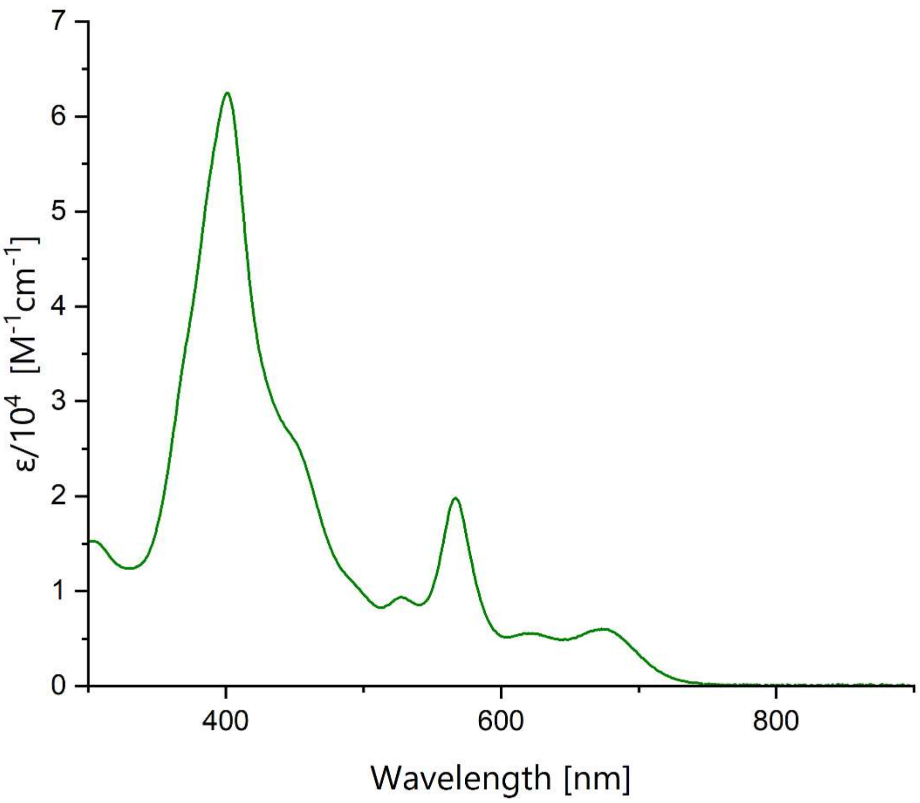

Figure S53. UV-Vis spectrum of **2-NO$_2$** in dichloromethane.

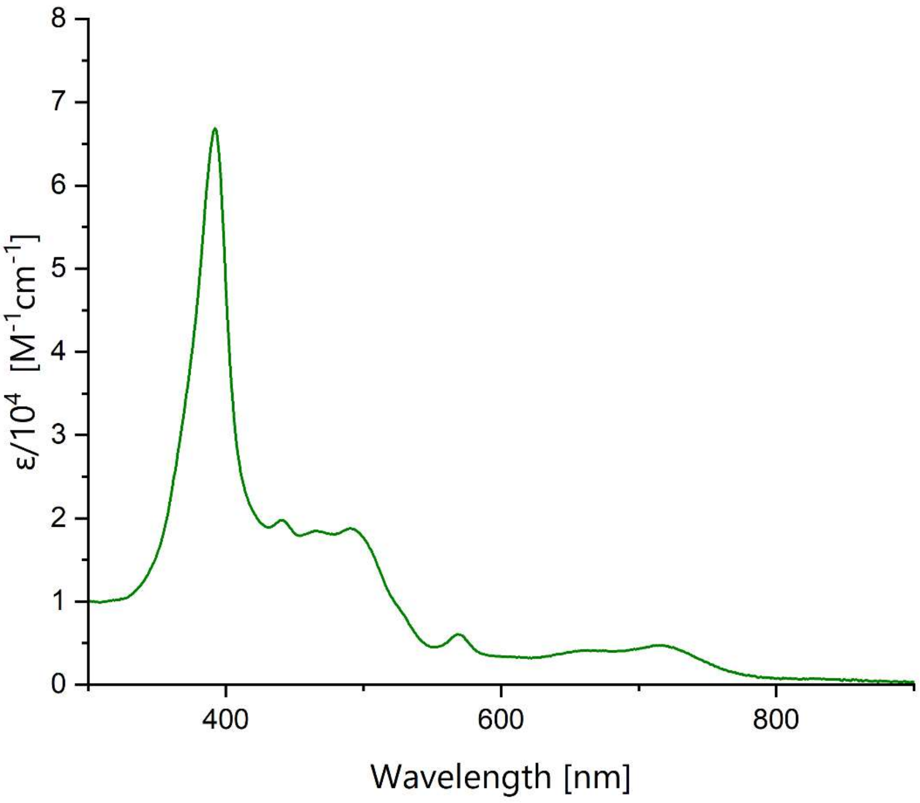

Figure S54. UV-Vis spectrum of **2-NH$_2$** in dichloromethane.

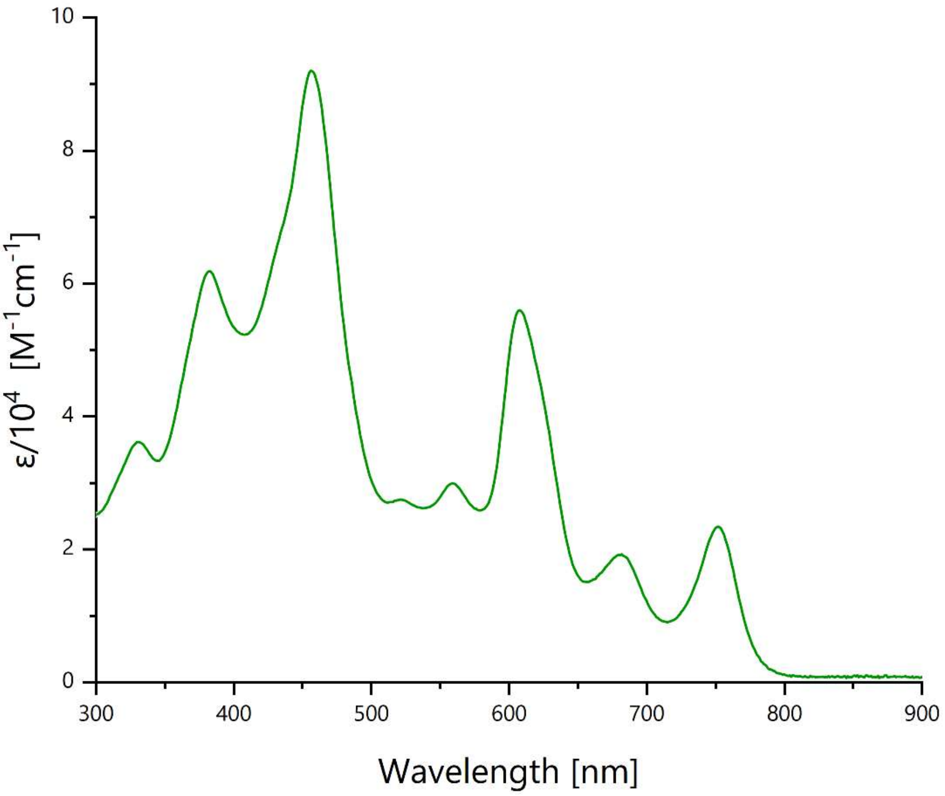

Figure S55. UV-Vis spectrum of **3a** in dichloromethane.

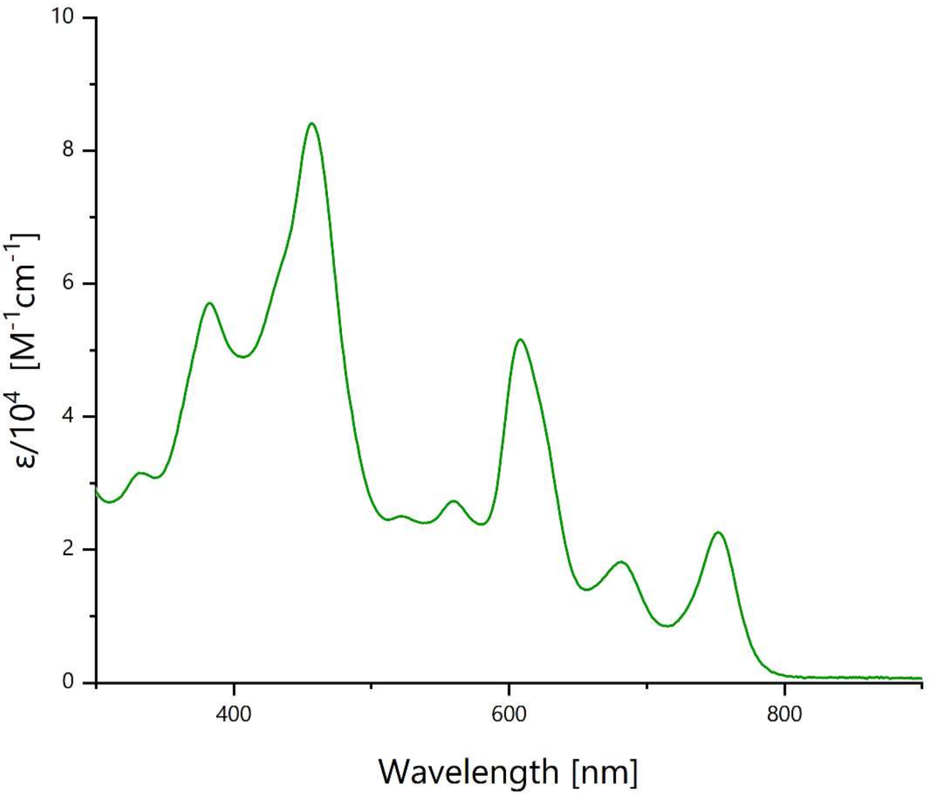

Figure S56. UV-Vis spectrum of **3b** in dichloromethane.

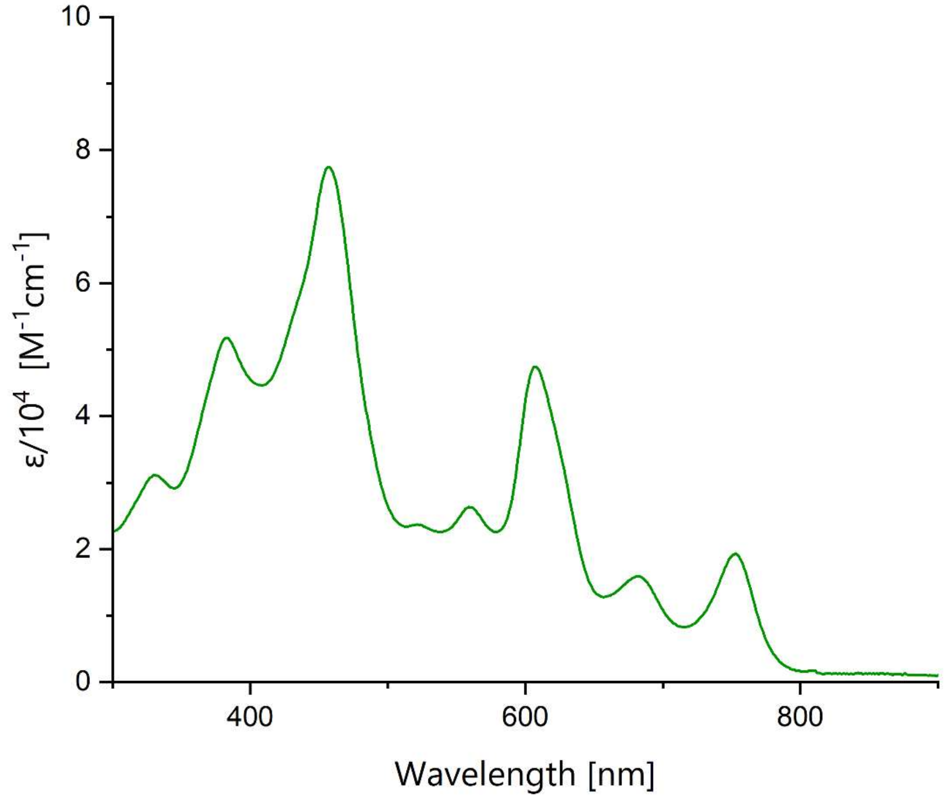

Figure S57. UV-Vis spectrum of **3c** in dichloromethane.

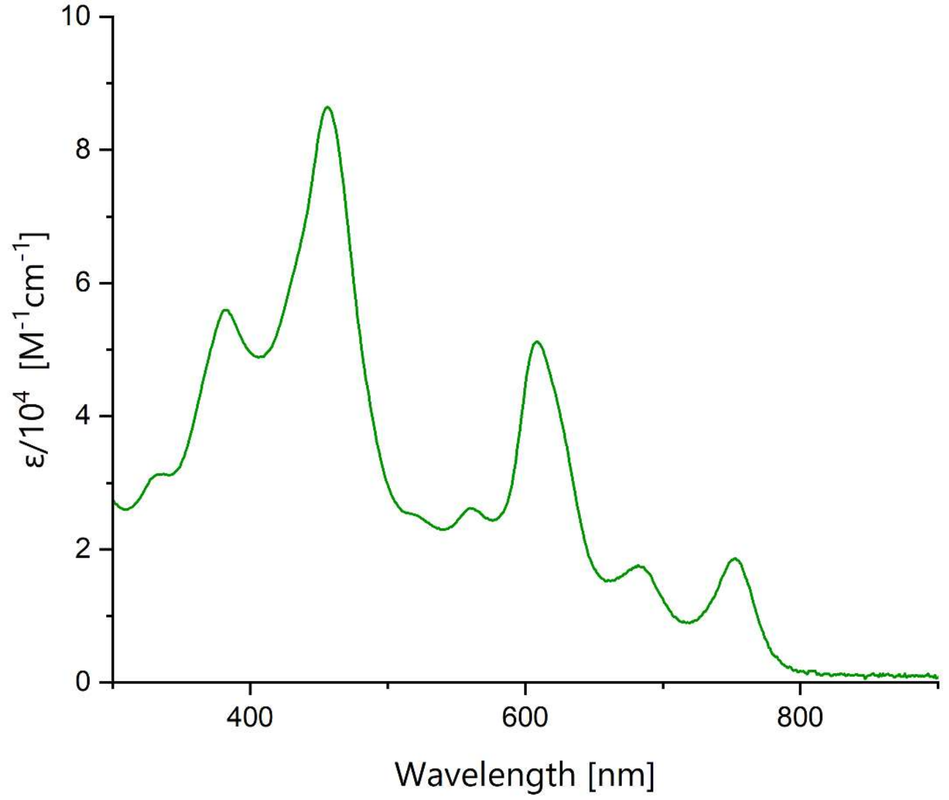

Figure S58. UV-Vis spectrum of **3d** in dichloromethane.

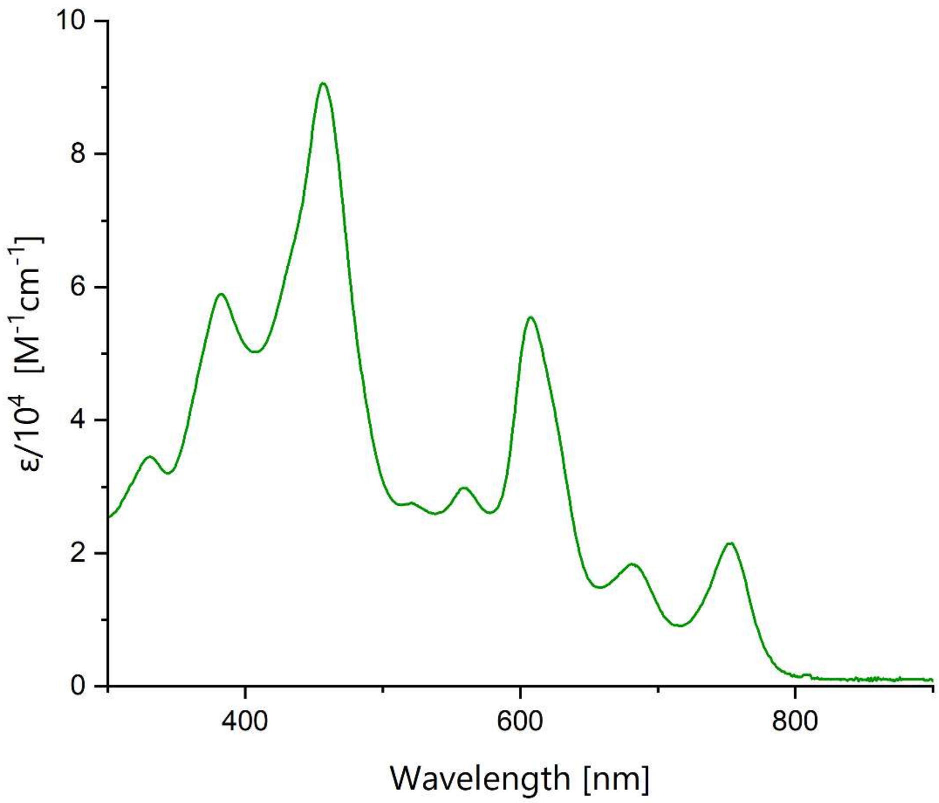

Figure S59. UV-Vis spectrum of **3e** in dichloromethane.

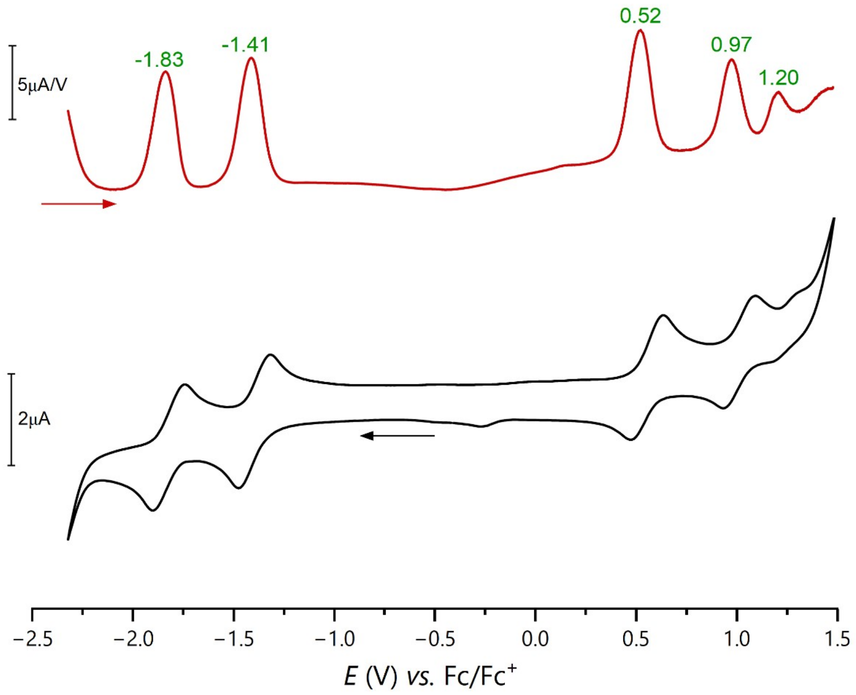

Figure S60. Differential pulse (DP) and cyclic (CV) voltammograms recorded for **2-NO$_2$** in DCM with $[Bu_4N]PF_6$ as supporting electrolyte.

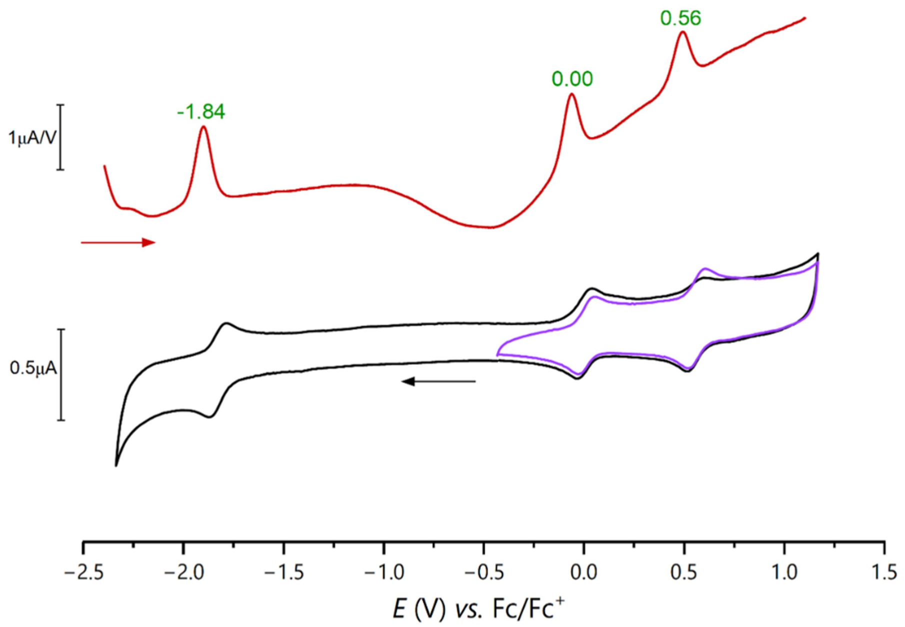

Figure S61. Differential pulse (DP) and cyclic (CV) voltammograms recorded for **2-NH$_2$** in DCM with $[Bu_4N]PF_6$ as supporting electrolyte.

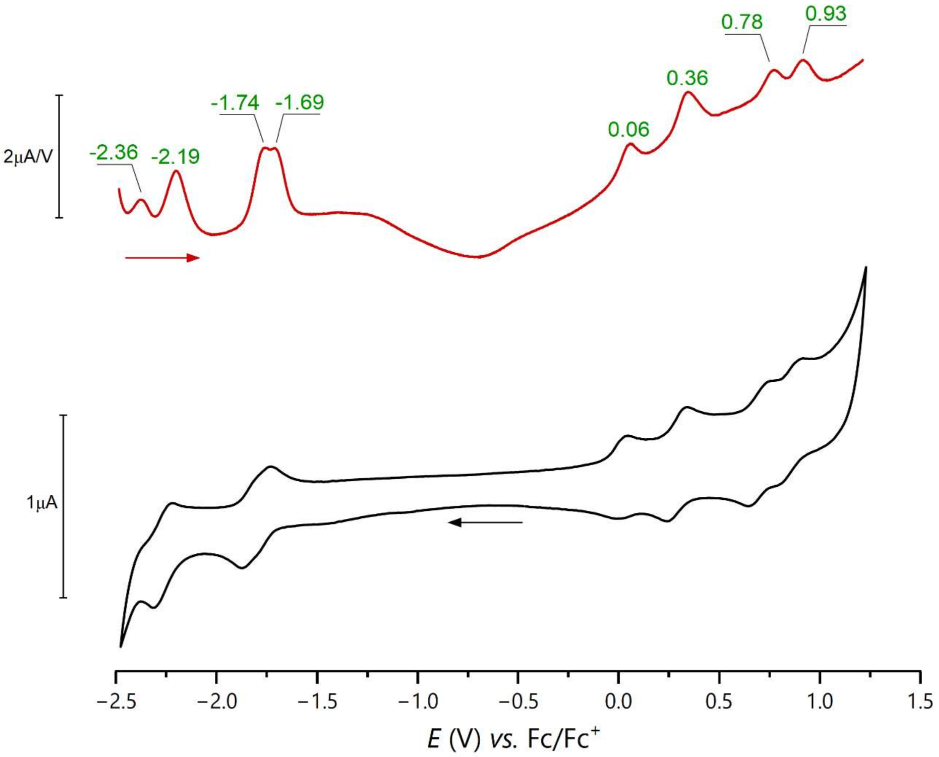

Figure S62. Differential pulse (DP) and cyclic (CV) voltammograms recorded for **3a** in DCM with $[Bu_4N]PF_6$ as supporting electrolyte.

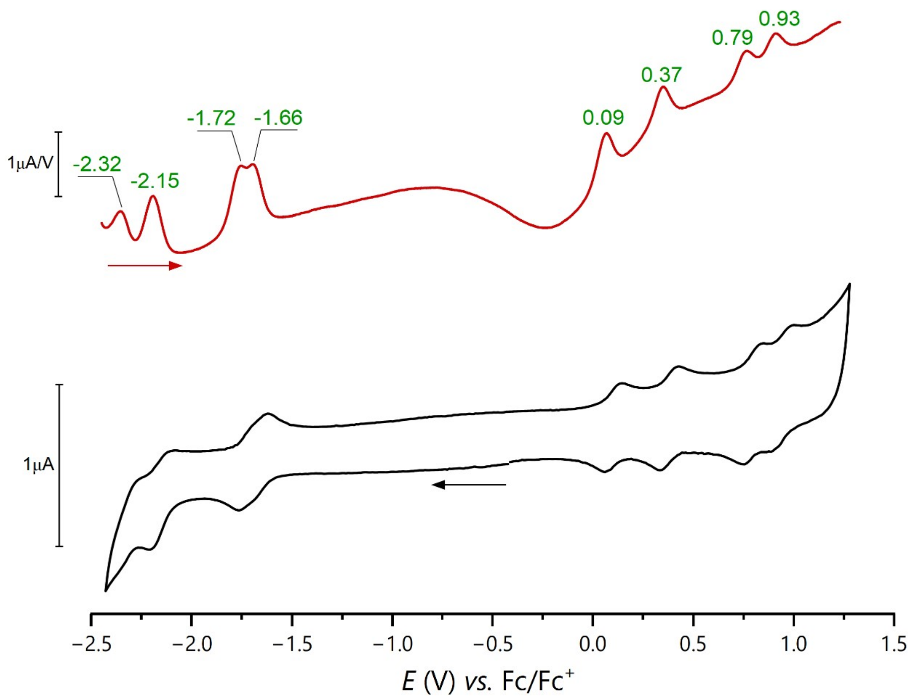

Figure S63. Differential pulse (DP) and cyclic (CV) voltammograms recorded for **3b** in DCM with $[Bu_4N]PF_6$ as supporting electrolyte.

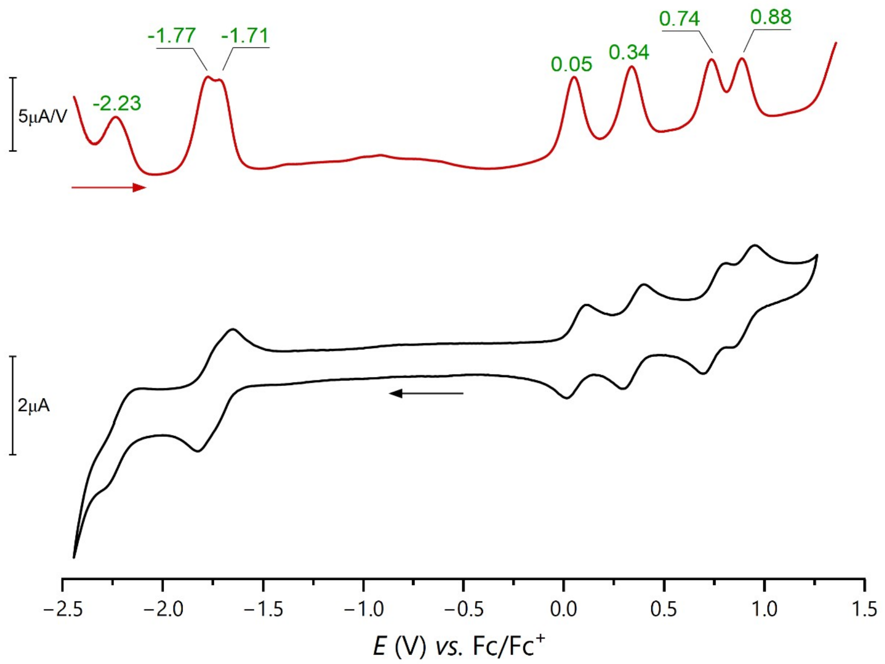

Figure S64. Differential pulse (DP) and cyclic (CV) voltammograms recorded for **3c** in DCM with $[Bu_4N]PF_6$ as supporting electrolyte.

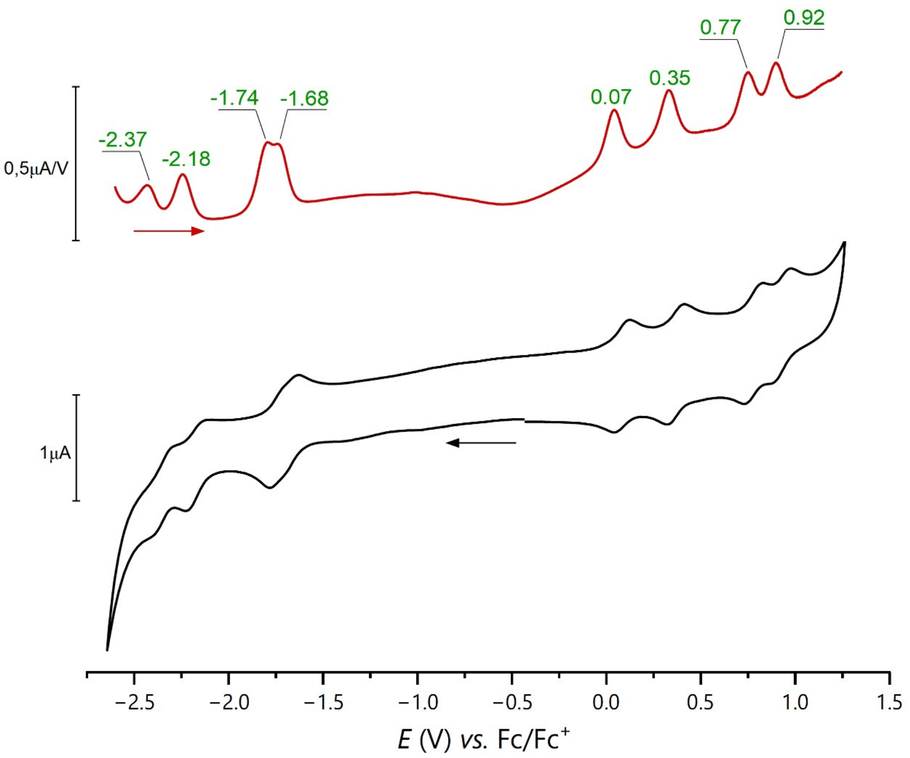

Figure S65. Differential pulse (DP) and cyclic (CV) voltammograms recorded for **3d** in DCM with $[Bu_4N]PF_6$ as supporting electrolyte.

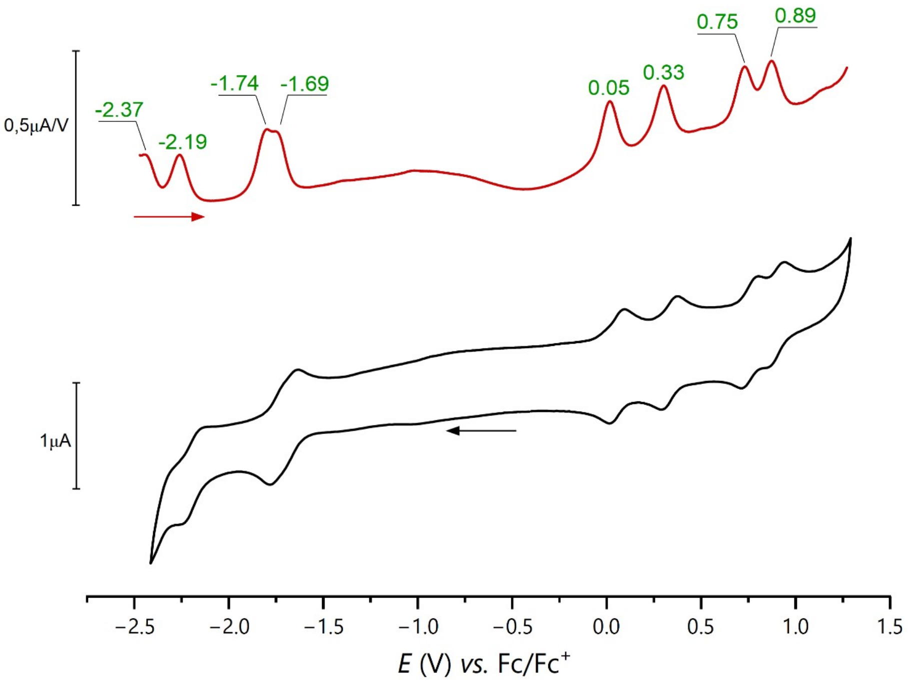

Figure S66. Differential pulse (DP) and cyclic (CV) voltammograms recorded for **3e** in DCM with $[Bu_4N]PF_6$ as supporting electrolyte.

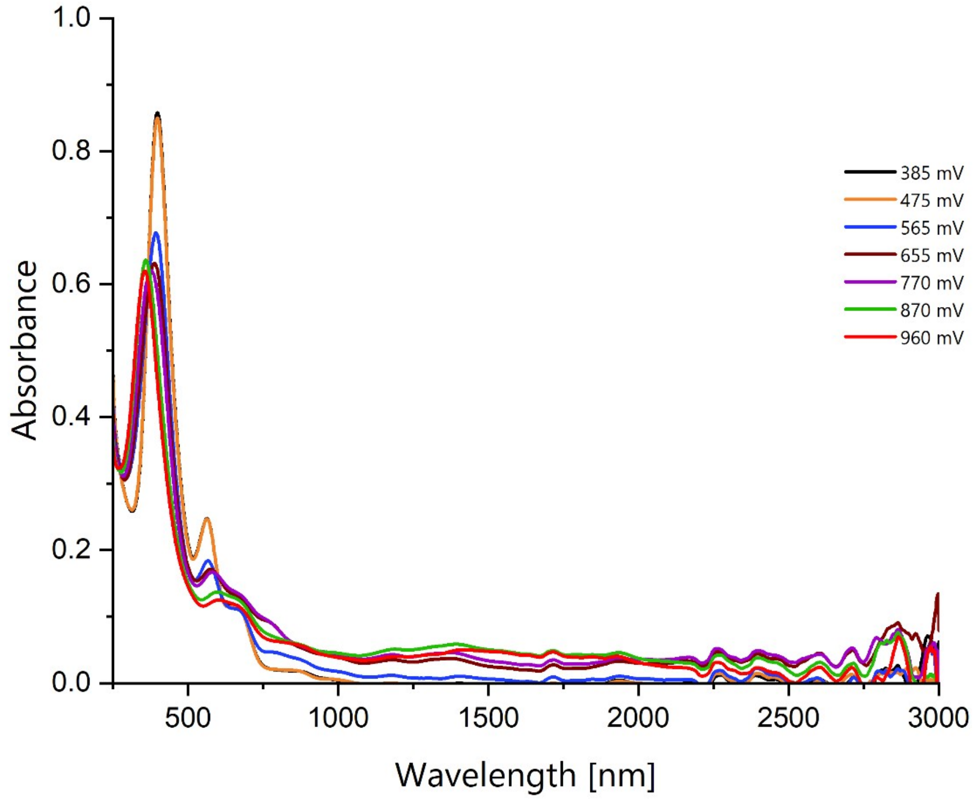

Figure S67. UV-Vis-NIR spectra recorded upon electrochemical oxidation of **2-NO$_2$** (0.1 M $[Bu_4N]PF_6$, DCM).

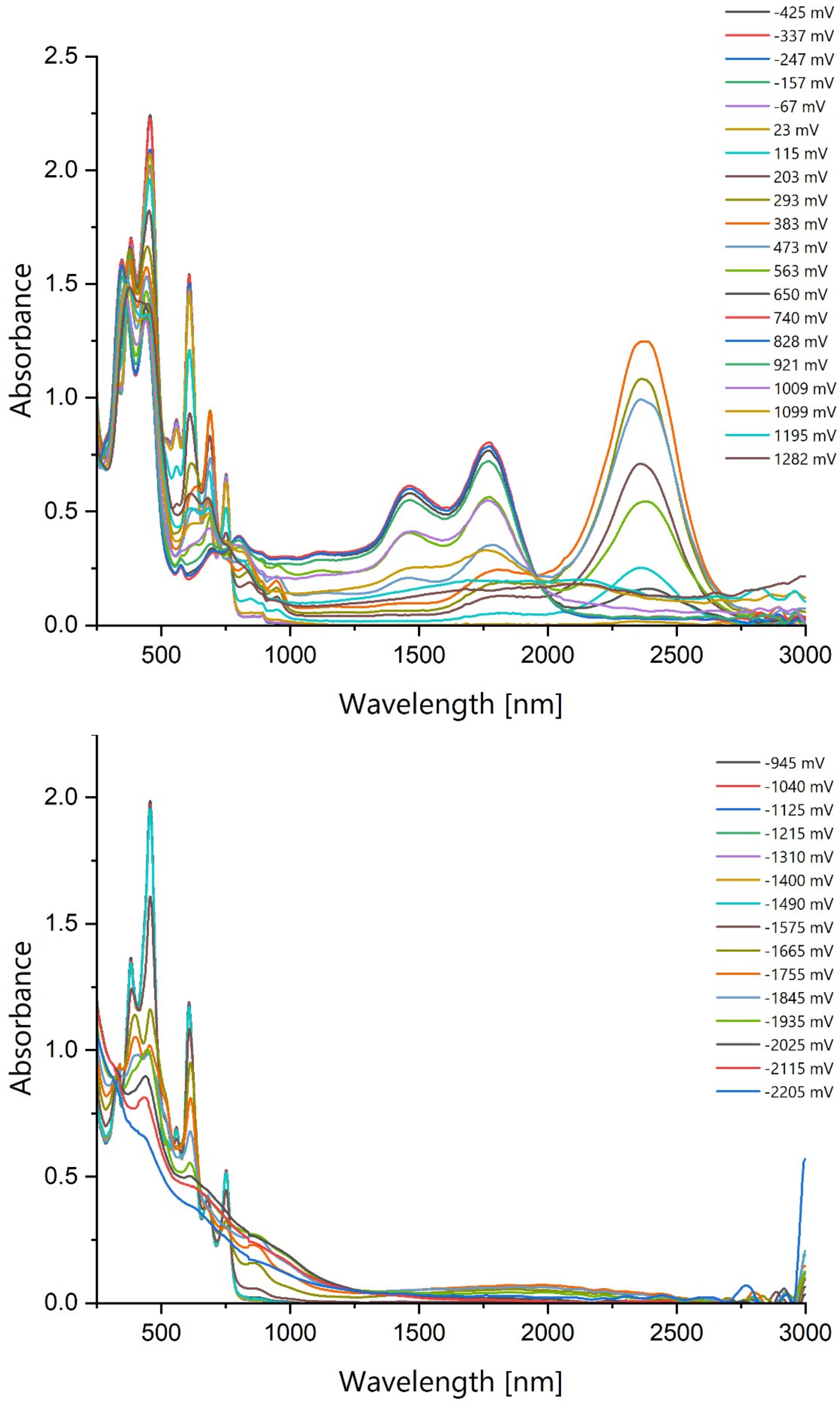

Figure S68. UV-Vis-NIR spectra recorded upon electrochemical oxidation of **3c** (0.1 M $[Bu_4N]PF_6$, DCM). Upper panel presents spectral changes during anodic scan and the bottom panel shows spectral changes during cathodic scan.

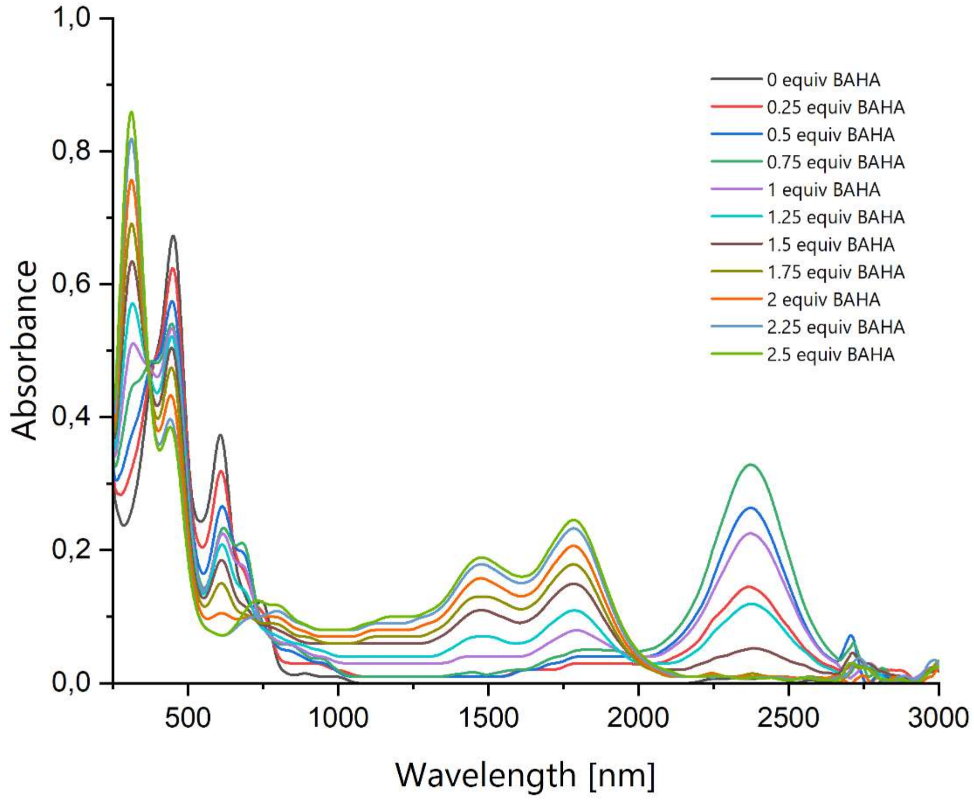

Figure S69. UV-Vis-NIR spectra recorded upon addition of tris(4-bromophenyl)ammoniumyl hexachloroantimonate (BAHA) to the DCM solution of **3c** with the amount of the added oxidant specified in the legend.

Table S1. Crystal data for **2-NO$_2$**, **3a** and **3c**.

	**2-NO$_2$**	**3a**	**3c**
**Chemical formula**	$C_{36}H_{29}N_5NiO_3$	$2(C_{79}H_{60}ClN_9Ni_2O_2)\cdot C_6H_{14}$	$C_{82}H_{67}N_9Ni_2O_5\cdot CH_3CN$
**M$_r$**	638.35	2726.62	1416.92
**Temperature [K]**	170	170	100
**Crystal system, space group**	Monoclinic, $P2_1$	Triclinic, $P\bar{1}$	Monoclinic, $P2_1/n$
**a [Å]** **b [Å]** **c [Å]**	8.1919(7) 13.5603(8) 13.6763(8)	15.1399(9), 15.7071(8), 36.6504(10)	20.4671(4), 11.3067(2), 30.4914(5)
**α [°]** **β [°]** **γ [°]**	90 104.640(7) 90	78.424(3) 85.745(3) 64.332(6)	90 103.722(2) 90
**Volume [Å^3]**	1469.90(18)	7695.0(7)	6854.8(2)
**Z**	2	2	4
**Radiation type**	Mo Kα	Mo Kα	Cu Kα
**μ [mm^{-1}]**	0.707	0.574	1.19
**Crystal size**	0.12 × 0.1 × 0.08	0.32 × 0.14 × 0.03	0.35 × 0.06 × 0.02
**Data collection**			
**Diffractometer**	XtaLAB AFC10 (RCD3): fixed-chi	XtaLAB AFC10 (RCD3): fixed-chi	XtaLAB Synergy R, DW system, HyPix-Arc 150
**Absorption correction**	Multi-scan, *CrysAlisPro* 1.171.39.45i (Rigaku Oxford Diffraction, 2018) Empirical absorption correction using spherical harmonics, implemented in SCALE3 ABSPACK scaling algorithm.	Multi-scan, *CrysAlisPro* 1.171.39.45i (Rigaku Oxford Diffraction, 2018) Empirical absorption correction using spherical harmonics, implemented in SCALE3 ABSPACK scaling algorithm.	Multi-scan *CrysAlisPRO* 1.171. 43.105a (Rigaku Oxford Diffraction, 2024) Empirical absorption correction using spherical harmonics, implemented in SCALE3 ABSPACK scaling algorithm.
**T_{min}, T_{max}**	0.647, 1.000	0.357, 1.000	0.711, 1.000
**No. of measured, independent and observed [$I > 2\sigma(I)$] reflections**	23632, 7680, 6252	116074, 40477, 21850	58939, 13252, 8326
**R_{int}**	0.073	0.131	0.040
**(sin θ/λ)max [Å^{-1}]**	0.726	0.720	0.621
**Refinement**			
**$R[F^2>2\sigma(F^2)]$, $wR(F^2)$, S**	0.084, 0.220, 1.04	0.096, 0.286, 1.00	0.055, 0.163, 1.03
**No. of reflections**	7680	40477	13252
**No. of parameters**	413	1971	926
**No. of restraints**	1	1056	1
**H-atom treatment**	H-atom parameters constrained	H-atom parameters constrained	H-atom parameters constrained
**$\Delta\rho_{max}$, $\Delta\rho_{min}$ [e Å^{-3}]**	2.25, −0.67	2.38, −1.02	0.64, −0.36
**CCDC number**	2431678	2431677	2431674

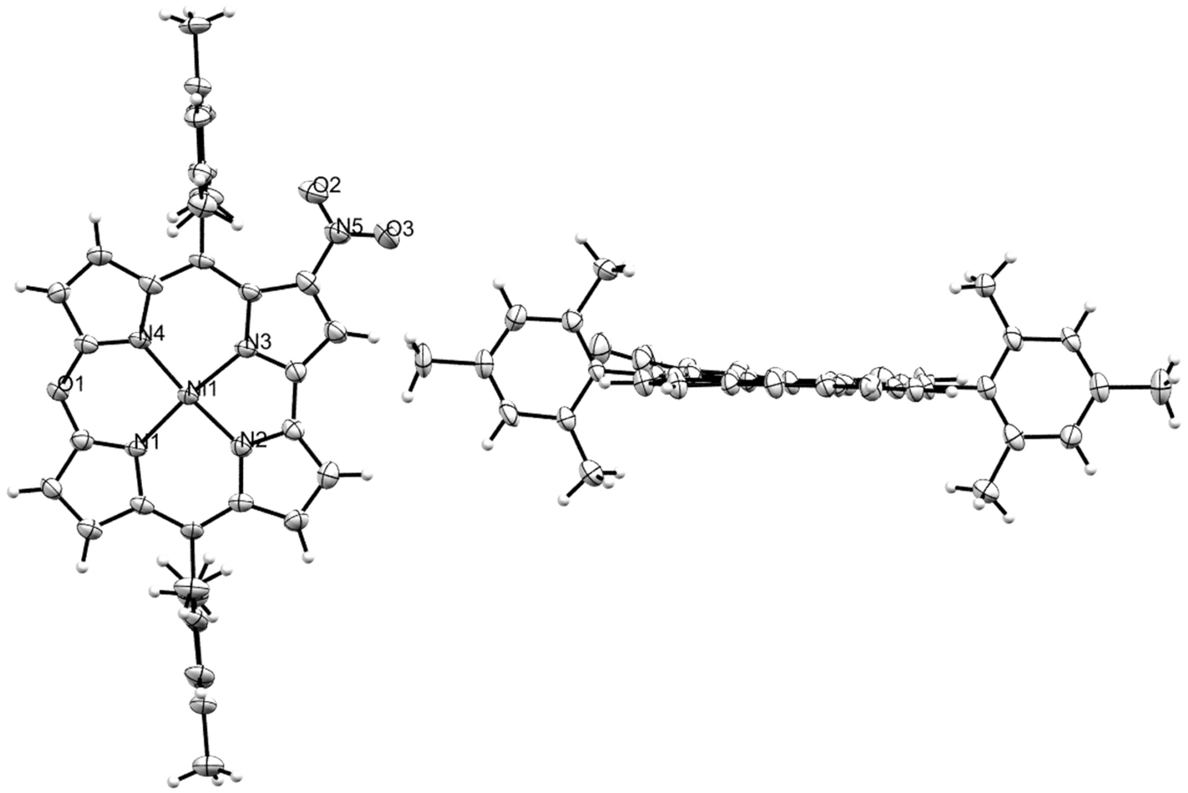

Figure S70. Two views of the ORTEP representations (ellipsoids set on the 50% probability level) of the asymmetric unit of the **2-NO$_2$** crystal structure.

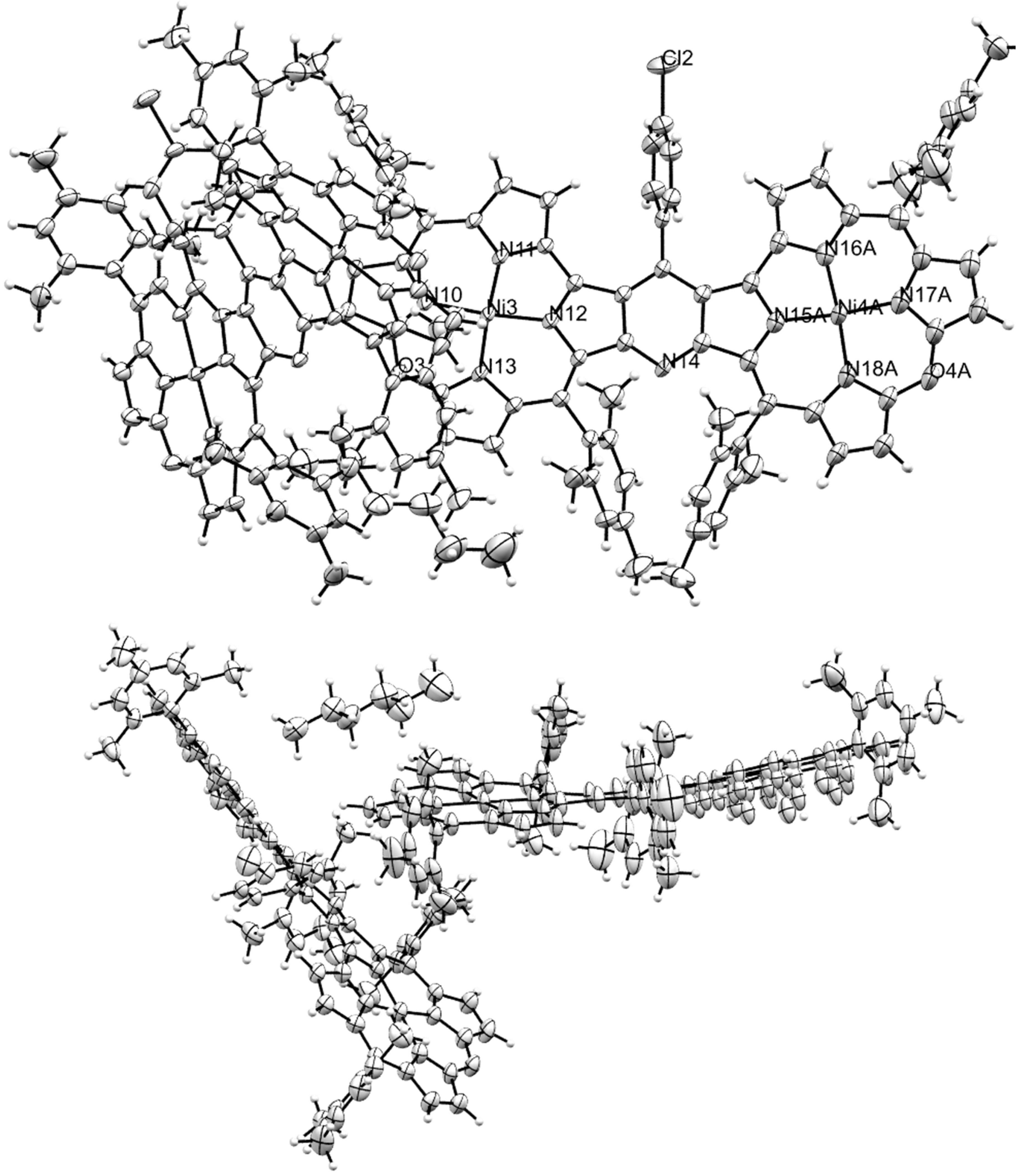

Figure S71. Two views of the ORTEP representations (ellipsoids set on the 50% probability level) of the asymmetric unit of the **3a** crystal structure.

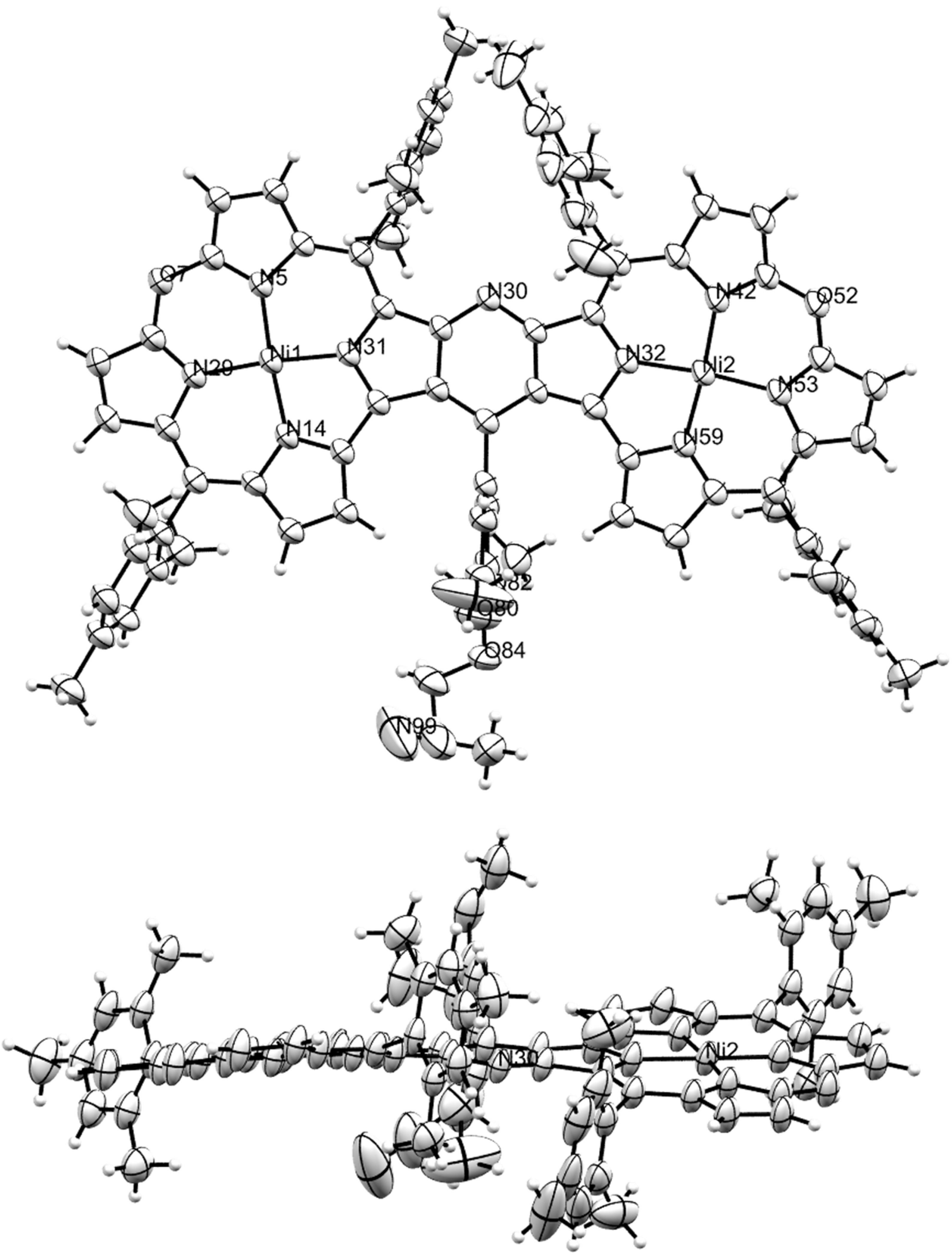

Figure S72. Two views of the ORTEP representations (ellipsoids set on the 50% probability level) of the asymmetric unit of the **3c** crystal structure.

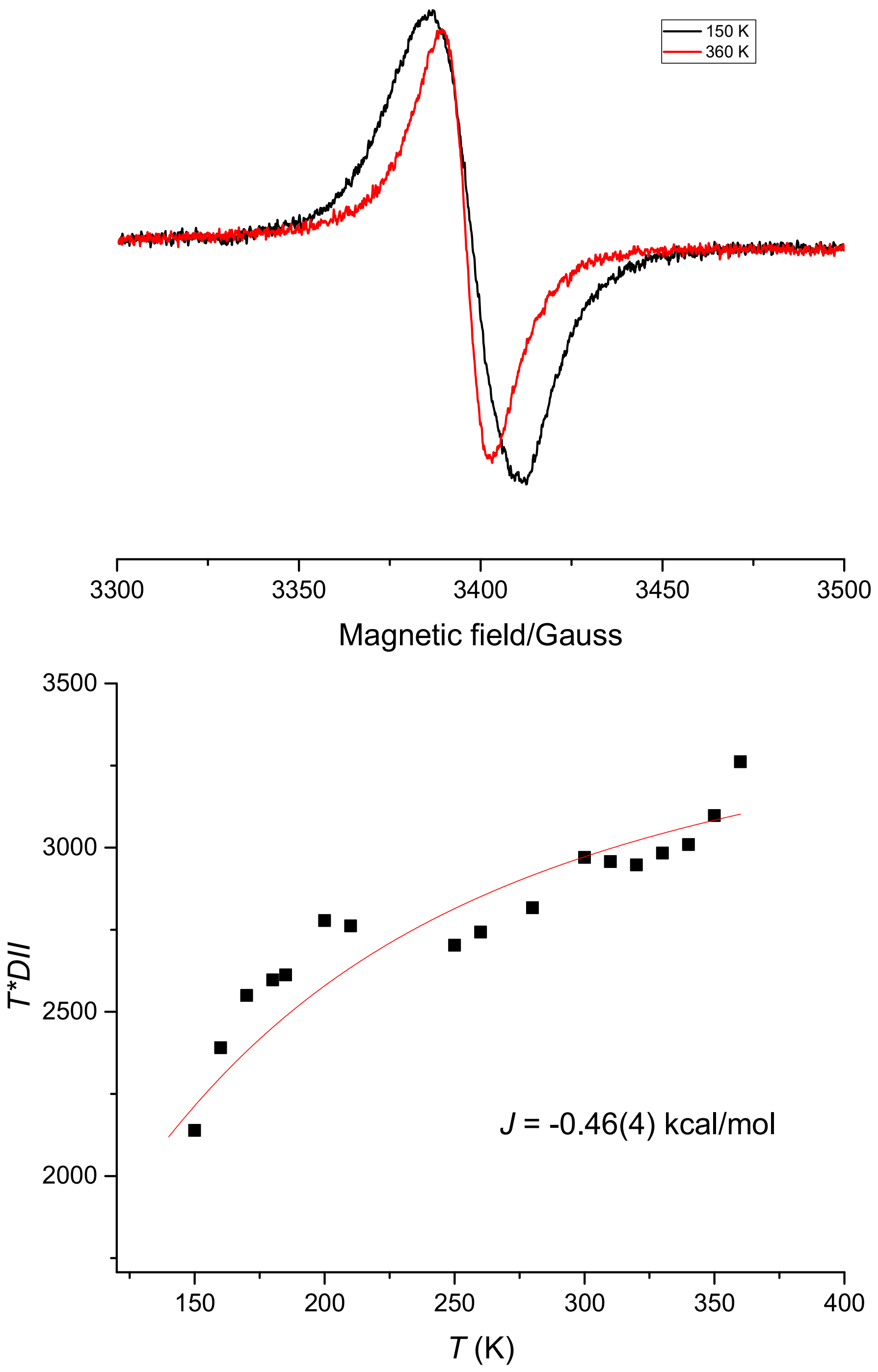

Figure S73. Top: solid state ESR spectra of [**3c**]$^{2+}$ at specified temperatures. Bottom: temperature dependence of the product of temperature and doubly integrated intensity of the ESR signals from the spectra taken upon the variable-temperature ESR experiment for the solid sample of [3c]$^{2+}$. The black squares represent experimental data, and the red line is the best fit curve calculated on the basis of the data fitting to the Bleaney-Bowers equation with the exchange integral J = –0.46 kcal/mol.

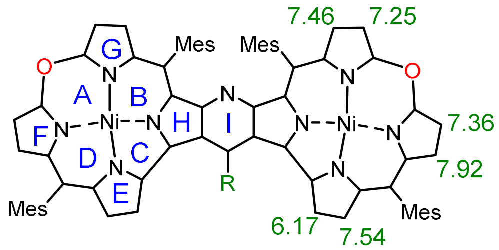

Ring	NICS(–1)	NICS(0)	NICS(1)
A	–11.1	–14.8	–10.9
B	–7.8	–8.8	–7.2
C	–6.3	–8.8	–7.2
D	–8.0	–9.4	–8.0
E	–8.7	–7.1	–8.0
F	–7.2	–6.0	–7.2
G	–8.8	–8.0	–8.0
H	–6.0	–3.0	–6.0
I	–9.2	–6.8	–9.2

Figure S74. GIAO calculated NICS(x) values (x = –1, 0, 1) and calculated pyrrole proton chemical shifts (green numbers; in ppm) for **3c**. The NICS(x) were estimated x Å over a mean plane of the macrocycle in the midpoint of each ring specified with the blue letter.

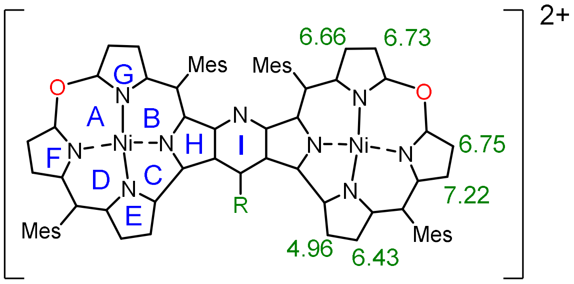

Ring	NICS(–1)	NICS(0)	NICS(1)
A	–1.7	–2.6	–1.8
B	0.5	0.8	0.6
C	3.1	4.0	3.2
D	0.8	1.2	0.9
E	–7.5	–6.0	–7.7
F	–5.8	–4.0	–5.7
G	–5.1	–4.5	–4.6
H	–1.7	2.1	–1.8
I	–4.6	–1.5	–4.6

Figure S75. GIAO calculated NICS(x) values (x = –1, 0, 1) and calculated pyrrole proton chemical shifts (green numbers; in ppm) for [**3c**]$^{2+}$. The NICS(x) were estimated x Å over a mean plane of the macrocycle in the midpoint of each ring specified with the blue letter.

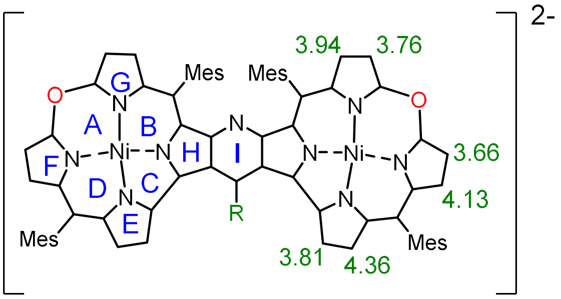

Ring	NICS(–1)	NICS(0)	NICS(1)
A	9.7	10.7	8.6
B	3.8	4.3	3.3
C	2.8	–0.2	2.2
D	4.2	5.2	5.4
E	–3.3	–1.4	–2.3
F	–5.1	–5.5	–4.3
G	–7.4	–9.8	–9.3
H	–4.3	–2.2	–5.1
I	5.6	12.3	5.7

Figure S76. GIAO calculated NICS(x) values (x = –1, 0, 1) and calculated pyrrole proton chemical shifts (green numbers; in ppm) for [**3c**]$^{2-}$. The NICS(x) were estimated x Å over a mean plane of the macrocycle in the midpoint of each ring specified with the blue letter.

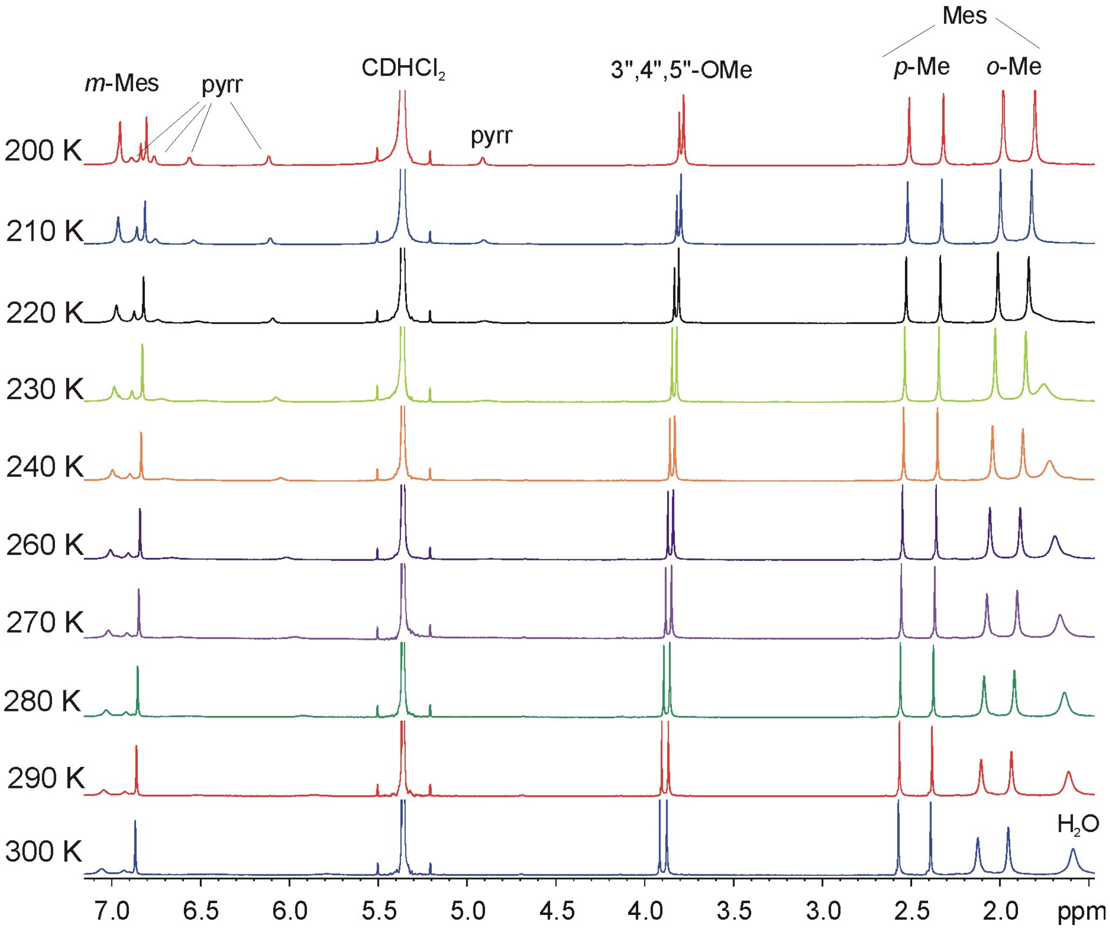

Figure S77. Variable temperature ^{1}H NMR (600 MHz) experiment for [**3c**]$^{2+}$ in CD_2Cl_2.

Table S2. TD DFT calculated electronic transitions for **3c** (*in vacuo*).

No	Energy (cm^{-1})	Wavelength (nm)	Osc. Strength	Symmetry	Major contributions
1	14818.0	674.9	0.0915	Singlet-A	HOMO->LUMO (92%)
2	15834.3	631.5	0.0077	Singlet-A	HOMO->L+1 (82%)
3	17716.0	564.5	0.1351	Singlet-A	H-1->LUMO (20%), HOMO->L+2 (69%)
4	17958.7	556.8	0.3204	Singlet-A	H-1->LUMO (61%), HOMO->L+1 (10%), HOMO->L+2 (20%)
5	18509.6	540.3	0.0162	Singlet-A	H-1->L+1 (75%)
6	20301.0	492.6	0.0034	Singlet-A	H-6->L+6 (11%), H-5->L+5 (13%), H-1->L+6 (10%), HOMO->L+5 (17%)
7	20328.4	491.9	0.0019	Singlet-A	H-6->L+5 (12%), H-4->L+6 (12%), H-1->L+5 (11%), HOMO->L+6 (18%)
8	20384.9	490.6	0.0019	Singlet-A	H-2->LUMO (27%), H-1->L+2 (18%), HOMO->L+3 (30%)
9	20590.5	485.7	0.0152	Singlet-A	H-5->LUMO (13%), H-4->LUMO (11%), H-4->L+1 (11%), H-3->LUMO (35%)
10	20671.2	483.8	2E-4	Singlet-A	H-5->LUMO (21%), H-4->L+1 (14%), H-3->L+1 (12%), H-2->LUMO (12%)
11	21439.0	466.4	0.0047	Singlet-A	H-12->L+5 (11%), H-6->LUMO (17%), H-2->L+1 (25%)
12	21611.6	462.7	0.0088	Singlet-A	H-1->L+2 (42%), HOMO->L+3 (37%)
13	21657.6	461.7	0.0237	Singlet-A	H-10->L+6 (12%), HOMO->L+3 (12%)
14	21935.1	455.9	0.0205	Singlet-A	H-12->LUMO (22%), H-2->LUMO (15%), H-2->L+1 (11%)
15	22041.5	453.7	0.0225	Singlet-A	H-10->LUMO (11%), H-10->L+1 (13%), H-4->L+6 (12%), H-3->L+1 (11%)
16	22093.9	452.6	0.0097	Singlet-A	H-5->L+5 (29%)
17	22114.9	452.2	0.0345	Singlet-A	H-6->L+6 (10%), H-4->L+6 (17%)
18	22234.3	449.8	0.0054	Singlet-A	H-12->LUMO (23%)
19	22483.5	444.8	0.024	Singlet-A	H-3->LUMO (26%), H-2->L+1 (23%)
20	22814.2	438.3	0.404	Singlet-A	H-12->L+5 (10%), H-3->LUMO (16%)
21	23173.1	431.5	0.0376	Singlet-A	H-12->L+5 (10%), H-3->L+1 (18%), HOMO->L+4 (21%)
22	23400.6	427.3	0.4538	Singlet-A	H-6->LUMO (47%), H-2->L+1 (10%)
23	23803.0	420.1	0.0203	Singlet-A	H-7->LUMO (14%), H-6->L+1 (31%), HOMO->L+4 (10%)
24	23918.4	418.1	0.002	Singlet-A	H-7->L+1 (34%), H-4->LUMO (13%), H-4->L+1 (13%)
25	24023.2	416.3	0.0115	Singlet-A	H-7->L+1 (27%)
26	24162.0	413.9	0.0101	Singlet-A	H-7->LUMO (36%), H-4->LUMO (10%)
27	24255.5	412.3	0.0051	Singlet-A	H-7->LUMO (19%), H-5->L+2 (18%)
28	24348.3	410.7	0.0517	Singlet-A	H-2->L+2 (53%), H-1->L+3 (14%)
29	24589.4	406.7	0.0602	Singlet-A	H-5->LUMO (16%), H-5->L+1 (15%), H-3->L+2 (24%)
30	24799.9	403.2	0.0038	Singlet-A	H-9->L+1 (23%), H-7->LUMO (12%), H-6->L+2 (10%), HOMO->L+4 (22%)
31	24994.3	400.1	0.0199	Singlet-A	H-5->L+1 (11%), H-5->L+2 (15%), H-4->L+2 (16%), H-2->L+2 (15%), H-1->L+3 (13%)
32	25089.5	398.6	0.004	Singlet-A	H-11->L+1 (19%), H-9->LUMO (42%)
33	25264.5	395.8	0.0127	Singlet-A	H-11->LUMO (10%), H-9->L+1 (19%), H-8->LUMO (20%), H-6->L+2 (12%)
34	25390.3	393.9	0.0132	Singlet-A	H-8->LUMO (72%)
35	25811.4	387.4	0.3275	Singlet-A	H-11->L+1 (11%), H-5->L+2 (10%), H-1->L+3 (25%)
36	25864.6	386.6	0.1415	Singlet-A	H-11->LUMO (12%), H-6->L+1 (12%), H-5->L+2 (10%), H-4->L+2 (14%), H-3->L+2 (28%)
37	26100.9	383.1	0.0037	Singlet-A	H-10->LUMO (12%), H-10->L+1 (17%), H-10->L+2 (45%)
38	26133.2	382.7	0.0501	Singlet-A	H-11->LUMO (10%), H-11->L+1 (18%), H-9->LUMO (26%)
39	26218.7	381.4	0.007	Singlet-A	H-12->L+1 (16%), H-12->L+2 (59%), H-12->L+3 (10%)
40	26244.5	381.0	0.0237	Singlet-A	H-13->LUMO (58%), H-8->L+1 (19%)
41	26326.7	379.8	0.0059	Singlet-A	H-11->LUMO (25%), H-11->L+1 (20%), H-9->L+1 (10%), H-8->L+1 (16%)
42	26389.7	378.9	0.0422	Singlet-A	H-13->LUMO (23%), H-8->L+1 (49%)
43	26865.5	372.2	0.0398	Singlet-A	H-14->LUMO (50%), H-6->L+2 (25%)
44	27047.0	369.7	9E-4	Singlet-A	H-16->LUMO (44%), H-16->L+1 (37%)
45	27151.8	368.3	0.0081	Singlet-A	H-14->L+1 (19%), H-13->L+1 (41%)
46	27244.6	367.0	4E-4	Singlet-A	H-17->LUMO (38%)
47	27248.6	367.0	9E-4	Singlet-A	H-17->LUMO (27%), H-14->L+1 (10%), H-10->LUMO (14%), H-10->L+1 (12%)
48	27266.4	366.8	0.0037	Singlet-A	H-14->L+1 (11%), H-13->L+1 (13%), H-1->L+4 (21%)
49	27294.6	366.4	0.0067	Singlet-A	H-14->L+1 (13%), H-13->L+1 (18%), H-1->L+4 (37%)
50	27455.1	364.2	0.0018	Singlet-A	H-14->L+1 (10%), H-7->L+2 (72%)
51	27647.1	361.7	0	Singlet-A	H-12->LUMO (20%), H-12->L+1 (54%), H-12->L+2 (15%)
52	27716.4	360.8	0.0355	Singlet-A	H-13->L+1 (19%), H-2->L+3 (36%)
53	28210.0	354.5	5E-4	Singlet-A	H-15->LUMO (24%), H-15->L+1 (45%)
54	28256.0	353.9	5E-4	Singlet-A	H-33->L+5 (39%), H-33->L+6 (17%), H-31->L+5 (10%)
55	28264.9	353.8	0.0031	Singlet-A	H-32->L+6 (10%), H-31->L+6 (34%), H-30->L+6 (14%)
56	28311.7	353.2	0.0393	Singlet-A	H-18->LUMO (29%), H-18->L+1 (23%), H-8->L+2 (26%)
57	28331.8	353.0	0.0449	Singlet-A	H-18->LUMO (19%), H-18->L+1 (15%), H-8->L+2 (43%)

58	28348.0	352.8	0.0181	Singlet-A	H-3->L+3 (61%)
59	28433.5	351.7	0.0262	Singlet-A	H-15->LUMO (50%), H-15->L+1 (13%)
60	28457.7	351.4	0.1318	Singlet-A	H-15->LUMO (10%), H-2->L+3 (25%)

Table S3. TD DFT calculated electronic transitions for **3c** (PCM, dichloromethane)

No	Energy (cm^{-1})	Wavelength (nm)	Osc. Strength	Symmetry	Major contributions
1	14842.2	673.8	0.1343	Singlet-A	HOMO->LUMO (92%)
2	15744.7	635.1	0.0201	Singlet-A	HOMO->L+1 (83%)
3	17732.9	563.9	0.5841	Singlet-A	HOMO->L+2 (88%)
4	17975.7	556.3	0.0917	Singlet-A	H-1->LUMO (78%), HOMO->L+1 (12%)
5	18448.3	542.1	0.0157	Singlet-A	H-1->L+1 (77%)
6	20246.9	493.9	0.0069	Singlet-A	H-6->L+6 (14%), H-5->L+5 (14%), H-1->L+6 (12%), HOMO->L+5 (18%)
7	20269.5	493.4	0.0025	Singlet-A	H-6->L+5 (15%), H-5->L+6 (11%), H-1->L+5 (13%), HOMO->L+6 (17%)
8	20501.8	487.8	0.0017	Singlet-A	H-2->LUMO (22%), H-1->L+2 (22%), HOMO->L+3 (31%)
9	20555.8	486.5	0.0233	Singlet-A	H-5->L+1 (15%), H-4->LUMO (14%), H-3->LUMO (37%)
10	20609.9	485.2	3E-4	Singlet-A	H-5->LUMO (18%), H-4->L+1 (17%), H-3->L+1 (16%), H-2->LUMO (22%)
11	21272.9	470.1	0.0072	Singlet-A	H-6->LUMO (15%), H-2->L+1 (26%)
12	21435.0	466.5	0.0427	Singlet-A	H-10->L+5 (11%), H-9->LUMO (14%), H-9->L+6 (13%)
13	21770.5	459.3	0.0072	Singlet-A	H-1->L+2 (44%), HOMO->L+3 (45%)
14	21801.2	458.7	0.0423	Singlet-A	H-10->LUMO (21%), H-9->LUMO (15%), H-9->L+1 (17%), H-2->L+1 (18%)
15	21931.0	456.0	0.0622	Singlet-A	H-2->LUMO (12%)
16	21985.1	454.9	0.0974	Singlet-A	H-14->L+5 (17%), H-5->L+5 (16%), H-4->L+6 (15%)
17	22006.0	454.4	0.0331	Singlet-A	H-14->L+6 (11%), H-4->L+5 (11%), H-2->LUMO (17%)
18	22043.9	453.6	0.0027	Singlet-A	H-9->LUMO (10%), H-3->L+1 (18%)
19	22323.8	448.0	0.2422	Singlet-A	H-10->L+5 (10%), H-10->L+6 (10%), H-9->L+5 (11%), H-3->LUMO (10%), H-2->L+1 (28%)
20	22624.7	442.0	0.4072	Singlet-A	H-4->LUMO (18%), H-3->LUMO (38%)
21	23061.8	433.6	0.063	Singlet-A	H-5->LUMO (13%), H-3->L+1 (19%), HOMO->L+4 (18%)
22	23173.9	431.5	0.4434	Singlet-A	H-6->LUMO (49%)
23	23673.2	422.4	0.0279	Singlet-A	H-6->L+1 (40%), HOMO->L+4 (15%)
24	23911.9	418.2	0.0031	Singlet-A	H-7->L+1 (56%)
25	23936.1	417.8	0.0065	Singlet-A	H-7->L+1 (10%), H-5->LUMO (10%), H-4->L+2 (11%), H-3->L+2 (13%), HOMO->L+4 (12%)
26	24155.5	414.0	0.0254	Singlet-A	H-5->L+1 (21%), H-5->L+2 (11%), H-4->LUMO (20%)
27	24253.1	412.3	2E-4	Singlet-A	H-11->L+1 (11%), H-7->LUMO (66%)
28	24338.6	410.9	0.0251	Singlet-A	H-2->L+2 (67%), H-1->L+3 (13%)
29	24544.3	407.4	0.0946	Singlet-A	H-5->LUMO (17%), H-3->L+2 (32%)
30	24749.9	404.0	0.0024	Singlet-A	H-11->L+1 (13%), H-7->LUMO (15%), H-6->L+2 (11%), H-4->L+1 (15%), HOMO->L+4 (21%)
31	25046.7	399.3	0.0078	Singlet-A	H-5->L+1 (10%), H-5->L+2 (25%), H-2->L+2 (13%), H-1->L+3 (20%)
32	25180.6	397.1	0.0018	Singlet-A	H-8->LUMO (58%)
33	25208.9	396.7	0.016	Singlet-A	H-12->L+1 (31%), H-11->LUMO (38%)
34	25351.6	394.5	0.022	Singlet-A	H-12->LUMO (14%), H-11->L+1 (26%), H-8->LUMO (26%)
35	25658.1	389.7	0.3927	Singlet-A	H-12->L+1 (10%), H-5->L+2 (15%), H-1->L+3 (31%)
36	25820.2	387.3	0.0038	Singlet-A	H-9->LUMO (10%), H-9->L+1 (20%), H-9->L+2 (49%)
37	25849.3	386.9	0.0286	Singlet-A	H-10->L+1 (18%), H-10->L+2 (44%)
38	25870.2	386.5	0.1342	Singlet-A	H-13->LUMO (24%), H-5->L+2 (10%), H-4->L+2 (11%), H-3->L+2 (16%)
39	25886.4	386.3	0.0809	Singlet-A	H-13->LUMO (41%), H-10->L+2 (11%), H-4->L+2 (11%)
40	26121.1	382.8	0.0192	Singlet-A	H-8->L+1 (77%)
41	26242.1	381.1	0.0242	Singlet-A	H-15->LUMO (15%), H-12->L+1 (24%), H-11->LUMO (27%)
42	26316.3	380.0	0.0085	Singlet-A	H-15->LUMO (40%), H-15->L+1 (20%)
43	26395.3	378.9	0.0047	Singlet-A	H-16->LUMO (33%), H-16->L+1 (12%), H-12->LUMO (31%)
44	26397.7	378.8	0.0091	Singlet-A	H-16->LUMO (22%), H-12->LUMO (31%)
45	26710.7	374.4	0.0628	Singlet-A	H-13->L+1 (73%)
46	26909.1	371.6	0.0232	Singlet-A	H-14->LUMO (52%), H-6->L+2 (25%)
47	27051.8	369.7	5E-4	Singlet-A	H-14->L+1 (37%), H-1->L+4 (28%)
48	27084.9	369.2	0.0115	Singlet-A	H-14->L+1 (34%), H-1->L+4 (43%)
49	27273.6	366.7	1E-4	Singlet-A	H-9->LUMO (30%), H-9->L+1 (33%), H-9->L+2 (24%)
50	27353.5	365.6	4E-4	Singlet-A	H-10->LUMO (24%), H-10->L+1 (34%), H-10->L+2 (20%)
51	27392.2	365.1	0.0016	Singlet-A	H-14->LUMO (10%), H-6->L+2 (14%), H-4->L+2 (10%), H-2->L+3 (15%)
52	27564.0	362.8	0.001	Singlet-A	H-17->LUMO (59%), H-17->L+1 (28%)
53	27628.5	361.9	0.0036	Singlet-A	H-7->L+2 (78%)

54	27651.1	361.6	0.0019	Singlet-A	H-18->LUMO (60%), H-18->L+1 (21%)
55	28133.4	355.4	0.1108	Singlet-A	H-8->L+2 (82%)
56	28172.1	355.0	0.2263	Singlet-A	H-2->L+3 (49%)
57	28229.4	354.2	0.0053	Singlet-A	H-33->L+6 (37%), H-32->L+5 (31%)
58	28250.4	354.0	0.0582	Singlet-A	H-33->L+5 (28%), H-32->L+6 (33%), H-2->L+3 (12%)
59	28304.4	353.3	3E-4	Singlet-A	H-19->L+1 (80%)
60	28328.6	353.0	0.0092	Singlet-A	H-3->L+3 (74%)

Table S4. TD DFT calculated electronic transitions for [**3c**$^{\bullet}$]$^{+}$ (*in vacuo*).

No	Energy (cm^{-1})	Wavelength (nm)	Osc. Strength	Symmetry	Major contributions
1	5447.5	1835.7	0.2295	2.016-A	HOMO(B)->LUMO(B) (98%)
2	7753.4	1289.8	0.0114	2.042-A	H-1(B)->LUMO(B) (91%)
3	8679.3	1152.2	0.0421	2.043-A	H-2(B)->LUMO(B) (81%)
4	9847.2	1015.5	5E-4	2.069-A	H-4(B)->LUMO(B) (32%), H-3(B)->LUMO(B) (38%)
5	9914.2	1008.7	0.0102	2.108-A	H-14(B)->LUMO(B) (10%), H-5(B)->LUMO(B) (60%)
6	10098.9	990.2	1E-4	2.138-A	H-7(B)->LUMO(B) (38%), H-3(B)->LUMO(B) (38%)
7	10236.0	976.9	0.0049	2.185-A	H-11(B)->LUMO(B) (60%)
8	10522.3	950.4	5E-4	3.234-A	--
9	10576.3	945.5	4E-4	3.292-A	H-17(A)->L+6(A) (38%), H-16(B)->L+7(B) (37%)
10	10705.4	934.1	0.0031	2.433-A	H-7(B)->LUMO(B) (10%), H-5(B)->LUMO(B) (11%), H-4(B)->LUMO(B) (39%)
11	10997.4	909.3	0	3.418-A	H-19(A)->L+5(A) (40%), H-18(B)->L+6(B) (39%)
12	11079.6	902.6	1E-4	3.314-A	H-15(A)->L+5(A) (13%), H-18(B)->L+6(B) (10%)
13	11248.2	889.0	0	3.426-A	H-13(A)->L+6(A) (14%), H-11(B)->L+7(B) (15%)
14	11485.3	870.7	0.003	2.077-A	H-11(B)->LUMO(B) (11%), H-8(B)->LUMO(B) (68%)
15	11648.3	858.5	8E-4	2.978-A	H-18(A)->L+5(A) (10%), H-17(B)->L+6(B) (13%), H-13(B)->LUMO(B) (19%)
16	11709.6	854.0	0.0026	2.627-A	H-13(B)->LUMO(B) (50%)
17	12143.5	823.5	2E-4	2.085-A	H-14(B)->LUMO(B) (63%), H-13(B)->LUMO(B) (10%), H-7(B)->LUMO(B) (11%)
18	12468.5	802.0	0.0279	2.415-A	HOMO(A)->LUMO(A) (57%)
19	12632.3	791.6	1E-4	2.035-A	H-6(B)->LUMO(B) (89%)
20	12660.5	789.9	0.0011	2.041-A	H-9(B)->LUMO(B) (91%)
21	12743.6	784.7	0	2.059-A	H-16(B)->LUMO(B) (86%)
22	12888.7	775.9	0.0112	3.019-A	H-1(A)->LUMO(A) (13%), HOMO(A)->L+1(A) (33%), HOMO(B)->L+1(B) (21%), HOMO(B)->L+2(B) (11%)
23	12970.2	771.0	2E-4	2.096-A	H-18(B)->LUMO(B) (94%)
24	13239.6	755.3	0	2.024-A	H-10(B)->LUMO(B) (97%)
25	13308.1	751.4	0	2.027-A	H-12(B)->LUMO(B) (96%)
26	13538.8	738.6	0.0077	2.244-A	H-17(B)->LUMO(B) (79%)
27	14087.3	709.9	5E-4	2.024-A	H-15(B)->LUMO(B) (92%)
28	15347.1	651.6	0.063	2.794-A	HOMO(A)->LUMO(A) (18%), HOMO(A)->L+1(A) (22%), HOMO(B)->L+1(B) (21%)
29	15509.2	644.8	0.0107	3.166-A	H-2(A)->LUMO(A) (10%), H-2(A)->L+1(A) (10%), HOMO(B)->L+3(B) (11%)
30	16197.2	617.4	0.0198	3.002-A	H-1(A)->L+2(A) (10%), HOMO(A)->L+1(A) (10%), HOMO(B)->L+2(B) (37%), HOMO(B)->L+3(B) (14%)
31	16316.6	612.9	0.0323	2.719-A	H-1(A)->LUMO(A) (16%), HOMO(B)->L+1(B) (31%)
32	16880.4	592.4	0.1744	2.311-A	HOMO(A)->L+2(A) (41%), H-19(B)->LUMO(B) (15%), HOMO(B)->L+1(B) (13%)
33	17184.4	581.9	0.0037	2.521-A	HOMO(A)->L+2(A) (11%), H-19(B)->LUMO(B) (47%)
34	17278.0	578.8	0.0387	2.784-A	HOMO(B)->L+2(B) (17%), HOMO(B)->L+3(B) (20%)
35	17736.1	563.8	0.0173	2.617-A	H-1(A)->LUMO(A) (31%), HOMO(B)->L+2(B) (11%)
36	18354.0	544.8	0.0025	2.644-A	H-1(A)->L+1(A) (11%), H-21(B)->LUMO(B) (43%)
37	18798.4	532.0	0.014	2.693-A	H-1(A)->L+1(A) (33%)
38	19124.2	522.9	0.0085	3.276-A	--
39	19560.6	511.2	0.0011	2.989-A	H-21(B)->LUMO(B) (13%)
40	19719.4	507.1	5E-4	3.155-A	H-22(B)->LUMO(B) (11%)
41	20044.5	498.9	0.0028	2.286-A	H-24(B)->LUMO(B) (19%), H-22(B)->LUMO(B) (57%)
42	20129.2	496.8	0.0063	2.462-A	H-2(A)->LUMO(A) (10%), HOMO(A)->L+3(A) (21%)
43	20265.5	493.4	3E-4	2.052-A	H-20(B)->LUMO(B) (89%)
44	20327.6	491.9	0.0029	2.863-A	H-1(A)->L+2(A) (12%), HOMO(B)->L+3(B) (16%)
45	20596.2	485.5	0.0242	3.159-A	H-11(B)->L+1(B) (10%)
46	20860.7	479.4	0.0029	3.198-A	H-18(A)->LUMO(A) (12%), H-13(A)->L+1(A) (13%)
47	20925.2	477.9	0.0034	2.073-A	HOMO(A)->L+6(A) (10%), H-7(B)->L+7(B) (11%)
48	20985.7	476.5	0.008	2.815-A	H-3(A)->LUMO(A) (57%), H-3(A)->L+1(A) (10%)
49	21005.1	476.1	0.0072	2.414-A	H-2(A)->LUMO(A) (30%), H-1(B)->L+1(B) (15%)
50	21157.5	472.6	2E-4	2.044-A	H-15(A)->L+5(A) (12%), HOMO(A)->L+5(A) (12%), H-11(B)->L+6(B) (12%)

51	21320.5	469.0	0.0311	2.646-A	H-4(A)->LUMO(A) (27%)
52	21393.9	467.4	0.0108	2.556-A	H-1(A)->L+2(A) (12%), HOMO(A)->L+3(A) (12%), H-23(B)->LUMO(B) (11%), H-2(B)->L+1(B) (10%)
53	21518.1	464.7	0.0019	2.709-A	H-24(B)->LUMO(B) (23%)
54	21562.4	463.8	0.0171	2.629-A	H-5(A)->LUMO(A) (10%), H-4(A)->LUMO(A) (21%), H-2(A)->L+1(A) (17%), H-1(B)->L+2(B) (11%)
55	21634.2	462.2	2E-4	3.392-A	H-30(A)->L+6(A) (39%), H-31(B)->L+7(B) (39%)
56	21641.5	462.1	0.0022	2.512-A	H-5(A)->LUMO(A) (13%), H-4(A)->LUMO(A) (10%), HOMO(A)->L+4(A) (18%)
57	21701.2	460.8	0.0147	2.636-A	HOMO(A)->L+4(A) (12%)
58	21858.4	457.5	0.0035	2.758-A	H-3(A)->L+1(A) (17%)
59	21937.5	455.8	0.0014	2.247-A	H-11(B)->L+7(B) (12%)
60	21943.9	455.7	1E-4	2.878-A	H-9(A)->LUMO(A) (51%), H-8(A)->LUMO(A) (30%)

Table S5. TD DFT calculated electronic transitions for [**3c**$^{\bullet}$]$^{+}$ (DCM).

No	Energy (cm^{-1})	Wavelength (nm)	Osc. Strength	Symmetry	Major contributions
1	5099.8	1960.8	0.3342	2.016-A	HOMO(B)->LUMO(B) (98%)
2	7982.5	1252.7	0.0079	2.044-A	H-2(B)->LUMO(B) (14%), H-1(B)->LUMO(B) (82%)
3	8685.0	1151.4	0.0448	2.047-A	H-2(B)->LUMO(B) (72%), H-1(B)->LUMO(B) (14%)
4	9785.1	1022.0	0.0109	2.173-A	H-9(B)->LUMO(B) (17%), H-7(B)->LUMO(B) (16%), H-6(B)->LUMO(B) (38%)
5	9912.6	1008.8	0	2.768-A	H-16(A)->L+6(A) (20%), H-14(B)->L+7(B) (23%), H-7(B)->LUMO(B) (23%), H-4(B)->LUMO(B) (17%)
6	10035.2	996.5	0.0016	2.820-A	H-16(A)->L+6(A) (24%), H-14(B)->L+7(B) (22%), H-7(B)->LUMO(B) (20%), H-6(B)->LUMO(B) (22%)
7	10079.5	992.1	0.0092	2.805-A	H-5(B)->LUMO(B) (10%), H-4(B)->LUMO(B) (33%)
8	10326.3	968.4	4E-4	3.380-A	H-18(A)->L+5(A) (44%), H-18(B)->L+6(B) (44%)
9	10384.4	963.0	0.002	2.976-A	H-4(B)->LUMO(B) (23%)
10	10621.5	941.5	5E-4	2.084-A	H-3(B)->LUMO(B) (84%)
11	10890.1	918.3	0	3.325-A	H-12(A)->L+5(A) (13%), H-9(B)->L+6(B) (10%), H-7(B)->L+6(B) (16%)
12	11028.0	906.8	1E-4	3.435-A	H-6(A)->L+6(A) (11%)
13	11403.9	876.9	9E-4	3.225-A	H-19(A)->L+5(A) (12%), H-16(B)->L+6(B) (17%), H-5(B)->LUMO(B) (10%)
14	11475.7	871.4	0.0034	2.337-A	H-7(B)->LUMO(B) (12%), H-5(B)->LUMO(B) (56%), H-4(B)->LUMO(B) (10%)
15	11989.4	834.1	1E-4	2.112-A	H-18(B)->LUMO(B) (90%)
16	12134.6	824.1	0.0033	2.036-A	H-10(B)->LUMO(B) (24%), H-9(B)->LUMO(B) (54%)
17	12201.6	819.6	0.0023	2.028-A	H-8(B)->LUMO(B) (95%)
18	12342.7	810.2	4E-4	2.063-A	H-14(B)->LUMO(B) (82%)
19	12426.6	804.7	0.0205	2.236-A	HOMO(A)->LUMO(A) (34%), H-14(B)->LUMO(B) (10%), H-10(B)->LUMO(B) (17%)
20	12480.6	801.2	0.0186	2.285-A	HOMO(A)->LUMO(A) (20%), H-10(B)->LUMO(B) (32%), H-9(B)->LUMO(B) (10%)
21	12844.4	778.6	0.0176	2.952-A	H-1(A)->LUMO(A) (17%), HOMO(A)->L+1(A) (31%), HOMO(B)->L+1(B) (23%)
22	13340.4	749.6	0.0129	2.221-A	H-16(B)->LUMO(B) (76%)
23	14162.3	706.1	2E-4	2.032-A	H-12(B)->LUMO(B) (98%)
24	14453.5	691.9	1E-4	2.027-A	H-11(B)->LUMO(B) (92%)
25	14743.8	678.3	4E-4	2.023-A	H-17(B)->LUMO(B) (96%)
26	14885.0	671.8	0	2.027-A	H-15(B)->LUMO(B) (99%)
27	15132.6	660.8	0	2.024-A	H-13(B)->LUMO(B) (100%)
28	15271.3	654.8	0.103	2.802-A	HOMO(A)->LUMO(A) (10%), HOMO(A)->L+1(A) (26%), HOMO(A)->L+2(A) (12%), HOMO(B)->L+1(B) (19%)
29	15501.2	645.1	0.005	3.223-A	H-2(A)->LUMO(A) (12%), H-1(B)->L+1(B) (10%), HOMO(B)->L+3(B) (12%)
30	16210.9	616.9	0.0112	2.802-A	H-1(A)->LUMO(A) (11%), HOMO(A)->L+1(A) (10%), HOMO(B)->L+2(B) (38%)
31	16317.4	612.8	0.0641	2.877-A	HOMO(B)->L+1(B) (29%)
32	16718.3	598.1	0.2317	2.371-A	HOMO(A)->L+2(A) (54%), HOMO(B)->L+1(B) (12%)
33	17137.7	583.5	0.0171	2.762-A	H-20(B)->LUMO(B) (13%), H-19(B)->LUMO(B) (23%), HOMO(B)->L+3(B) (16%)
34	17269.9	579.0	0.0306	2.457-A	H-19(B)->LUMO(B) (36%), HOMO(B)->L+2(B) (14%)
35	17768.4	562.8	0.0236	2.669-A	H-1(A)->LUMO(A) (30%)
36	18400.7	543.5	0.0027	2.713-A	H-1(A)->L+1(A) (10%), H-20(B)->LUMO(B) (38%)
37	18820.1	531.3	0.0146	2.733-A	H-1(A)->L+1(A) (31%)
38	19073.4	524.3	0.0068	3.280-A	--
39	19470.2	513.6	0.0018	3.113-A	H-20(B)->LUMO(B) (10%)
40	19644.4	509.0	0.0014	3.125-A	H-12(A)->LUMO(A) (10%)

41	20024.3	499.4	0.0144	2.233-A	H-1(A)->L+2(A) (12%), HOMO(A)->L+3(A) (30%), HOMO(B)->L+3(B) (20%)
42	20243.7	494.0	0.0076	3.074-A	H-7(A)->LUMO(A) (12%)
43	20443.7	489.1	0.0186	2.625-A	H-24(B)->LUMO(B) (16%), H-23(B)->LUMO(B) (39%)
44	20558.3	486.4	0.0167	2.862-A	H-24(B)->LUMO(B) (27%), H-23(B)->LUMO(B) (10%)
45	20707.5	482.9	0.0021	3.186-A	H-19(A)->LUMO(A) (12%), H-6(B)->L+2(B) (10%)
46	20759.9	481.7	0.0031	2.039-A	H-7(A)->L+6(A) (13%), HOMO(A)->L+6(A) (10%), HOMO(B)->L+7(B) (10%)
47	21025.3	475.6	0.0137	2.208-A	H-2(A)->LUMO(A) (26%), H-1(B)->L+1(B) (17%)
48	21064.8	474.7	2E-4	2.051-A	HOMO(A)->L+5(A) (10%), H-7(B)->L+6(B) (17%)
49	21326.1	468.9	0.0172	2.609-A	H-1(A)->L+2(A) (13%), HOMO(A)->L+3(A) (11%)
50	21367.2	468.0	0.0484	2.404-A	H-2(A)->LUMO(A) (12%), H-2(A)->L+1(A) (10%), H-2(B)->L+1(B) (10%)
51	21466.4	465.8	0.0116	2.680-A	H-24(B)->LUMO(B) (11%)
52	21499.5	465.1	0.0088	2.583-A	H-4(A)->LUMO(A) (11%)
53	21545.5	464.1	0.0014	3.305-A	H-30(A)->L+6(A) (35%), H-31(B)->L+7(B) (34%)
54	21585.0	463.3	0.0128	2.438-A	H-3(A)->LUMO(A) (12%), HOMO(A)->L+4(A) (12%)
55	21655.2	461.8	0.0062	2.608-A	H-4(A)->LUMO(A) (16%)
56	21774.5	459.3	0.0019	2.359-A	--
57	21840.7	457.9	0.001	2.820-A	H-33(A)->L+5(A) (18%), H-33(B)->L+6(B) (17%)
58	21843.9	457.8	0.0192	2.661-A	H-4(A)->LUMO(A) (15%)
59	21910.9	456.4	0.0031	3.013-A	H-16(A)->LUMO(A) (12%), H-16(A)->L+1(A) (41%)
60	21969.7	455.2	0.0105	2.752-A	H-16(A)->L+1(A) (13%), HOMO(A)->L+4(A) (16%)

Table S6. TD DFT calculated electronic transitions for triplet $[\mathbf{3c}^{\bullet\bullet}]^{2+}$ (*in vacuo*)

No	Energy (cm^{-1})	Wavelength (nm)	Osc. Strength	Symmetry	Major contributions
1	5866.1	1704.7	0.0142	3.018-A	HOMO(B)->LUMO(B) (75%)
2	6379.0	1567.6	0.0074	3.016-A	H-2(B)->LUMO(B) (90%)
3	6554.1	1525.8	0.0036	3.023-A	H-7(B)->LUMO(B) (46%), H-1(B)->LUMO(B) (24%)
4	6725.1	1487.0	0.006	3.021-A	H-7(B)->LUMO(B) (22%), H-1(B)->LUMO(B) (47%)
5	6851.7	1459.5	0.0209	3.021-A	H-9(B)->LUMO(B) (17%), H-6(B)->LUMO(B) (37%), H-1(B)->LUMO(B) (17%), HOMO(B)->LUMO(B) (11%)
6	7072.7	1413.9	0.0019	3.022-A	H-4(B)->LUMO(B) (86%)
7	7268.7	1375.8	0	3.019-A	H-3(B)->LUMO(B) (90%)
8	7567.9	1321.4	3E-4	3.021-A	H-8(B)->LUMO(B) (72%)
9	7630.8	1310.5	0	3.020-A	H-5(B)->LUMO(B) (86%)
10	8010.7	1248.3	0.0179	3.022-A	H-9(B)->LUMO(B) (44%), H-6(B)->LUMO(B) (34%)
11	8572.9	1166.5	0.0067	3.034-A	H-13(B)->LUMO(B) (24%), H-12(B)->LUMO(B) (35%), H-9(B)->LUMO(B) (11%)
12	8710.0	1148.1	0.0049	3.024-A	H-13(B)->LUMO(B) (10%), H-11(B)->LUMO(B) (11%), H-10(B)->LUMO(B) (21%), H-9(B)->L+1(B) (13%), H-7(B)->LUMO(B) (15%), HOMO(B)->L+1(B) (13%)
13	8989.0	1112.5	2E-4	3.038-A	H-14(B)->LUMO(B) (18%), H-13(B)->LUMO(B) (18%), H-11(B)->LUMO(B) (20%)
14	9280.2	1077.6	0.0044	3.022-A	H-12(B)->LUMO(B) (10%), HOMO(B)->L+1(B) (61%)
15	9550.4	1047.1	1E-4	3.042-A	H-14(B)->LUMO(B) (51%), H-13(B)->LUMO(B) (12%)
16	10156.9	984.5	0.0022	3.023-A	H-2(B)->L+1(B) (83%)
17	10541.7	948.6	0.0017	3.021-A	H-10(B)->LUMO(B) (45%), H-6(B)->L+1(B) (30%)
18	10812.7	924.8	0.0129	3.035-A	H-7(B)->L+1(B) (47%), H-1(B)->L+1(B) (22%)
19	10884.5	918.7	0.0013	3.033-A	H-7(B)->L+1(B) (16%), H-1(B)->L+1(B) (65%)
20	10976.4	911.0	0.0109	3.284-A	H-16(B)->LUMO(B) (26%), H-14(B)->L+1(B) (11%), H-7(B)->L+1(B) (14%)
21	11038.5	905.9	0.0012	3.070-A	H-11(B)->LUMO(B) (11%), H-10(B)->LUMO(B) (12%), H-6(B)->L+1(B) (49%)
22	11083.7	902.2	0.0012	3.589-A	H-17(B)->LUMO(B) (21%), H-17(B)->L+1(B) (11%), H-17(B)->L+8(B) (13%)
23	11118.4	899.4	0.003	3.517-A	H-20(A)->L+5(A) (14%), H-18(B)->LUMO(B) (22%), H-18(B)->L+7(B) (13%)
24	11292.6	885.5	0	3.025-A	H-3(B)->L+1(B) (91%)
25	11426.5	875.2	9E-4	3.030-A	H-8(B)->L+1(B) (20%), H-4(B)->L+1(B) (68%)
26	11467.6	872.0	9E-4	3.057-A	H-8(B)->L+1(B) (57%), H-4(B)->L+1(B) (23%)
27	11615.2	860.9	0	3.843-A	H-17(B)->LUMO(B) (14%), H-17(B)->L+8(B) (10%)
28	11629.7	859.9	1E-4	3.859-A	H-18(B)->LUMO(B) (14%)
29	11653.9	858.1	1E-4	3.812-A	H-14(A)->L+6(A) (11%), H-17(B)->LUMO(B) (20%)
30	11674.1	856.6	0	3.792-A	H-15(A)->L+5(A) (11%), H-18(B)->LUMO(B) (23%)
31	11856.4	843.4	0	3.024-A	H-5(B)->L+1(B) (92%)
32	12133.8	824.1	2E-4	4.027-A	H-16(A)->L+5(A) (10%), H-16(B)->L+8(B) (11%)
33	12183.0	820.8	0	4.017-A	H-16(A)->L+6(A) (10%), H-16(B)->L+7(B) (12%), H-14(B)->L+8(B) (10%)

34	12288.7	813.8	6E-4	3.037-A	H-11(B)->LUMO(B) (26%), H-9(B)->L+1(B) (63%)
35	13012.9	768.5	0.0664	3.034-A	HOMO(A)->LUMO(A) (86%)
36	13228.3	756.0	0.0219	3.039-A	H-10(B)->L+1(B) (77%)
37	13822.7	723.4	0.0107	3.033-A	HOMO(A)->L+1(A) (15%), H-11(B)->L+1(B) (55%), H-10(B)->L+1(B) (15%)
38	14274.4	700.6	0.0106	3.025-A	H-1(A)->LUMO(A) (14%), HOMO(A)->L+1(A) (50%), H-11(B)->L+1(B) (25%)
39	14681.7	681.1	0.0016	3.059-A	H-19(B)->LUMO(B) (10%), H-12(B)->LUMO(B) (14%), H-12(B)->L+1(B) (56%)
40	15022.1	665.7	5E-4	3.131-A	H-19(B)->LUMO(B) (15%), H-13(B)->L+1(B) (37%)
41	15133.4	660.8	5E-4	3.024-A	H-15(B)->LUMO(B) (96%)
42	15226.9	656.7	0.0085	3.221-A	H-20(B)->LUMO(B) (18%), H-16(B)->LUMO(B) (14%), H-14(B)->L+1(B) (22%)
43	15401.2	649.3	0.0048	3.235-A	H-19(B)->LUMO(B) (23%), H-13(B)->L+1(B) (28%)
44	16210.9	616.9	0.0486	3.326-A	H-1(A)->LUMO(A) (17%), H-16(B)->LUMO(B) (14%), H-14(B)->L+1(B) (21%)
45	16608.6	602.1	0.0182	3.606-A	H-12(A)->LUMO(A) (11%), H-16(B)->L+1(B) (19%)
46	16808.6	594.9	0.1933	3.204-A	HOMO(A)->L+2(A) (54%)
47	17474.0	572.3	7E-4	3.134-A	H-1(A)->LUMO(A) (21%), HOMO(A)->L+2(A) (17%), H-20(B)->LUMO(B) (22%)
48	17747.4	563.5	6E-4	3.246-A	H-2(A)->LUMO(A) (10%), H-19(B)->LUMO(B) (10%), H-16(B)->L+1(B) (30%)
49	17827.3	560.9	0.0026	3.544-A	H-2(A)->LUMO(A) (21%), H-2(A)->L+1(A) (20%)
50	17911.2	558.3	0.0012	3.632-A	H-3(A)->LUMO(A) (53%), H-3(A)->L+1(A) (19%)
51	18033.8	554.5	0.0022	3.490-A	H-2(A)->LUMO(A) (28%), H-2(A)->L+1(A) (12%), H-1(A)->L+1(A) (16%)
52	18096.7	552.6	0.0023	3.618-A	H-6(A)->LUMO(A) (65%), H-6(A)->L+1(A) (10%), H-6(A)->L+2(A) (10%)
53	18151.5	550.9	0.0148	3.308-A	H-2(A)->LUMO(A) (25%), H-1(A)->L+1(A) (15%)
54	18418.5	542.9	0.0093	3.609-A	H-4(A)->LUMO(A) (83%)
55	18509.6	540.3	0.015	3.522-A	H-2(A)->L+1(A) (40%)
56	18592.7	537.8	1E-4	3.632-A	H-5(A)->LUMO(A) (68%), H-5(A)->L+1(A) (21%)
57	18815.3	531.5	1E-4	3.629-A	H-7(A)->LUMO(A) (78%), H-7(A)->L+1(A) (11%)
58	18825.8	531.2	1E-4	3.027-A	H-15(B)->L+1(B) (97%)
59	19008.1	526.1	0.0033	3.483-A	H-9(A)->L+1(A) (12%), H-8(A)->LUMO(A) (31%), H-1(A)->L+2(A) (12%)
60	19022.6	525.7	2E-4	3.057-A	H-17(B)->LUMO(B) (29%), H-17(B)->L+1(B) (63%)

Table S7. TD DFT calculated electronic transitions for triplet $[\mathbf{3c}^{\bullet\bullet}]^{2+}$ (DCM).

No	Energy (cm^{-1})	Wavelength (nm)	Osc. Strength	Symmetry	Major contributions
1	6434.7	1554.1	0.0684	3.022-A	H-3(B)->LUMO(B) (20%), H-1(B)->LUMO(B) (10%), HOMO(B)->LUMO(B) (50%)
2	6931.5	1442.7	0.0127	3.026-A	H-2(B)->LUMO(B) (65%)
3	7852.6	1273.5	0.0018	3.032-A	H-12(B)->LUMO(B) (34%), H-3(B)->LUMO(B) (13%), HOMO(B)->LUMO(B) (22%)
4	8026.8	1245.8	0.0067	3.029-A	H-14(B)->LUMO(B) (13%), H-10(B)->LUMO(B) (13%), H-4(B)->LUMO(B) (29%), H-1(B)->LUMO(B) (11%)
5	8097.8	1234.9	0.0091	3.018-A	H-1(B)->LUMO(B) (72%)
6	8405.9	1189.6	0.0028	3.023-A	H-12(B)->LUMO(B) (22%), H-3(B)->LUMO(B) (47%), HOMO(B)->LUMO(B) (14%)
7	8791.4	1137.5	9E-4	3.037-A	H-14(B)->LUMO(B) (20%), H-13(B)->LUMO(B) (13%), H-11(B)->LUMO(B) (15%), H-10(B)->LUMO(B) (26%)
8	9268.1	1079.0	3E-4	3.032-A	H-14(B)->LUMO(B) (15%), H-11(B)->LUMO(B) (19%), H-4(B)->LUMO(B) (22%), H-2(B)->LUMO(B) (23%)
9	9642.4	1037.1	0.0118	3.018-A	H-5(B)->LUMO(B) (79%)
10	9877.1	1012.4	3E-4	3.251-A	H-16(B)->LUMO(B) (47%), H-16(B)->L+1(B) (23%)
11	9895.6	1010.5	2E-4	3.266-A	H-17(B)->LUMO(B) (46%), H-17(B)->L+1(B) (21%)
12	10047.2	995.3	0.0035	3.044-A	H-11(B)->LUMO(B) (18%), H-4(B)->LUMO(B) (24%)
13	10355.4	965.7	0.0049	3.238-A	H-15(B)->LUMO(B) (33%), H-14(B)->L+1(B) (10%)
14	10562.6	946.7	0.0018	3.140-A	H-6(B)->LUMO(B) (72%)
15	10590.9	944.2	0	3.933-A	H-18(A)->L+5(A) (19%), H-17(A)->L+6(A) (14%), H-17(B)->LUMO(B) (10%), H-17(B)->L+7(B) (17%), H-16(B)->L+8(B) (13%)
16	10646.5	939.3	0.0012	3.487-A	H-7(B)->LUMO(B) (28%)
17	10670.7	937.1	0.0026	3.266-A	H-7(B)->LUMO(B) (58%)
18	10911.1	916.5	0.003	3.020-A	H-1(B)->L+1(B) (14%), HOMO(B)->L+1(B) (63%)
19	11173.2	895.0	1E-4	3.019-A	H-8(B)->LUMO(B) (92%)
20	11281.3	886.4	1E-4	3.019-A	H-9(B)->LUMO(B) (92%)
21	11385.3	878.3	1E-4	4.094-A	H-15(A)->L+6(A) (11%), H-12(B)->L+8(B) (10%)
22	11393.4	877.7	2E-4	4.091-A	H-15(A)->L+5(A) (17%), H-12(B)->L+7(B) (15%)

23	11612.8	861.1	0.0065	3.021-A	H-10(B)->LUMO(B) (17%), H-1(B)->L+1(B) (48%), HOMO(B)->L+1(B) (12%)
24	11784.6	848.6	0.0473	3.028-A	H-2(B)->L+1(B) (77%)
25	11899.1	840.4	2E-4	4.011-A	H-19(A)->L+6(A) (13%), H-16(A)->L+5(A) (10%), H-15(B)->L+8(B) (13%)
26	11928.9	838.3	1E-4	4.011-A	H-19(A)->L+5(A) (13%), H-15(B)->L+7(B) (14%)
27	12081.4	827.7	4E-4	3.033-A	H-3(B)->L+1(B) (55%), H-1(B)->L+1(B) (21%)
28	12609.7	793.0	0.005	3.029-A	H-4(B)->L+1(B) (82%)
29	12664.5	789.6	1E-4	3.017-A	H-14(B)->LUMO(B) (10%), H-13(B)->LUMO(B) (52%), H-3(B)->L+1(B) (23%)
30	12877.4	776.6	0.0874	3.029-A	HOMO(A)->LUMO(A) (84%)
31	13120.2	762.2	0.0087	3.027-A	H-5(B)->L+1(B) (83%)
32	13780.0	725.7	0.0167	3.040-A	HOMO(A)->L+1(A) (39%), H-10(B)->L+1(B) (26%)
33	14145.4	706.9	0.0227	3.026-A	H-1(A)->LUMO(A) (12%), HOMO(A)->L+1(A) (31%), H-11(B)->L+1(B) (10%), H-10(B)->L+1(B) (33%)
34	14453.5	691.9	0.0015	3.051-A	H-12(B)->L+1(B) (23%), H-11(B)->L+1(B) (31%)
35	14608.3	684.5	5E-4	3.057-A	H-12(B)->LUMO(B) (12%), H-12(B)->L+1(B) (35%), H-11(B)->L+1(B) (18%)
36	14793.8	676.0	7E-4	3.030-A	H-6(B)->L+1(B) (76%)
37	14972.1	667.9	5E-4	3.034-A	H-7(B)->L+1(B) (84%)
38	15092.2	662.6	0.0129	3.166-A	H-20(B)->LUMO(B) (15%), H-18(B)->L+1(B) (10%), H-15(B)->LUMO(B) (18%), H-14(B)->L+1(B) (17%), H-13(B)->L+1(B) (12%)
39	15234.2	656.4	0.0024	3.199-A	H-18(B)->LUMO(B) (32%), H-8(B)->L+1(B) (21%)
40	15251.1	655.7	0.001	3.073-A	H-8(B)->L+1(B) (70%)
41	15435.0	647.9	1E-4	3.025-A	H-9(B)->L+1(B) (90%)
42	15473.7	646.3	0.0063	3.030-A	H-14(B)->L+1(B) (24%), H-13(B)->L+1(B) (68%)
43	16050.4	623.0	0.1202	3.272-A	H-1(A)->LUMO(A) (19%), HOMO(A)->L+2(A) (19%), H-15(B)->LUMO(B) (10%), H-14(B)->L+1(B) (14%)
44	16482.7	606.7	0.0313	3.560-A	H-15(B)->L+1(B) (27%)
45	16568.2	603.6	0.2242	3.254-A	HOMO(A)->L+2(A) (51%)
46	17420.0	574.1	0.0022	3.111-A	H-1(A)->LUMO(A) (28%), HOMO(A)->L+1(A) (11%), HOMO(A)->L+2(A) (13%), H-20(B)->LUMO(B) (22%)
47	17603.9	568.1	0	3.240-A	H-1(A)->L+1(A) (17%), H-18(B)->LUMO(B) (17%), H-15(B)->L+1(B) (24%)
48	17996.6	555.7	0.0391	3.175-A	H-1(A)->L+1(A) (38%), HOMO(A)->L+3(A) (12%)
49	18190.2	549.7	0.0185	3.479-A	H-1(A)->LUMO(A) (20%)
50	18254.7	547.8	0	3.024-A	H-16(B)->LUMO(B) (30%), H-16(B)->L+1(B) (67%)
51	18426.5	542.7	0	3.024-A	H-17(B)->LUMO(B) (28%), H-17(B)->L+1(B) (69%)
52	18778.2	532.5	0.002	3.021-A	H-19(B)->LUMO(B) (98%)
53	18975.8	527.0	0.005	3.303-A	H-2(A)->LUMO(A) (10%), H-1(A)->L+2(A) (26%), HOMO(A)->L+3(A) (26%)
54	19357.3	516.6	0.0399	3.571-A	H-21(B)->LUMO(B) (28%)
55	19450.1	514.1	4E-4	3.375-A	H-22(B)->LUMO(B) (19%), H-18(B)->LUMO(B) (11%), H-15(B)->L+1(B) (10%)
56	19784.8	505.4	0.0044	3.563-A	H-2(A)->LUMO(A) (75%)
57	19938.0	501.6	0.0693	3.609-A	H-2(A)->L+1(A) (56%), H-21(B)->LUMO(B) (11%)
58	20068.7	498.3	0.0039	3.667-A	H-3(A)->LUMO(A) (88%)
59	20182.4	495.5	6E-4	3.767-A	H-4(A)->LUMO(A) (49%)
60	20304.2	492.5	0.0027	3.527-A	HOMO(A)->L+3(A) (13%)

Table S8. TD DFT calculated electronic transitions for singlet [**3c**]$^{2+}$ (*in vacuo*).

No	Energy (cm^{-1})	Wavelength (nm)	Osc. Strength	Symmetry	Major contributions
1	7012.2	1426.1	0.2272	Singlet-A	H-7->LUMO (30%), HOMO->LUMO (70%)
2	7109.8	1406.5	0.0096	Singlet-A	H-6->LUMO (13%), H-3->LUMO (85%)
3	7264.6	1376.5	5E-4	Singlet-A	H-6->LUMO (81%), H-3->LUMO (13%)
4	7417.9	1348.1	0.0031	Singlet-A	H-1->LUMO (94%)
5	7540.5	1326.2	0.0013	Singlet-A	H-2->LUMO (98%)
6	7871.2	1270.5	2E-4	Singlet-A	H-4->LUMO (99%)
7	8008.3	1248.7	1E-4	Singlet-A	H-5->LUMO (99%)
8	8523.7	1173.2	0.1514	Singlet-A	H-9->LUMO (16%), H-7->LUMO (60%), HOMO->LUMO (23%)
9	8750.3	1142.8	0.0029	Singlet-A	H-8->LUMO (97%)
10	9272.1	1078.5	0.0971	Singlet-A	H-9->LUMO (80%), HOMO->LUMO (10%)
11	10233.6	977.2	0.029	Singlet-A	H-10->LUMO (95%)
12	11170.0	895.3	0.0042	Singlet-A	H-14->LUMO (75%), H-13->LUMO (15%)
13	11449.8	873.4	0.0155	Singlet-A	H-13->LUMO (12%), H-11->LUMO (86%)
14	11570.0	864.3	0.0031	Singlet-A	H-14->LUMO (19%), H-13->LUMO (37%), H-12->LUMO (32%)
15	12065.2	828.8	0.0217	Singlet-A	H-13->LUMO (27%), H-12->LUMO (59%)

16	13354.9	748.8	0.1565	Singlet-A	H-15->LUMO (86%)
17	13893.7	719.8	0.0014	Singlet-A	H-18->LUMO (98%)
18	14147.0	706.9	0.0012	Singlet-A	H-19->LUMO (98%)
19	15197.9	658.0	0.0026	Singlet-A	H-17->LUMO (93%)
20	15617.3	640.3	0.1009	Singlet-A	HOMO->L+1 (95%)
21	15689.1	637.4	4E-4	Singlet-A	H-16->LUMO (98%)
22	17149.8	583.1	0.0709	Singlet-A	HOMO->L+2 (92%)
23	18277.3	547.1	2E-4	Singlet-A	H-20->LUMO (96%)
24	18641.1	536.4	6E-4	Singlet-A	H-1->L+1 (78%), H-1->L+3 (13%)
25	18902.4	529.0	3E-4	Singlet-A	H-2->L+1 (67%), H-2->L+2 (15%), H-2->L+3 (15%)
26	19210.5	520.5	0.0083	Singlet-A	H-3->L+1 (96%)
27	19308.9	517.9	0	Singlet-A	H-4->L+1 (83%), H-4->L+3 (10%)
28	19575.1	510.9	0.0038	Singlet-A	H-5->L+1 (22%), HOMO->L+3 (52%)
29	19590.4	510.5	0.0017	Singlet-A	H-5->L+1 (50%), H-5->L+2 (10%), HOMO->L+3 (22%)
30	19738.0	506.6	0.0049	Singlet-A	H-21->LUMO (19%), H-6->L+1 (60%), H-6->L+2 (10%)
31	19906.6	502.3	0.0192	Singlet-A	H-21->LUMO (16%), H-6->L+2 (71%)
32	20038.8	499.0	0.0099	Singlet-A	H-21->LUMO (49%), H-6->L+1 (21%)
33	20209.8	494.8	0.0018	Singlet-A	H-7->L+1 (94%)
34	20767.2	481.5	0.0197	Singlet-A	H-3->L+2 (94%)
35	21026.1	475.6	0.0053	Singlet-A	H-9->L+2 (15%), H-8->L+1 (36%), H-8->L+2 (27%)
36	21128.5	473.3	0.0211	Singlet-A	H-24->LUMO (17%), H-9->L+1 (25%), H-8->L+1 (13%), H-8->L+2 (17%)
37	21178.5	472.2	0.0092	Singlet-A	H-24->LUMO (48%), H-23->LUMO (21%), H-9->L+1 (12%)
38	21412.4	467.0	1E-4	Singlet-A	H-2->L+1 (12%), H-2->L+2 (84%)
39	21439.8	466.4	2E-4	Singlet-A	H-1->L+2 (84%), H-1->L+3 (10%)
40	21567.3	463.7	6E-4	Singlet-A	H-13->L+7 (13%), H-12->L+7 (29%), HOMO->L+7 (10%)
41	21636.6	462.2	0.0013	Singlet-A	H-14->L+6 (19%), H-13->L+6 (20%)
42	21784.2	459.0	0.0013	Singlet-A	H-7->L+2 (30%), H-5->L+2 (43%)
43	21826.2	458.2	9E-4	Singlet-A	H-7->L+2 (32%), H-5->L+2 (38%)
44	21860.0	457.5	1E-4	Singlet-A	H-4->L+2 (82%)
45	21957.6	455.4	0.0073	Singlet-A	H-9->L+1 (18%), H-9->L+2 (19%), H-8->L+1 (21%), H-8->L+2 (14%)
46	21988.3	454.8	0.0058	Singlet-A	H-23->LUMO (24%)
47	22143.1	451.6	1E-4	Singlet-A	H-17->L+7 (15%), H-14->L+6 (16%)
48	22152.0	451.4	6E-4	Singlet-A	H-2->L+1 (20%), H-2->L+3 (66%), H-2->L+4 (11%)
49	22182.7	450.8	0.0013	Singlet-A	H-1->L+1 (19%), H-1->L+3 (58%)
50	22223.0	450.0	0.0024	Singlet-A	HOMO->L+4 (49%)
51	22282.7	448.8	0.0075	Singlet-A	H-17->L+6 (12%), H-9->L+1 (10%), H-9->L+2 (11%)
52	22373.0	447.0	0.0086	Singlet-A	H-23->LUMO (11%), H-9->L+1 (13%), H-9->L+2 (14%)
53	22577.1	442.9	0.0031	Singlet-A	H-22->LUMO (18%), H-10->L+1 (34%), H-9->L+2 (15%)
54	22668.2	441.1	0.0128	Singlet-A	H-18->L+7 (42%)
55	22715.8	440.2	3E-4	Singlet-A	H-3->L+3 (95%)
56	22758.5	439.4	1E-4	Singlet-A	H-4->L+1 (14%), H-4->L+3 (71%)
57	22775.5	439.1	1E-4	Singlet-A	H-5->L+1 (13%), H-5->L+3 (73%), H-5->L+4 (10%)
58	22856.9	437.5	0.0323	Singlet-A	H-19->L+6 (41%), HOMO->L+4 (10%)
59	23079.6	433.3	0.0119	Singlet-A	H-22->LUMO (53%), H-10->L+1 (10%)
60	23109.4	432.7	0.0248	Singlet-A	H-14->L+1 (20%), H-10->L+2 (25%), HOMO->L+5 (23%)

Table S9. TD DFT calculated electronic transitions for singlet [**3c**]$^{2+}$ (DCM).

No	Energy (cm^{-1})	Wavelength (nm)	Osc. Strength	Symmetry	Major contributions
1	6914.6	1446.2	0.5693	Singlet-A	HOMO->LUMO (97%)
2	8453.5	1182.9	3E-4	Singlet-A	H-2->LUMO (21%), H-1->LUMO (78%)
3	8889.0	1125.0	0.0029	Singlet-A	H-2->LUMO (78%), H-1->LUMO (21%)
4	9189.9	1088.2	0.1124	Singlet-A	H-3->LUMO (85%)
5	9811.7	1019.2	0.0074	Singlet-A	H-4->LUMO (97%)
6	10159.4	984.3	0.0612	Singlet-A	H-5->LUMO (79%)
7	10769.1	928.6	0.0251	Singlet-A	H-8->LUMO (72%)
8	10912.7	916.4	0.0015	Singlet-A	H-13->LUMO (20%), H-12->LUMO (54%)
9	11403.1	877.0	9E-4	Singlet-A	H-11->LUMO (49%), H-7->LUMO (34%)
10	11470.0	871.8	0.0089	Singlet-A	H-11->LUMO (16%), H-7->LUMO (49%), H-6->LUMO (17%)
11	11565.2	864.7	0.0037	Singlet-A	H-8->LUMO (14%), H-6->LUMO (72%)
12	11989.4	834.1	0	Singlet-A	H-10->LUMO (42%), H-9->LUMO (48%)
13	12079.0	827.9	1E-4	Singlet-A	H-10->LUMO (49%), H-9->LUMO (28%)
14	12159.6	822.4	4E-4	Singlet-A	H-14->LUMO (30%), H-12->LUMO (24%), H-11->LUMO (13%), H-9->LUMO (22%)
15	12564.5	795.9	0.065	Singlet-A	H-14->LUMO (37%), H-13->LUMO (52%)
16	12820.2	780.0	1E-4	Singlet-A	H-16->LUMO (96%)

17	12975.8	770.7	0.0022	Singlet-A	H-18->LUMO (94%)
18	13494.5	741.0	0.1261	Singlet-A	H-15->LUMO (89%)
19	14951.9	668.8	0.0048	Singlet-A	H-17->LUMO (93%)
20	15567.3	642.4	0.0996	Singlet-A	HOMO->L+1 (96%)
21	17155.4	582.9	0.089	Singlet-A	HOMO->L+2 (94%)
22	18270.1	547.3	5E-4	Singlet-A	H-20->LUMO (96%)
23	19148.4	522.2	0.0098	Singlet-A	HOMO->L+3 (91%)
24	19511.4	512.5	1E-4	Singlet-A	H-19->LUMO (99%)
25	19931.6	501.7	0.0085	Singlet-A	H-21->LUMO (86%)
26	20925.2	477.9	0.0102	Singlet-A	H-1->L+1 (76%)
27	21058.3	474.9	0.029	Singlet-A	H-3->L+1 (32%), H-2->L+1 (43%)
28	21105.1	473.8	0.0181	Singlet-A	H-3->L+1 (50%), H-2->L+1 (36%)
29	21205.9	471.6	0.0213	Singlet-A	H-2->L+2 (28%), H-1->L+2 (38%)
30	21364.8	468.1	0.0033	Singlet-A	H-11->L+7 (44%), HOMO->L+7 (10%)
31	21434.2	466.5	0.0047	Singlet-A	H-14->L+6 (29%), H-13->L+6 (10%)
32	21521.3	464.7	6E-4	Singlet-A	H-24->LUMO (46%), H-23->LUMO (31%)
33	21718.9	460.4	0.0083	Singlet-A	H-24->LUMO (18%), H-16->L+7 (12%), HOMO->L+4 (13%)
34	21830.2	458.1	0.0036	Singlet-A	H-17->L+7 (16%), H-12->L+7 (11%)
35	21925.4	456.1	0.0258	Singlet-A	H-17->L+6 (15%), HOMO->L+4 (22%)
36	21978.6	455.0	0.022	Singlet-A	H-4->L+1 (38%)
37	21987.5	454.8	0.0124	Singlet-A	H-18->L+6 (29%), H-16->L+7 (24%)
38	22043.9	453.6	0.0411	Singlet-A	H-18->L+6 (14%), H-17->L+6 (10%), H-4->L+1 (10%), HOMO->L+4 (10%)
39	22347.2	447.5	0.0509	Singlet-A	H-23->LUMO (20%), H-5->L+1 (16%), H-4->L+2 (11%)
40	22400.4	446.4	0.0062	Singlet-A	H-23->LUMO (16%), H-5->L+1 (10%), H-4->L+2 (13%), HOMO->L+4 (13%)
41	22430.3	445.8	0.0046	Singlet-A	H-7->L+1 (63%), H-7->L+3 (13%)
42	22575.5	443.0	0.0032	Singlet-A	H-8->L+1 (10%), H-6->L+1 (39%), H-5->L+2 (10%)
43	22625.5	442.0	0.0027	Singlet-A	H-8->L+1 (22%), H-6->L+1 (22%)
44	22724.7	440.1	0.0127	Singlet-A	H-2->L+2 (38%), H-1->L+2 (40%)
45	22909.4	436.5	0.0342	Singlet-A	H-5->L+2 (10%), H-4->L+1 (22%), H-3->L+2 (40%)
46	23052.9	433.8	0.0058	Singlet-A	H-22->LUMO (59%)
47	23136.8	432.2	0.0085	Singlet-A	H-13->L+1 (17%), H-4->L+2 (17%), HOMO->L+5 (14%)
48	23240.9	430.3	0.0015	Singlet-A	H-10->L+1 (39%), H-9->L+1 (31%)
49	23351.4	428.2	0.0014	Singlet-A	H-8->L+2 (14%), H-5->L+1 (25%), H-4->L+2 (14%)
50	23436.1	426.7	0.0011	Singlet-A	H-10->L+1 (22%), H-9->L+1 (37%)
51	23528.0	425.0	0.0027	Singlet-A	H-14->L+1 (13%), HOMO->L+5 (38%)
52	23698.2	422.0	0.0216	Singlet-A	H-8->L+1 (11%), H-5->L+2 (36%), H-3->L+2 (11%)
53	23952.2	417.5	0.0265	Singlet-A	H-22->LUMO (10%), H-11->L+1 (12%)
54	23990.2	416.8	0.0164	Singlet-A	H-16->L+1 (18%), H-16->L+2 (11%), H-2->L+3 (19%), H-1->L+3 (27%)
55	24041.0	416.0	0.0109	Singlet-A	H-16->L+1 (20%), H-16->L+2 (12%), H-2->L+3 (12%), H-1->L+3 (23%)
56	24117.6	414.6	0.0532	Singlet-A	H-26->LUMO (15%), H-25->LUMO (13%), H-16->L+1 (11%), H-8->L+2 (10%)
57	24225.7	412.8	0.0115	Singlet-A	H-25->LUMO (26%), H-3->L+3 (46%)
58	24296.6	411.6	0.0045	Singlet-A	H-18->L+1 (68%), H-18->L+2 (18%)
59	24448.3	409.0	0.1177	Singlet-A	H-12->L+1 (27%), H-2->L+3 (27%)
60	24620.1	406.2	0.0949	Singlet-A	H-25->LUMO (38%), H-3->L+3 (25%)

Table S10. TD DFT calculated electronic transitions for [**3c**$^{\bullet}$]$^{-}$ (*in vacuo*)

No	Energy (cm^{-1})	Wavelength (nm)	Osc. Strength	Symmetry	Major contributions
1	1665.5	6004.1	0.0506	2.034-A	HOMO(A)->LUMO(A) (95%)
2	4090.0	2445.0	0.0967	2.015-A	HOMO(A)->L+1(A) (86%)
3	7410.6	1349.4	0.0279	2.027-A	HOMO(A)->L+2(A) (91%)
4	9629.5	1038.5	1E-4	3.473-A	H-8(A)->L+4(A) (15%), H-7(B)->L+5(B) (15%)
5	9773.8	1023.1	1E-4	3.458-A	--
6	10293.2	971.5	0.0011	3.418-A	H-13(A)->L+4(A) (15%), H-1(A)->LUMO(A) (16%), H-12(B)->L+5(B) (13%), HOMO(B)->LUMO(B) (22%)
7	10357.0	965.5	7E-4	3.436-A	H-13(A)->L+4(A) (29%), H-13(B)->L+5(B) (12%), H-12(B)->L+5(B) (22%), HOMO(B)->LUMO(B) (10%)
8	10428.7	958.9	2E-4	3.290-A	--
9	10641.7	939.7	0.0167	2.162-A	HOMO(A)->L+3(A) (89%)
10	10819.9	924.2	0	3.430-A	H-10(A)->L+4(A) (11%), H-8(A)->L+4(A) (14%), H-7(B)->L+5(B) (15%)
11	10857.8	921.0	1E-4	3.471-A	H-7(A)->L+8(A) (28%), H-7(A)->L+9(A) (20%), H-6(B)->L+9(B) (21%), H-6(B)->L+10(B) (20%)
12	12522.6	798.6	0.0566	2.139-A	H-1(A)->LUMO(A) (34%), HOMO(B)->LUMO(B) (38%), HOMO(B)->L+1(B) (13%), HOMO(B)->L+2(B) (10%)
13	13537.2	738.7	0.0128	3.217-A	H-2(A)->LUMO(A) (15%), H-1(A)->L+1(A) (10%), H-2(B)->LUMO(B)

					(11%), HOMO(B)->LUMO(B) (15%), HOMO(B)->L+1(B) (23%)
14	14126.0	707.9	0.0054	3.188-A	H-1(A)->LUMO(A) (31%), H-1(A)->L+1(A) (10%), HOMO(B)->L+1(B) (19%)
15	14789.8	676.1	0.0136	2.698-A	H-1(A)->L+1(A) (18%), HOMO(B)->L+2(B) (55%)
16	15581.0	641.8	0.0011	3.392-A	H-6(A)->LUMO(A) (25%), H-5(B)->LUMO(B) (28%)
17	16191.6	617.6	0.0516	3.089-A	H-2(A)->LUMO(A) (13%), H-2(A)->L+1(A) (11%), H-1(A)->L+2(A) (16%), HOMO(B)->L+3(B) (10%)
18	16323.0	612.6	0.2175	2.095-A	H-2(A)->LUMO(A) (30%), H-1(A)->L+1(A) (13%), H-1(B)->LUMO(B) (29%)
19	16812.6	594.8	0.2282	2.544-A	H-2(A)->L+1(A) (10%), H-1(A)->L+1(A) (21%), HOMO(B)->L+1(B) (16%), HOMO(B)->L+2(B) (18%)
20	17342.5	576.6	0.0061	2.175-A	HOMO(A)->L+4(A) (74%)
21	17627.2	567.3	0.0083	2.517-A	H-3(A)->LUMO(A) (33%), H-1(B)->L+1(B) (22%), H-1(B)->L+2(B) (19%)
22	17760.3	563.1	0.0094	3.022-A	H-2(B)->LUMO(B) (42%), H-1(B)->LUMO(B) (34%)
23	17782.1	562.4	5E-4	2.328-A	HOMO(A)->L+4(A) (15%), HOMO(A)->L+8(A) (37%), HOMO(A)->L+9(A) (19%)
24	18570.1	538.5	0.014	3.335-A	H-8(A)->LUMO(A) (16%), H-7(B)->LUMO(B) (14%)
25	18662.9	535.8	0.0103	3.204-A	H-8(A)->LUMO(A) (22%), H-7(B)->LUMO(B) (20%), H-4(B)->LUMO(B) (12%)
26	18716.1	534.3	0.0461	3.340-A	H-1(A)->L+2(A) (19%)
27	19236.3	519.8	0.042	2.867-A	H-3(A)->LUMO(A) (22%), H-1(B)->L+1(B) (14%)
28	19346.0	516.9	0.1307	2.564-A	H-3(B)->L+1(B) (14%), H-3(B)->L+2(B) (11%), H-1(B)->L+2(B) (16%)
29	19615.4	509.8	0.0053	2.628-A	H-2(A)->L+1(A) (12%), H-3(B)->LUMO(B) (11%), HOMO(B)->L+3(B) (15%)
30	19948.5	501.3	0.0126	2.430-A	H-5(A)->LUMO(A) (17%), H-4(A)->LUMO(A) (22%)
31	20021.9	499.5	5E-4	2.974-A	H-2(B)->L+1(B) (17%)
32	20180.8	495.5	0.0061	2.795-A	H-6(A)->LUMO(A) (10%), HOMO(A)->L+6(A) (13%), H-1(B)->L+2(B) (10%)
33	20208.2	494.8	0.0136	2.470-A	H-3(B)->LUMO(B) (34%)
34	20261.5	493.5	0.0023	2.131-A	HOMO(A)->L+6(A) (66%)
35	20271.1	493.3	0.0062	2.230-A	H-3(B)->LUMO(B) (12%)
36	20335.7	491.7	0.0045	2.535-A	H-4(A)->LUMO(A) (10%), H-1(A)->L+2(A) (12%), HOMO(B)->L+3(B) (14%), HOMO(B)->L+4(B) (10%)
37	20476.0	488.4	0.0027	2.101-A	H-3(B)->LUMO(B) (12%)
38	20626.0	484.8	0.0136	2.055-A	HOMO(A)->L+8(A) (16%), HOMO(A)->L+9(A) (26%), HOMO(A)->L+10(A) (46%)
39	20749.4	481.9	0.0168	2.262-A	H-4(A)->LUMO(A) (19%), HOMO(A)->L+5(A) (13%), HOMO(A)->L+10(A) (11%), H-3(B)->L+1(B) (10%)
40	20818.8	480.3	0.0074	2.080-A	HOMO(A)->L+5(A) (74%)
41	20870.4	479.1	0.3701	2.236-A	H-7(B)->LUMO(B) (10%), H-4(B)->LUMO(B) (17%)
42	21095.4	474.0	0.021	2.932-A	H-6(A)->L+1(A) (10%), HOMO(A)->L+9(A) (10%), HOMO(A)->L+10(A) (10%), H-5(B)->L+1(B) (10%)
43	21161.6	472.6	0.0297	2.511-A	H-5(A)->LUMO(A) (10%)
44	21266.4	470.2	0.0282	3.265-A	H-27(A)->L+8(A) (11%)
45	21291.4	469.7	0.0134	3.297-A	H-13(A)->LUMO(A) (27%), H-13(B)->LUMO(B) (16%), H-12(B)->LUMO(B) (38%)
46	21328.5	468.9	4E-4	3.466-A	H-34(A)->L+4(A) (31%), H-34(B)->L+5(B) (22%), H-33(B)->L+5(B) (19%)
47	21338.2	468.6	0.0737	2.486-A	H-13(A)->LUMO(A) (10%), H-6(B)->L+1(B) (11%), H-6(B)->L+2(B) (20%)
48	21399.5	467.3	0.0944	2.351-A	H-13(A)->LUMO(A) (12%), H-6(B)->L+2(B) (12%)
49	21477.7	465.6	0.0263	2.932-A	H-4(A)->L+1(A) (16%)
50	21563.2	463.8	0.0138	2.690-A	H-4(B)->L+1(B) (13%)
51	21734.2	460.1	0.0038	2.815-A	H-7(A)->LUMO(A) (87%)
52	21783.4	459.1	0.0555	2.501-A	H-13(A)->LUMO(A) (14%)
53	21927.0	456.1	0.0058	3.073-A	H-6(A)->L+1(A) (13%)
54	22214.1	450.2	0.0092	2.057-A	H-8(A)->L+4(A) (17%), H-7(B)->L+5(B) (17%)
55	22244.8	449.5	0.048	2.440-A	H-2(B)->L+1(B) (10%), H-2(B)->L+2(B) (10%), HOMO(B)->L+4(B) (10%)
56	22295.6	448.5	0.0017	2.062-A	HOMO(A)->L+12(A) (84%)
57	22393.2	446.6	0.0015	2.077-A	HOMO(A)->L+11(A) (71%)
58	22497.2	444.5	0.0016	2.134-A	HOMO(A)->L+7(A) (79%)
59	22513.4	444.2	0.0276	2.608-A	H-5(A)->LUMO(A) (10%)
60	22585.1	442.8	0.0064	2.954-A	

Table S11. TD DFT calculated electronic transitions for triplet [**3c**$^{\bullet\bullet}$]$^{2-}$ (*in vacuo*).

No	Energy (cm^{-1})	Wavelength (nm)	Osc. Strength	Symmetry	Major contributions
1	4653.8	2148.8	0.0482	3.030-A	HOMO(A)->LUMO(A) (92%)

2	5174.9	1932.4	0.0753	3.032-A	H-1(A)->LUMO(A) (87%)
3	8776.9	1139.4	0.0563	3.042-A	HOMO(A)->L+1(A) (89%)
4	9412.5	1062.4	0.0055	3.130-A	H-1(A)->L+1(A) (86%)
5	9506.9	1051.9	1E-4	4.095-A	H-6(B)->L+9(B) (10%)
6	9515.7	1050.9	0	4.072-A	--
7	10428.7	958.9	0	4.030-A	--
8	10444.1	957.5	1E-4	4.027-A	--
9	10786.9	927.1	0	4.142-A	H-10(A)->L+4(A) (14%), H-10(A)->L+7(A) (14%), H-9(B)->L+6(B) (12%), H-9(B)->L+8(B) (13%), H-9(B)->L+9(B) (21%)
10	10790.1	926.8	0	4.145-A	H-9(A)->L+4(A) (10%), H-9(A)->L+6(A) (11%), H-9(A)->L+7(A) (15%), H-8(B)->L+8(B) (18%), H-8(B)->L+9(B) (19%)
11	11940.2	837.5	0.036	3.194-A	HOMO(A)->L+2(A) (76%), HOMO(B)->L+1(B) (14%)
12	12912.9	774.4	1E-4	3.100-A	H-1(A)->L+2(A) (86%)
13	13504.1	740.5	0.0053	3.628-A	H-2(A)->LUMO(A) (24%), HOMO(A)->L+2(A) (20%), HOMO(B)->L+1(B) (41%)
14	13608.2	734.9	0.1066	3.243-A	H-1(A)->L+2(A) (10%), HOMO(B)->LUMO(B) (70%)
15	15459.2	646.9	0.0197	3.405-A	H-2(A)->LUMO(A) (36%), H-1(B)->LUMO(B) (20%), HOMO(B)->L+1(B) (16%), HOMO(B)->L+2(B) (16%)
16	16210.1	616.9	0.0093	3.938-A	H-3(A)->LUMO(A) (39%), H-2(A)->L+1(A) (14%), H-1(B)->L+2(B) (14%)
17	17461.1	572.7	0.0172	3.201-A	H-1(A)->L+7(A) (13%), HOMO(A)->L+4(A) (22%), HOMO(A)->L+6(A) (11%)
18	17532.9	570.4	0.2946	3.162-A	H-2(A)->LUMO(A) (10%), H-1(B)->LUMO(B) (26%), HOMO(B)->L+1(B) (13%)
19	17573.2	569.0	0.2282	3.169-A	H-1(A)->L+4(A) (13%), HOMO(A)->L+7(A) (13%), H-1(B)->LUMO(B) (16%)
20	17699.8	565.0	0.0186	3.207-A	H-2(B)->LUMO(B) (17%), H-1(B)->L+1(B) (46%)
21	17918.4	558.1	0.0744	3.171-A	H-2(B)->L+1(B) (14%), HOMO(B)->L+2(B) (50%)
22	18655.6	536.0	0.0207	3.720-A	H-2(A)->L+1(A) (13%), H-3(B)->L+1(B) (17%), H-2(B)->LUMO(B) (16%), HOMO(B)->L+3(B) (23%)
23	19329.9	517.3	0.0275	3.416-A	H-3(B)->LUMO(B) (20%), H-2(B)->L+1(B) (24%), H-1(B)->LUMO(B) (10%)
24	19454.9	514.0	0.1506	3.642-A	H-4(A)->LUMO(A) (20%), H-3(B)->LUMO(B) (16%), H-2(B)->L+2(B) (16%)
25	19589.6	510.5	0.0227	3.676-A	H-3(A)->LUMO(A) (17%), H-2(A)->L+1(A) (20%), H-3(B)->L+1(B) (15%)
26	19763.0	506.0	0.0318	3.095-A	H-1(A)->L+6(A) (10%), HOMO(A)->L+4(A) (10%), HOMO(A)->L+5(A) (10%), HOMO(A)->L+6(A) (11%)
27	19804.1	504.9	0.0194	3.097-A	H-1(A)->L+6(A) (13%), HOMO(A)->L+5(A) (21%), HOMO(A)->L+6(A) (10%)
28	19838.0	504.1	0.0082	3.202-A	HOMO(A)->L+6(A) (13%), H-6(B)->L+1(B) (14%), H-5(B)->LUMO(B) (20%)
29	20011.4	499.7	0.0065	3.373-A	HOMO(B)->L+3(B) (12%)
30	20192.1	495.2	0.0059	3.159-A	--
31	20292.1	492.8	0.0021	3.214-A	--
32	20400.2	490.2	0.0019	3.052-A	HOMO(A)->L+3(A) (67%), HOMO(A)->L+5(A) (17%)
33	20448.6	489.0	0.1783	3.244-A	H-6(B)->LUMO(B) (11%), H-5(B)->L+1(B) (17%), H-4(B)->LUMO(B) (21%)
34	20663.9	483.9	0.0159	3.424-A	H-2(A)->L+1(A) (28%), HOMO(B)->L+3(B) (19%), HOMO(B)->L+4(B) (17%)
35	20923.6	477.9	0.0386	3.573-A	H-5(A)->LUMO(A) (19%), H-3(A)->LUMO(A) (11%)
36	21047.0	475.1	0.1369	3.279-A	H-4(A)->LUMO(A) (11%), H-6(B)->LUMO(B) (11%), H-4(B)->LUMO(B) (27%)
37	21066.4	474.7	0.0105	4.071-A	H-32(B)->L+9(B) (11%)
38	21076.9	474.5	0.0044	4.087-A	H-31(B)->L+9(B) (12%)
39	21161.6	472.6	0.024	3.092-A	H-1(A)->L+3(A) (21%), HOMO(A)->L+8(A) (15%), H-4(B)->L+1(B) (11%)
40	21255.1	470.5	0.0071	3.064-A	H-1(A)->L+3(A) (16%), H-1(A)->L+8(A) (23%), HOMO(A)->L+8(A) (36%)
41	21330.9	468.8	0.0031	3.129-A	H-1(A)->L+3(A) (27%), HOMO(A)->L+9(A) (14%)
42	21375.3	467.8	0.0092	3.076-A	H-1(A)->L+9(A) (27%), HOMO(A)->L+9(A) (45%)
43	21564.8	463.7	0.0294	3.586-A	H-7(A)->LUMO(A) (18%), H-5(A)->LUMO(A) (10%)
44	21708.4	460.7	0.0878	3.426-A	H-6(A)->LUMO(A) (12%), HOMO(A)->L+10(A) (23%)
45	21802.0	458.7	0.0331	3.194-A	HOMO(A)->L+10(A) (17%), H-9(B)->LUMO(B) (17%), H-9(B)->L+1(B) (11%)
46	21863.3	457.4	0.0043	3.148-A	H-8(B)->LUMO(B) (29%), H-8(B)->L+1(B) (24%)
47	21890.7	456.8	0.0016	3.458-A	H-6(A)->LUMO(A) (25%), H-9(B)->LUMO(B) (10%)
48	22037.5	453.8	0.0042	3.082-A	H-1(A)->L+5(A) (12%), HOMO(A)->L+4(A) (25%)
49	22179.4	450.9	0.0291	3.209-A	H-3(B)->LUMO(B) (32%), H-2(B)->L+1(B) (30%)
50	22205.2	450.3	0.1183	3.663-A	H-6(A)->LUMO(A) (10%), H-4(A)->LUMO(A) (15%), H-3(A)->L+1(A) (36%)
51	22276.2	448.9	0.0162	3.464-A	H-1(B)->L+2(B) (47%)
52	22409.3	446.2	0.0116	3.584-A	H-8(A)->LUMO(A) (13%), H-7(A)->LUMO(A) (15%), H-3(B)->L+1(B)

					(11%)
53	22451.2	445.4	0.0255	3.202-A	H-1(A)->L+10(A) (16%), HOMO(A)->L+10(A) (11%)
54	22495.6	444.5	0.023	3.129-A	H-1(A)->L+10(A) (12%), HOMO(A)->L+10(A) (20%)
55	22595.6	442.6	9E-4	3.076-A	HOMO(A)->L+11(A) (75%)
56	22723.1	440.1	0.0011	3.419-A	--
57	22793.2	438.7	0.0017	3.202-A	--
58	22917.4	436.3	9E-4	3.107-A	H-1(A)->L+4(A) (28%)
59	22982.8	435.1	0.0109	3.341-A	H-8(A)->LUMO(A) (10%), H-2(A)->L+2(A) (15%)
60	23134.4	432.3	3E-4	3.072-A	H-1(A)->L+10(A) (13%), HOMO(A)->L+12(A) (20%)

Table S12. TD DFT calculated electronic transitions for triplet [**3c**]$^{2-}$ (*in vacuo*).

No	Energy (cm^{-1})	Wavelength (nm)	Osc. Strength	Symmetry	Major contributions
1	2251.9	4440.7	0.065	Singlet-A	HOMO->LUMO (135%), HOMO->L+1 (17%)
2	5716.1	1749.5	0.3742	Singlet-A	HOMO->LUMO (29%), HOMO->L+1 (84%)
3	8379.3	1193.4	0.0234	Singlet-A	HOMO->L+2 (99%)
4	10682.8	936.1	0.0372	Singlet-A	HOMO->L+3 (99%)
5	13442.0	743.9	0.0118	Singlet-A	H-1->LUMO (96%)
6	15659.3	638.6	0.0608	Singlet-A	H-2->LUMO (94%)
7	16363.4	611.1	0.0025	Singlet-A	HOMO->L+7 (76%)
8	16740.0	597.4	0.0012	Singlet-A	HOMO->L+10 (61%), HOMO->L+12 (17%)
9	17352.2	576.3	0.478	Singlet-A	H-1->L+1 (86%)
10	17597.4	568.3	1E-4	Singlet-A	HOMO->L+4 (95%)
11	18166.8	550.5	0.0083	Singlet-A	HOMO->L+5 (73%), HOMO->L+6 (21%)
12	18208.8	549.2	0.0099	Singlet-A	HOMO->L+5 (20%), HOMO->L+6 (71%)
13	19094.4	523.7	0.1612	Singlet-A	H-5->LUMO (20%), H-4->LUMO (30%), H-3->LUMO (22%)
14	19257.3	519.3	0.519	Singlet-A	H-4->LUMO (10%), H-3->LUMO (52%)
15	19413.0	515.1	0.0245	Singlet-A	HOMO->L+8 (77%)
16	19461.4	513.8	0.0297	Singlet-A	H-4->LUMO (26%), H-2->L+1 (16%), H-1->L+2 (24%), HOMO->L+8 (12%)
17	19532.3	512.0	0.0169	Singlet-A	HOMO->L+9 (92%)
18	19596.0	510.3	0.0242	Singlet-A	HOMO->L+11 (94%)
19	19744.5	506.5	0.0036	Singlet-A	H-5->LUMO (27%), HOMO->L+10 (12%), HOMO->L+12 (47%)
20	20372.8	490.9	0.0052	Singlet-A	H-6->LUMO (24%)
21	20426.8	489.6	0.0057	Singlet-A	H-6->LUMO (11%), H-1->L+2 (19%)
22	20529.2	487.1	0.0117	Singlet-A	HOMO->L+13 (63%), HOMO->L+14 (22%)
23	20636.5	484.6	9E-4	Singlet-A	H-6->LUMO (21%), HOMO->L+14 (29%)
24	20655.1	484.1	0.0032	Singlet-A	H-2->L+1 (16%), H-1->L+2 (18%), HOMO->L+14 (17%)
25	20734.1	482.3	0.0589	Singlet-A	H-6->LUMO (21%), HOMO->L+13 (21%), HOMO->L+14 (30%)
26	20977.7	476.7	0.0285	Singlet-A	H-8->LUMO (17%), H-7->LUMO (28%), H-5->LUMO (18%)
27	21254.3	470.5	0.0128	Singlet-A	H-9->LUMO (15%), H-8->LUMO (61%)
28	21362.4	468.1	0.0036	Singlet-A	H-9->LUMO (67%)
29	21886.7	456.9	0.1038	Singlet-A	H-7->LUMO (32%), H-4->LUMO (11%), H-2->L+1 (22%), H-1->L+3 (13%)
30	22186.7	450.7	6E-4	Singlet-A	HOMO->L+15 (95%)
31	22515.8	444.1	0.0629	Singlet-A	H-10->LUMO (48%), H-9->L+7 (11%)
32	22628.7	441.9	0.0097	Singlet-A	H-9->L+7 (11%), H-8->L+10 (14%), H-5->L+1 (11%), H-4->L+1 (15%)
33	22786.8	438.9	0.0388	Singlet-A	H-9->L+7 (10%), H-2->L+2 (44%)
34	23203.8	431.0	0.0329	Singlet-A	H-3->L+1 (13%), HOMO->L+16 (18%)
35	23289.3	429.4	0.0642	Singlet-A	H-10->LUMO (24%), H-2->L+2 (26%), HOMO->L+16 (20%)
36	23449.0	426.5	4E-4	Singlet-A	H-5->L+7 (16%)
37	23552.2	424.6	0.0418	Singlet-A	H-3->L+1 (57%)
38	23824.8	419.7	0.0353	Singlet-A	H-5->L+1 (18%), H-1->L+3 (67%)
39	23878.0	418.8	0.0132	Singlet-A	H-9->L+7 (10%), H-4->L+1 (32%)
40	23994.2	416.8	0.0016	Singlet-A	HOMO->L+16 (45%)
41	24822.5	402.9	0.2881	Singlet-A	H-6->L+1 (54%)
42	25033.8	399.5	0.0036	Singlet-A	H-8->L+1 (76%), H-8->L+2 (15%)
43	25155.6	397.5	0.0234	Singlet-A	H-9->L+1 (73%), H-9->L+2 (14%)
44	25204.8	396.7	0.3266	Singlet-A	H-5->L+1 (41%), H-4->L+1 (31%)
45	25545.2	391.5	0.0011	Singlet-A	H-11->LUMO (92%)
46	25778.3	387.9	0.003	Singlet-A	H-13->LUMO (50%), H-12->LUMO (28%)
47	25925.9	385.7	0.006	Singlet-A	H-10->L+1 (18%), H-5->L+2 (13%), H-2->L+3 (58%)
48	26119.5	382.9	0.0165	Singlet-A	H-13->LUMO (11%), H-7->L+1 (28%), H-3->L+2 (42%)
49	26582.4	376.2	0.162	Singlet-A	H-13->LUMO (15%), H-7->L+1 (15%), H-3->L+2 (43%)
50	26703.4	374.5	0.0035	Singlet-A	H-13->LUMO (16%), H-12->LUMO (56%)
51	26924.4	371.4	3E-4	Singlet-A	H-14->LUMO (80%)
52	27065.5	369.5	0.0149	Singlet-A	H-4->L+2 (81%)
53	27327.7	365.9	0.034	Singlet-A	H-10->L+1 (30%), H-2->L+3 (20%)
54	27512.4	363.5	0.0025	Singlet-A	H-16->LUMO (83%)

55	27601.9	362.3	0.0146	Singlet-A	H-15->LUMO (91%)
56	27809.2	359.6	4E-4	Singlet-A	HOMO->L+17 (98%)
57	28134.2	355.4	0.274	Singlet-A	H-10->L+1 (26%), H-5->L+2 (57%)
58	28196.3	354.7	0.0016	Singlet-A	H-28->L+7 (12%), H-28->L+10 (22%)
59	28224.6	354.3	0.0035	Singlet-A	H-31->L+7 (30%), H-28->L+7 (14%)
60	28517.3	350.7	0.0165	Singlet-A	H-6->L+2 (80%)

Table S13. Computational details for the optimized structures of compounds.

**Structure / Name**[a]	**SCF E a.u.**	**ZPV**[b] **a.u.**	**lowest freq. cm^{-1}**	**E a.u.**	**ΔH a.u.**	**ΔG**[c] **a.u.**
**3c**/4c-OF_a	-7049.378365	-7048.057971	8.67	-7047.971814	-7047.970870	-7048.188068
[**3c**$^{\cdot}$]$^{+}$/4c+-OF_a	-7049.173539	-7047.854125	8.55	-7047.767580	-7047.766636	-7047.986246
[**3c**]$^{2+}$ /cs-4c++-OF_a	-7048.875844	-7047.555612	9.25	-7047.469165	-7047.468221	-7047.685878
[**3c**]$^{2+}$/cs-4c++-OF_b	-7048.349861	-7047.027637	8.46	-7046.940794	-7046.939850	-7047.160821
[**3c**]$^{2+}$/s-4c++-OF_a	-7048.875844	-7047.555628	9.23	-7047.469169	-7047.468224	-7047.685985
[**3c**]$^{2+}$/s-4c++-OF_b	-7048.364765	-7047.043275	8.18	-7046.956386	-7046.955442	-7047.177185
[**3c**$^{\cdot\cdot}$]$^{2+}$/t-4c++-OF_a	-7048.891868	-7047.572261	7.33	-7047.485886	-7047.484942	-7047.703798
[**3c**$^{\cdot\cdot}$]$^{2+}$/t-4c++-OF_b	-7048.362565	-7047.041241	7.36	-7046.954332	-7046.953388	-7047.176431
[**3c**$^{\cdot}$]$^{-}$/4c--OF_a	-7049.433773	-7048.117371	7.83	-7048.030929	-7048.029985	-7048.248689
[**3c**$^{\cdot\cdot}$]$^{2-}$/t-4c---OF_a	-7049.435684	-7048.122155	9.84	-7048.035916	-7048.034972	-7048.250351
[**3c**]$^{2-}$/cs-4c---OF_a	-7049.386402	-7048.072181	10.04	-7047.986045	-7047.985101	-7048.199133

[a] Data set name (Cartesian coordinated available as *.pdb files). [b] Zero-point vibrational energy. [c] Gibbs free energy.

References

[1] Sheldrick, G. M., *Acta Crystallogr. A* **2015**, *71*, 3.

[2] Dolomanov, O.V., Bourhis, L.J., Gildea, R.J., Howard, J.A.K., Puschmann, H., *J. Appl. Cryst.*, **2009**, *42*, 339.

[3] Gaussian 16, Revision C.01, Frisch, M. J.; Trucks, G. W.; Schlegel, H. B.; Scuseria, G. E.; Robb, M. A.; Cheeseman, J. R.; Scalmani, G.; Barone, V.; Petersson, G. A.; Nakatsuji, H.; Li, X.; Caricato, M.; Marenich, A. V.; Bloino, J.; Janesko, B. G.; Gomperts, R.; Mennucci, B.; Hratchian, H. P.; Ortiz, J. V.; Izmaylov, A. F.; Sonnenberg, J. L.; Williams-Young, D.; Ding, F.; Lipparini, F.; Egidi, F.; Goings, J.; Peng, B.; Petrone, A.; Henderson, T.; Ranasinghe, D.; Zakrzewski, V. G.; Gao, J.; Rega, N.; Zheng, G.; Liang, W.; Hada, M.; Ehara, M.; Toyota, K.; Fukuda, R.; Hasegawa, J.; Ishida, M.; Nakajima, T.; Honda, Y.; Kitao, O.; Nakai, H.; Vreven, T.; Throssell, K.; Montgomery, J. A., Jr.; Peralta, J. E.; Ogliaro, F.; Bearpark, M. J.; Heyd, J. J.; Brothers, E. N.; Kudin, K. N.; Staroverov, V. N.; Keith, T. A.; Kobayashi, R.; Normand, J.; Raghavachari, K.; Rendell, A. P.; Burant, J. C.; Iyengar, S. S.; Tomasi, J.; Cossi, M.; Millam, J. M.; Klene, M.; Adamo, C.; Cammi, R.; Ochterski, J. W.; Martin, R. L.; Morokuma, K.; Farkas, O.; Foresman, J. B.; Fox, D. J. Gaussian, Inc., Wallingford CT, 2016.

[4] Chai, J.-D.; Head-Gordon, M., *J. Chem. Phys.*, **2008**, *10*, 6615.

[5] Yanai, T., Tew, D. P., Handy, N. C., *Chem. Phys. Lett.*, **2004**, *393*, 51.

[6] Grimme, S., Ehrlich, S., Goerigk, L., *J. Comput. Chem.* **2011**, *32*, 1456.

[7] Becke, A. D., *J. Chem. Phys.*, **1993**, *98*, 5648.

[8] Lee, C. Yang, W., Parr, R. G., *Phys. Rev. B*, **1988**, *37*, 785.

[9] O'Boyle, N. M., Tenderholt, A. L., Langner, K. M. , *J. Comp. Chem.* **2008**, *29*, 839.

[10] NBO Version 3.1, E. D. Glendening, A. E. Reed, J. E. Carpenter, and F. Weinhold

[11] Deng, Z., Li, X., Stępień, M., Chmielewski, P. J., *Chem. – Eur. J.*, **2016**, *22*, 4231.
